# Association between achieving adequate antenatal care and health-seeking behaviors: A study of Demographic and Health Surveys in 47 low- and middle-income countries

**DOI:** 10.1371/journal.pmed.1004421

**Published:** 2024-07-05

**Authors:** Boshen Jiao, Isabelle Iversen, Ryoko Sato, Clint Pecenka, Sadaf Khan, Ranju Baral, Margaret E. Kruk, Catherine Arsenault, Stéphane Verguet

**Affiliations:** 1 Department of Global Health and Population, Harvard T.H. Chan School of Public Health, Boston, Massachusetts, United States of America; 2 PATH, Seattle, Washington, United States of America; 3 Department of Global Health, Milken Institute School of Public Health, George Washington University, Washington, DC, United States of America

## Abstract

**Background:**

Antenatal care (ANC) is essential for ensuring the well-being of pregnant women and their fetuses. This study models the association between achieving adequate ANC and various health and health-seeking indicators across wealth quintiles in low- and middle-income countries (LMICs).

**Methods and findings:**

We analyzed data from 638,265 women across 47 LMICs using available Demographic and Health Surveys from 2010 to 2022. Via multilevel logistic regression analyses adjusted for a series of confounding variables and country and wealth quintile fixed effects, we estimated the projected impact of achieving adequate ANC utilization and quality on a series of health and health care indicators: facility birth, postnatal care, childhood immunizations, and childhood stunting and wasting. Achieving adequate levels of ANC utilization and quality (defined as at least 4 visits, blood pressure monitoring, and blood and urine testing) was positively associated with health-seeking behavior across the majority of countries. The strongest association was observed for facility birth, followed by postnatal care and child immunization. The strength of the associations varied across countries and wealth quintiles, with more significant ones observed in countries with lower baseline ANC utilization levels and among the lower wealth quintiles. The associations of ANC with childhood stunting and wasting were notably less statistically significant compared to other indicators. Despite rigorous adjustments for potential confounders, a limitation to the methodology is that it is possible that unobserved variables may still impact outcomes.

**Conclusions:**

Strengthening ANC is associated with improved use of other health care in LMICs. ANC could serve as a critical platform for improving health outcomes for mothers and their children, emphasizing its importance beyond direct impact on maternal and neonatal mortality.

## Abstract 2

Leveraging data from demographic health surveys across the globe, Boshen Jiao and co-authors explore how adequate antenatal care influences health-seeking behaviors more generally.

## Author summary

**Why was this study done?:**

- Antenatal care is essential to the well-being of pregnant women and their fetuses.
- Previous research shows that the frequency of antenatal care visits can significantly influence health behaviors of both mothers and their children.
- Existing studies center on a limited set of countries, leading to a notable gap in our understanding of the potential broader health system impact that antenatal care can have.

**What did the researchers do and find?:**

- We modeled the association between achieving adequate antenatal care and various health indicators across wealth quintiles in 47 low- and middle-income countries using data from Demographic and Health Surveys collected between 2010 and 2022.
- The model estimated the projected impact of achieving adequate antenatal care utilization and quality on facility birth, postnatal care, childhood immunizations, and childhood stunting and wasting.
- We found that achieving adequate levels of antenatal care utilization and quality was positively associated with health-seeking behavior of mothers across most countries.
- The stronger associations were observed in countries with lower baseline antenatal care utilization levels and among lower wealth quintiles.

**What do these findings mean?:**

- Strengthening antenatal care could potentially improve the use of other health care in low- and middle- income countries.
- Antenatal care could serve as a critical platform for improving broader health outcomes for mothers and their children, emphasizing its importance beyond the direct impact on maternal and neonatal health.
- Despite rigorous adjustments for potential confounders, a limitation of the methodology is the possibility that unobserved variables may still impact the outcomes.

## Introduction

Antenatal care (ANC) plays a crucial role in ensuring the well-being of mothers and fetuses during pregnancy. By closely monitoring pregnant women to detect potential complications early and provide timely interventions, ANC serves as a critical platform for achieving positive health outcomes, including potentially major reductions in neonatal and infant mortality rates [1]. The scope of ANC includes assessing fetal health, screening and managing maternal infections and noncommunicable conditions, educating on danger signs and birth preparedness, and offering micronutrient supplementation and immunizations [2]. In 2016, the World Health Organization (WHO) updated its guidelines, recommending a minimum of 8 ANC visits to reduce perinatal deaths and boost maternal satisfaction, regardless of the resource setting [3]. Visit attendance in many places even falls short of the previous 2003 WHO-endorsed Focused Antenatal Care Model [4], which prescribes at least 4 ANC visits during pregnancy [3].

In addition to the direct impact on maternal and infant outcomes, extensive research has shown that the frequency of ANC visits in low- and middle-income countries (LMICs) can significantly influence subsequent health behaviors of both mothers and their children. Women who receive ANC are more likely to adopt health-promoting practices, including choosing institutional birth [5,6], seeking postnatal care (PNC) [7,8], and ensuring their children receive key immunizations [9,10]. These aspects of health care play a pivotal role in promoting the well-being of children—effectively reducing mortality [11–13] and morbidity outcomes like stunting and wasting [14,15]. Most existing studies, however, have centered on a limited set of countries, leading to notable gaps in our comprehension of the potential global impact of ANC and its varying influence across nations.

Moreover, available evidence (though limited) suggests that ANC quality is as important as frequency of ANC usage to improving women’s use of other health care [16–19]. WHO highlights 4 key areas that must be addressed to ensure high-quality ANC: nutritional interventions, maternal and fetal assessments, preventive measures and interventions for common physiological symptoms [3]. Simply increasing the number of ANC visits without ensuring high quality of care may not achieve the desired impact. High-quality ANC ensures that the health and nutritional needs of both the mother and fetus are adequately addressed, setting the foundation for optimal growth and development after birth [20]. Examining the impact of ANC utilization and quality jointly is, therefore, essential.

The third Sustainable Development Goal stresses the importance of equitable access to health care and achieving better health outcomes for all [21]. A number of studies have consistently pointed to the existence of systemic inequalities in health care coverage and quality in LMICs [22,23]. Addressing these inequities is crucial, not only for achieving adequate levels of ANC utilization and quality, but also for narrowing the existing health disparities between and within LMICs.

This study aims to assess the associations between ANC utilization and quality and various health and health care indicators in LMIC settings. We focus on subsequent facility birth, childhood immunizations, and PNC utilization. We also assess associations with levels of childhood stunting and wasting.

## Methods

### Data sources

We sourced publicly available Demographic and Health Surveys (DHS) [24]. The DHS program collects information using standardized survey questionnaires, biomarker collection, and geographic information. DHS enable cross-country comparisons [24]. They provide nationally representative samples for a large number of countries that are comparable and of consistently high quality over time. The sample size typically ranges from 5,000 to 30,000 households including women 15 to 49 and men 15 to 54 years of age. DHS are typically conducted every 5 years.

DHS aim to cover a wide range of population, health, and nutrition topical areas [24]. Therein, we relied on the births recode data, which contain complete birth histories of all interviewed women, including information on pregnancy and postnatal care and health and nutrition indicators for children born within 5 years preceding the survey. To capture the most recent patterns in ANC and health-seeking behaviors, we used 2010 to 2022 standard DHS. Our study sample ultimately consisted of women giving birth within the 5 years preceding the survey. We excluded samples that lacked the independent and dependent variables described in the following sections. A total of 47 countries and 78 DHS were retained (S1 Table lists all countries and DHS included in the analysis).

### Independent variables

ANC utilization is assessed based on the total number of visits by a skilled provider. To evaluate ANC quality, we considered various components of ANC, including blood pressure monitoring, blood testing, urine testing, height and weight measurement, tetanus vaccination, counseling on pregnancy complications, and iron supplementation. Note, however, that measurement of these ANC components was not consistent across countries. Only 3 components remained consistent across all countries: blood pressure monitoring, urine testing, and blood testing. To ensure comparability across all countries, we drew from the previous work by Arsenault and colleagues [16] and focused on these 3 components as proxy indicators of ANC quality. We then created a continuous ANC quality score ranging from zero to one, computed by dividing the number of different care components provided to each woman by the 3 care components assessed [16]. It is important to recognize that these indicators, while essential, do not encompass the full spectrum of ANC and thus provide a somewhat restricted perspective on overall quality. Nevertheless, these indicators are recognized by WHO as fundamental to ANC and are vital for detecting various pregnancy-related risks, such as anemia, hypertension, infections, pre-eclampsia, and nutritional deficiencies [16].

### Dependent variables

In a first analysis, we investigated the impact of ANC utilization and quality on certain outcome variables related to subsequent uptake of health care. These variables included facility birth, diphtheria-pertussis-tetanus third dose (DPT3) vaccination, measles vaccination, and PNC. Facility birth referred to births that occurred either in a public or private health facility as opposed to home. PNC was defined as the newborn receiving a postnatal check within 2 months of birth. DPT3 vaccination corresponded to whether a child 12 to 23 months of age received the 3 doses of the combined DPT vaccine. Measles vaccination corresponded to whether a child 12 to 23 months of age received the first dose of measles (or measles-containing) vaccine in the recommended series.

In a secondary analysis, we examined the outcomes of stunting and wasting among living children born 0 to 59 months before the survey, postulating that a positive pregnancy experience could enhance care uptake for children. Stunting was defined as a height-for-age Z-score falling below minus 2 standard deviations (SD) from the median of the WHO reference population [25]. Wasting was defined as a weight-for-height Z-score below minus 2 SD from the median of the WHO reference population [25]. We excluded the birth weight outcome from the analysis due to significant data gaps.

To account for potential confounding factors, we adjusted for certain sociodemographic variables. These included the mother’s age at the time of childbirth; mother’s education level (no education, primary education, secondary education, and higher education); mother’s marital status (never in union, married or living with a partner, and widowed, divorced or separated); rural or urban residency; mother’s literacy (cannot read at all, can read only parts of a sentence or whole sentence and not ascertained); and child’s birth order and sex. We also accounted for the mother’s body mass index as a proxy for health, recognizing that health could act as a confounding factor.

### Statistical analysis

We conducted a multilevel logistic regression analysis to investigate the impact of ANC on the aforementioned outcome variables. The model comprised key predictor variables including number of ANC visits, ANC quality score and country and wealth quintile fixed effects, while adjusting for confounding variables. To account for the potential heterogeneity of the impacts of ANC utilization and quality across countries and wealth quintiles [26,27], we initially constructed a saturated regression model for each outcome, by including all possible interaction terms between ANC variables, country and wealth quintile. Additionally, we incorporated the squared term of ANC visits to capture the potential nonlinear relationship between the number of visits and outcome. Given the saturated model’s extensive number of independent variables, there was a high risk of overfitting. To mitigate this issue, we used the least absolute shrinkage and selection operator (LASSO) technique with 5-fold cross-validation to discern which interaction terms and whether the squared term of ANC visits was pivotal for our final model. The LASSO method can refine the model by imposing penalties on less important variables, excluding certain interaction terms or the squared term for ANC visits if deemed non-essential, thereby improving the model’s predictive accuracy.

We employed the fitted multilevel logistic regression model to predict outcomes under 2 scenarios: either (1) a baseline reflecting the current state of ANC as observed in the DHS; and (2) a counterfactual intervention scenario where all individuals receive adequate ANC, characterized by at least 4 visits and a perfect quality score. This four-visit threshold draws from the standard established by WHO’s 2003 Focused Antenatal Care Model [3]. While aware of WHO’s 2016 updated recommendation for a minimum of 8 visits, we note that many regions have yet to meet even the previous four-visit standard [4]. Furthermore, a substantial portion of the DHS data was collected before the 2016 update to WHO’s guidelines (S1 Table).

To facilitate this analysis, we employed the method of recycled predictions [28]. This approach recalculates predicted outcomes for each individual in the dataset under both scenarios, enabling a direct comparison of outcomes across the entire population. For the baseline scenario, predictions were made using the existing dataset, thereby mirroring the current conditions of ANC utilization and quality. In contrast, for the counterfactual intervention scenario, we modified the dataset to simulate an increase in the number of ANC visits to 4 for those with fewer than 4 visits and elevated the ANC quality score to one for individuals with a baseline score below one, keeping all other independent variables constant. The differences in projected outcomes between these 2 scenarios would represent the potential changes associated with achieving adequate ANC levels. This analysis was conducted across different wealth quintiles and countries. Sampling weights were incorporated to ensure that the results are representative. The confidence intervals for the estimates were computed using *n* = 1,000 bootstrap replications. S1 Fig illustrates the statistical analysis process.

All computations were conducted using Rstudio (Version 2023.06.01) (www.r-project.org).

This study is reported as per the Strengthening the Reporting of Observational Studies in Epidemiology (STROBE) guidelines (Checklist S1).

## Results

The final analytical sample consisted of 638,265 women from 47 countries with available 2010 to 2022 DHS from 78 standard surveys. Table 1 summarizes descriptive statistics at the country and wealth quintile levels for both ANC and the outcome variables examined. Across country and wealth quintiles, the mean number of ANC visits was 4.6 (interquartile range [IQR]: 3.3, 5.7) and the mean ANC quality score was 0.72 (IQR: 0.56, 0.93). Generally, the richest and richer quintiles exhibited higher baseline ANC visits and quality scores compared to the poorer and poorest quintiles. A total of 59,459 individuals were excluded from the analysis due to incomplete data, with the largest subset of these exclusions (48,904) attributed to missing information on ANC utilization and quality. Individuals with missing ANC data were characterized by older age, lower level of education, a higher proportion of rural residency, and a higher prevalence of obesity relative to those included in the study (Tables 2 and S3).

**Table 1:** Mean and interquartile range of country- and quintile-specific antenatal care (ANC) and outcome indicators in 47 countries, 2010–2022.

	Overall	Poorest quintile	Poorer quintile	Middle quintile	Richer quintile	Richest quintile
Number of ANC visits	4.6 (3.3, 5.7)	3.7 (2.5, 4.8)	4.1 (3.1, 4.9)	4.5 (3.4, 5.2)	5.0 (3.7, 6.0)	5.9 (4.3, 6.7)
ANC quality score*	0.72 (0.56, 0.93)	0.59 (0.38, 0.82)	0.65 (0.42, 0.88)	0.71 (0.53, 0.91)	0.78 (0.63, 0.95)	0.87 (0.83, 0.97)
Facility birth	70% (56%, 92%)	51% (29%, 69%)	61% (45%, 81%)	69% (59%, 86%)	79% (74%, 94%)	90% (87%, 97%)
DPT3 vaccination	76% (66%, 90%)	67% (51%, 87%)	73% (62%, 89%)	77% (68%, 90%)	80% (73%, 92%)	84% (79%, 94%)
Measles vaccination	77% (70%, 89%)	69% (56%, 84%)	73% (64%, 87%)	77% (72%, 88%)	80% (73%, 90%)	85% (82%, 92%)
PNC utilization	50% (32%, 69%)	45% (28%, 63%)	48% (29%, 67%)	50% (33%, 67%)	52% (34%, 70%)	57% (39%, 74%)
Stunting rate**	29% (20%, 38%)	38% (32%, 42%)	33% (26%, 40%)	30% (23%, 37%)	26% (19%, 32%)	18% (13%, 23%)
Waste rate**	9% (5%, 12%)	11% (6%, 16%)	9% (5%, 13%)	9% (5%, 12%)	8% (4%, 11%)	7% (3%, 10%)

Data are from Demographic and Health Surveys conducted in 47 countries from 2010–2022.

* ANC quality score was computed by dividing the number of ANC components (blood pressure monitoring, urine testing, and blood testing) provided to each woman by the total number of ANC components (i.e., 3) in a specific country.

** Stunting was defined as a height-for-age Z-score falling below minus 2 SD from the median of the WHO reference population. Wasting was defined as a weight-for-height Z-score below minus 2 SD from the median of the WHO reference population.

ANC, antenatal care; DPT3, diphtheria-pertussis-tetanus third dose; IQR, interquartile range; PNC, postnatal care; SD, standard deviation; WHO, World Health Organization.

**Table 2:** Distribution of demographic and health variables (presented as *n* or mean (%), number or mean and proportion of participants) for the sample included in the analysis.

	Poorest	Poorer	Middle	Richer	Richest	Total
**Age at childbirth**						
<25 years	70,820 (48.0%)	71,552 (51.3%)	64,621 (50.5%)	57,356 (48.1%)	41,657 (40.1%)	306,006 (47.9%)
25–34 years	59,080 (40.0%)	54,199 (38.9%)	51,557 (40.3%)	51,580 (43.3%)	53,336 (51.3%)	269,752 (42.3%)
>34 years	17,766 (12.0%)	13,617 (9.8%)	11,883 (9.3%)	10,318 (8.7%)	8,923 (8.6%)	62,507 (9.8%)
**Education**						
No education	65,224 (44.2%)	41,503 (29.8%)	28,159 (22.0%)	18,834 (15.8%)	8,386 (8.1%)	162,106 (25.4%)
Primacy	43,861 (29.7%)	39,127 (28.1%)	31,880 (24.9%)	24,358 (20.4%)	14,118 (13.6%)	153,344 (24.0%)
Secondary	36,859 (25.0%)	53,547 (38.4%)	58,161 (45.4%)	58,942 (49.4%)	48,634 (46.8%)	256,143 (40.1%)
Higher	1,722 (1.2%)	5,191 (3.7%)	9,861 (7.7%)	17,120 (14.4%)	32,778 (31.5%)	66,672 (10.4%)
**Marital status**						
Never in union	3,826 (2.6%)	3,929 (2.8%)	4,072 (3.2%)	3,963 (3.3%)	3,792 (3.6%)	19,582 (3.1%)
Married/living with partner	135,888 (92.0%)	129,117 (92.6%)	118,322 (92.4%)	110,378 (92.6%)	96,084 (92.5%)	589,789 (92.4%)
Windowed/divorced/separated	7,952 (5.4%)	6,322 (4.5%)	5,667 (4.4%)	4,913 (4.1%)	4,040 (3.9%)	28,894 (4.5%)
**Place of residence**						
Urban	11,006 (7.5%)	15,494 (11.1%)	29,461 (23.0%)	52,301 (43.9%)	74,645 (71.8%)	182,907 (28.7%)
Rural	136,660 (92.5%)	123,874 (88.9%)	98,600 (77.0%)	66,953 (56.1%)	29,271 (28.2%)	455,358 (71.3%)
**Literacy**						
Cannot real at all	82,748 (56.0%)	56,010 (40.2%)	39,020 (30.5%)	25,731 (21.6%)	11,536 (11.1%)	215,045 (33.7%)
Able to read	63,866 (43.3%)	82,694 (59.3%)	88,485 (69.1%)	93,102 (78.1%)	92,140 (88.7%)	420,287 (65.8%)
Not ascertained	1,052 (0.7%)	661 (0.5%)	556 (0.4%)	421 (0.4%)	239 (0.2%)	2,929 (0.5%)
**Birth order**	3.367 (2.268)	3.001 (2.121)	2.812 (2.015)	2.605 (1.869)	2.295 (1.587)	2.859 (2.044)
**Sex of child**						
Male	76,801 (52.0%)	72,932 (52.3%)	66,873 (52.2%)	62,352 (52.3%)	54,737 (52.7%)	333,695 (52.3%)
Female	70,865 (48.0%)	66,436 (47.7%)	61,188 (47.8%)	56,902 (47.7%)	49,179 (47.3%)	304,570 (47.7%)
**BMI**						
Underweight	27,444 (18.6%)	20,444 (14.7%)	15,566 (12.2%)	11,281 (9.5%)	6,228 (6.0%)	80,963 (12.7%)
Normal	94,205 (63.8%)	87,527 (62.8%)	76,398 (59.7%)	66,629 (55.9%)	50,182 (48.3%)	374,941 (58.7%)
Overweight	14,000 (9.5%)	17,724 (12.7%)	20,846 (16.3%)	24,425 (20.5%)	26,512 (25.5%)	103,507 (16.2%)
Obese	12,017 (8.1%)	13,673 (9.8%)	15,251 (11.9%)	16,919 (14.2%)	20,994 (20.2%)	78,854 (12.4%)

BMI, body mass index.

The mean facility birth rate was 70% (IQR: 56%, 92%), mean DPT3 vaccination coverage 76% (IQR: 66%, 90%), mean measles vaccination coverage 77% (IQR: 70%, 89%), mean PNC utilization rate 50% (IQR: 32%, 69%), average stunting rate 29% (IQR: 20%, 38%), and average wasting rate was 9% (IQR: 5%, 12%). In general, the richest and richer quintiles had higher baseline facility birth and vaccination rates and lower stunting and wasting rates compared to the poorer and poorest quintiles. Detailed baseline rates across countries and wealth quintiles are presented in S3–S8 Tables.

**Fig 1:**
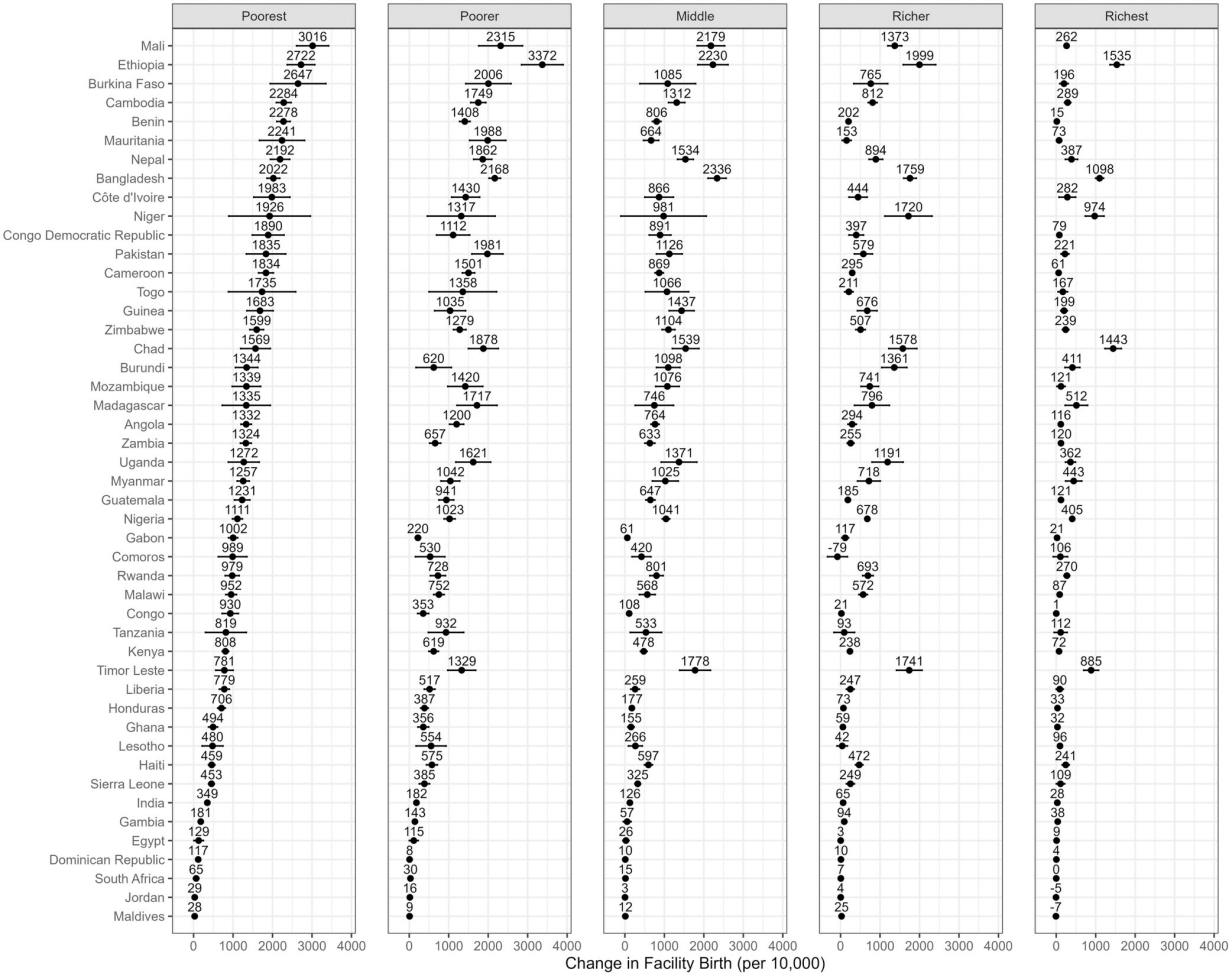
Facility birth rate change associated with achieving recommended ANC visits and quality across 5 wealth quintiles and countries. This change is measured by the predicted difference in facility birth rate between 2 scenarios: an intervention that ensures all women achieve the adequate level of ANC utilization and quality, and a baseline scenario that represents the current state of ANC in each country. The dots represent point estimates of the changes, and the lines around the dots denote the 95% confidence intervals. ANC, antenatal care.

**Fig 2:**
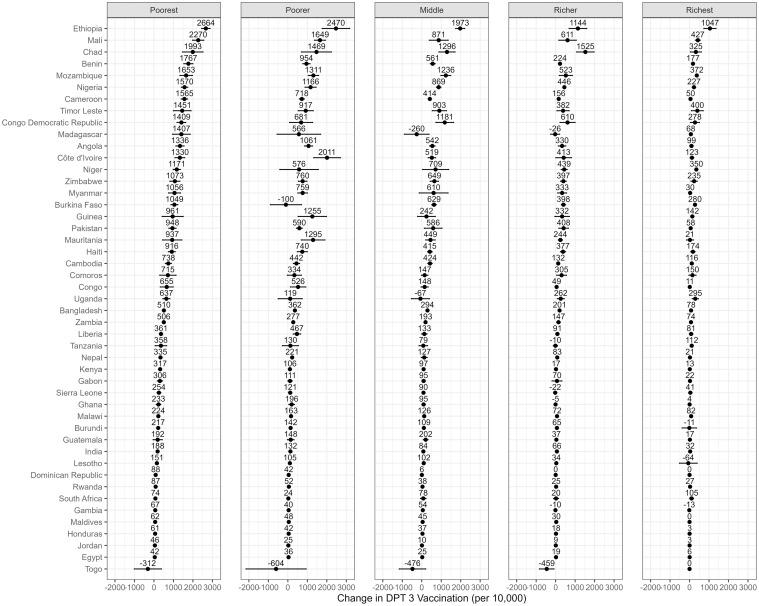
DPT3 vaccination rate change associated with achieving recommended ANC visits and quality across 5 wealth quintiles and countries. This change is measured by the predicted difference in DPT3 vaccination rate between 2 scenarios: an intervention that ensures all women achieve the adequate level of ANC utilization and quality, and a baseline scenario that represents the current state of ANC in each country. The dots represent point estimates of the changes, and the lines around the dots denote the 95% confidence intervals. ANC, antenatal care; DPT3, diphtheria-pertussis-tetanus third dose.

**Fig 3:**
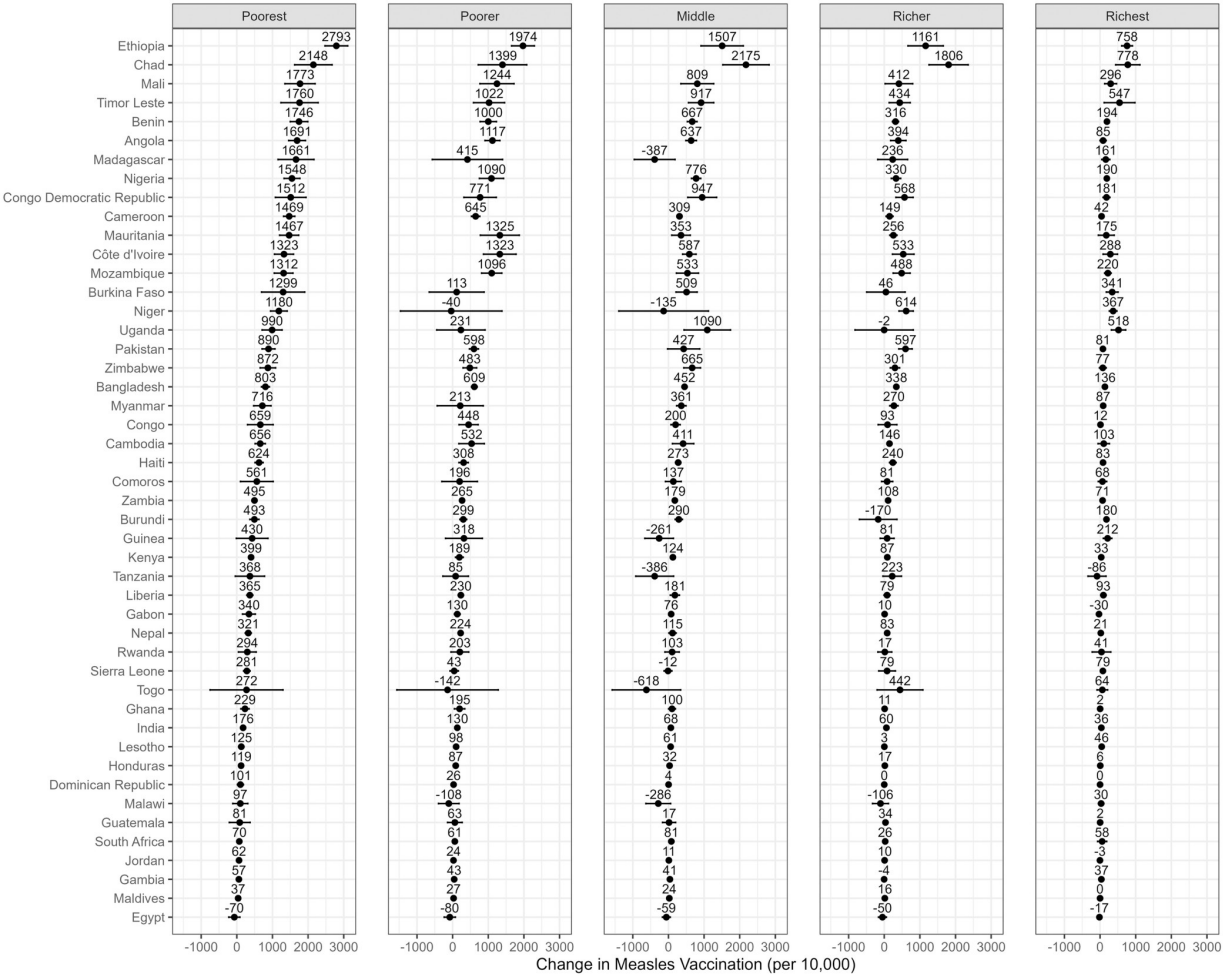
Measles vaccination rate change associated with achieving recommended ANC visits and quality across 5 wealth quintiles and countries. This change is measured by the predicted difference in measles vaccination rate between 2 scenarios: an intervention that ensures all women achieve the adequate level of ANC utilization and quality, and a baseline scenario that represents the current state of ANC in each country. The dots represent point estimates of the changes, and the lines around the dots denote the 95% confidence intervals. ANC, antenatal care.

**Fig 4:**
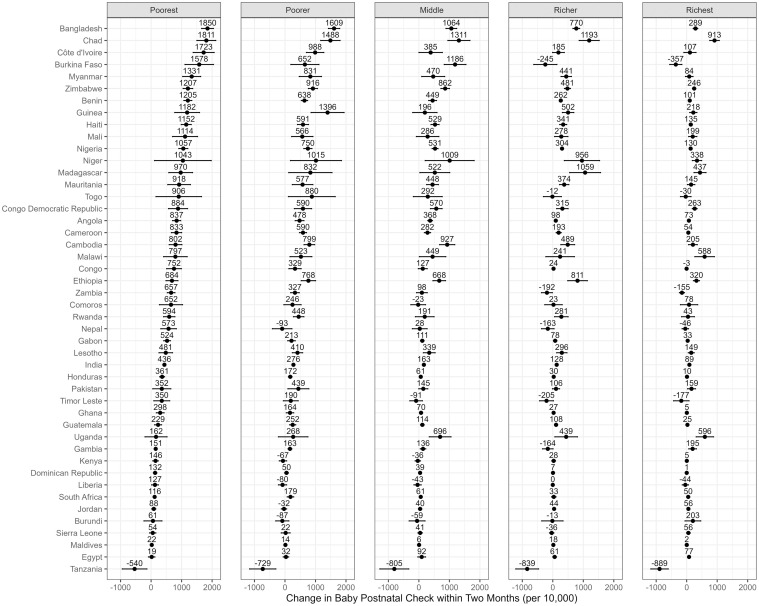
PNC utilization rate change associated with achieving recommended ANC visits and quality across 5 wealth quintiles and countries. This change is measured by the predicted difference in PNC utilization rate between 2 scenarios: an intervention that ensures all women achieve the adequate level of ANC utilization and quality, and a baseline scenario that represents the current state of ANC in each country. The dots represent point estimates of the changes, and the lines around the dots denote the 95% confidence intervals. ANC, antenatal care; PNC, postnatal care.

Figs 1–4 depict the predicted differences in facility birth, DPT3 vaccination, measles vaccination, and PNC utilization between the 2 scenarios: the intervention scenario, which involves achieving adequate ANC visits and quality, and the baseline scenario. (Detailed data can be found in S9–S12 Tables.) The differences were generally positive for lower wealth quintiles yet varied across countries.

Achieving adequate ANC visits and quality was statistically significantly associated with an increase in facility birth in 46 countries in the poorest quintile, 45 in the poorer quintile, 40 in the middle quintile, 40 in the richer quintile, and 37 in the richest quintile, compared to baseline levels. The Maldives demonstrated the lowest estimate within the poorest quintile, noting an increase of 28 per 10,000 in the facility birth rate. Conversely, Mali experienced the most substantial increase in this quintile with a rise of 3,016 per 10,000. The richest quintile witnessed a more modest change, ranging from a decrease of 7 per 10,000 in the Maldives to an increase of 1,535 per 10,000 in Ethiopia.

Moreover, statistically significant positive differences in DPT3 vaccination coverage between the 2 scenarios were observed across 43 countries in the poorest quintile, 37 in the poorer quintile, 35 in the middle quintile, 33 in the richer quintile, and 29 in the richest quintile. The extent of these differences varied from a decrease of 604 per 10,000 in Togo (poorest quintile) to an increase of 2,664 per 10,000 in Ethiopia (poorest quintile). Achieving adequate ANC was also associated with positive shifts in measles vaccination coverage, with some exceptions. Statistically significant values were observed in 40 countries in the poorest quintile, 30 in the poorer quintile, 32 in the middle quintile, 23 in the richer quintile, and 27 in the richest quintile. This change spanned from a decrease of 618 per 10,000 in Togo (middle quintile) to an increase of 2,793 per 10,000 in Ethiopia (poorest quintile).

Additionally, achieving statistically sufficient ANC was significantly associated with an increase in postnatal check rate for newborns in 40 countries in the poorest quintile, 33 in the poorer quintile, 28 in the middle quintile, 26 in the richer quintile, and 26 in the richest quintile. The potential change ranged from a decrease of 889 per 10,000 in Tanzania (richest quintile) to an increase of 1,850 per 10,000 in Bangladesh (poorest quintile).

The results related to stunting and wasting are provided in S2 and S3 Figs, as well as in S13 and S14 Tables. In general, the estimated associations were consistent across countries and the magnitude was smaller when compared to the previously mentioned indicators. Achieving adequate ANC was statistically significantly associated with a decrease in the stunting rate in 35 countries in the poorest quintile, 22 in the poorer quintile, 23 in the middle quintile, 21 in the richer quintile, and 24 in the richest quintile. Similarly, achieving adequate ANC was statistically significantly associated with a reduction in the wasting rate in 12 countries in the poorest quintile, 11 in the poorer quintile, 12 in the middle quintile, 15 in the richer quintile, and 11 in the richest quintile.

## Discussion

The analysis, conducted in 47 LMICs, indicates a potential association between adequate utilization and quality of ANC with improved health-seeking behaviors. Overall, meeting the ANC guidelines of at least 4 high-quality visits would be associated with increased facility birth, higher rates of PNC checks, and improved DPT3 and measles immunization coverage. Notably, the association would be more pronounced in lower wealth quintiles, highlighting the potentially greater health impact of ANC for disadvantaged populations.

A few exceptions are worth noting. For example, achieving adequate ANC utilization and quality displayed a negative association with PNC utilization in Tanzania. Prior studies have demonstrated that mothers in Tanzania giving birth in health facilities are less inclined to seek PNC compared to those giving birth outside such facilities [29,30]. One potential explanation is that women who give birth in such facilities might not receive appropriate guidance on the importance of returning for postnatal check-ups [29,30]. Conversely, those giving birth at home may feel a greater imperative to have both themselves and their newborns checked, or to officially register the births [29,30]. Thus, this negative association could potentially be attributed to the increased facility birth resulting from improved ANC.

To our knowledge, this is the first comprehensive study to assess association of achieving both adequate ANC utilization and quality with a range of health indicators, while considering a large sample of countries and accounting for socioeconomic status. Our findings show that the strength of the association would differ across countries and wealth quintiles. One possible explanation is that baseline ANC utilization and quality vary substantially across countries, as documented previously [16]. For example, since Ethiopia exhibits a lower baseline, achieving adequate level of ANC may lead to a substantial shift. In contrast, Ghana, with its initially higher baseline, would experience a more modest increase from achieving the same adequate level of ANC, potentially leading to a less pronounced impact on health-seeking behaviors. Additionally, individuals in lower wealth quintiles generally display lower ANC utilization and quality levels.

Another contributing factor to the heterogeneity in the estimated associations could be the variation in baseline health indicators. For example, Ethiopia’s baseline facility birth rate is lower than that of Malawi, suggesting a greater potential for improvement [31]. Thus, attaining an adequate level of ANC could have a greater impact on the facility birth rate in Ethiopia than in Malawi. This variation in baseline rate across countries can be attributed to several factors, such as socioeconomic status, the number of available birth facilities, the proximity to the nearest facility and the state of transportation infrastructure [32–34]. Furthermore, individuals in lower quintiles tend to have lower rates of facility birth overall. Consequently, our study suggests that strengthening ANC has the potential to benefit countries with lower utilization rates of these types of health care, thus contributing to global equity.

Recent challenges, notably a decline in immunization coverage and the redirection of funds toward addressing the COVID-19 pandemic, have posed significant challenges to maintaining adequate immunization coverage [35]. In this context, our findings provide compelling evidence of a positive association between achieving adequate ANC utilization and quality and childhood immunization coverage, particularly for DPT3 and measles immunizations. Therefore, strengthening ANC might serve as a promising strategy to raise immunization coverage.

The significance of our study lies in its contribution to the existing body of literature, which has predominantly focused on the direct impact of ANC on short-term mortality outcomes like neonatal and infant mortality [36–38]. Our findings, however, extend beyond this narrower scope by examining what might be the potential broader impact of ANC on subsequent health behaviors, ultimately leading to positive effects on both mother and child health. Solely emphasizing the direct mortality impact of ANC may underestimate its true value given that ANC plays a pivotal role in shaping overall health behaviors and optimizing the well-being of mothers and children.

Despite the strengths of our study, several limitations should be acknowledged. First, despite rigorous adjustments for potential confounders, it is possible that unobserved variables may still impact outcomes. These estimated associations could reflect the overall quality of health systems that offer both high-quality ANC and strong encouragement or provision of other care components (e.g., free ANC and PNC within the public health system), rather than ANC directly leading to these outcomes. We applied LASSO to identify relevant interaction terms and squared terms for ANC visits. While LASSO is a robust method commonly used in research, it has its limitations. For example, the number of predictors it selects is limited by the sample size, and it tends to choose only 1 or a few predictors from a group of correlated variables [39]. Second, our assessment predominantly concentrated on the technical dimensions of ANC quality, potentially neglecting women’s perceptions of quality. Notably, technical and perceived quality do not always correlate positively, underscoring the potential inadequacy of solely relying on the technical dimension to encapsulate the entirety of quality health care [40]. Third, our analysis omitted a range of conceivable indicators, including maternal mortality, risk behaviors (e.g., smoking and alcohol use), and comorbidities. The role of maternal health is particularly important in this context; ANC has the potential to enhance maternal well-being, which in turn can have a direct positive impact on child health. Investigating these relationships could further substantiate the critical importance of ANC, providing additional evidence to support the findings of this study. Fourth, using cross-sectional self-reported data from DHS introduces the potential for recall and response biases. A longitudinal approach, such as a trial following women and children over time, would provide a more comprehensive understanding. Fifth, the DHS do not detail the exact timing of each ANC visit. Fifth, our analysis excluded individuals with missing data, a majority of whom lacked information on ANC visits or quality. These individuals typically have lower socioeconomic status and poorer health compared to the included sample. Consequently, given the patterns observed in our study, there is a possibility that the impact of adequate ANC might have been underestimated. Sixth, our definition of adequate ANC utilization as at least 4 visits aligns with previous WHO guidelines. We recommend that future research should replicate our analysis using the more recent recommendation of at least 8 visits, once sufficient post-2016 data becomes available. Finally, the DHS wealth quintiles are relative to a country’s population and context and, therefore, are not directly comparable across countries. These acknowledged limitations offer directions for future research refinement and investigation.

In conclusion, our study demonstrates that achieving adequate levels of ANC utilization and quality could be associated with important gains in health care use for most LMICs, including facility birth, PNC checks, and childhood immunization coverage. This potential impact would likely be substantial for individuals in lower wealth quintiles and among countries with lower baseline levels of ANC and other health care. Strengthening ANC could yield major indirect health benefits beyond the proven short-term gains in reducing morbidity and mortality and, therefore, contribute to addressing global health inequities. These insights could encourage the adoption of the new ANC guidelines, which has been delayed and poor across many countries.

## Supporting information

S1 Table Countries included and corresponding Demographic and Health Surveys (DHS), along with gross national income (GNI) estimates. (DOCX)

S2 Table Distribution of demographic and health variables (number and proportion of participants) for the sample with missing values on antenatal care utilization or quality (not included in the analysis). (DOCX)

S3 Table Baseline unweighted absolute facility birth rates (per 10,000) across wealth quintiles and countries. (DOCX)

S4 Table Baseline unweighted absolute diphtheria-pertussis-tetanus third dose (DPT3) vaccination rate (per 10,000) across wealth quintiles and countries. (DOCX)

S5 Table Baseline unweighted absolute measles vaccination rates (per 10,000) across wealth quintiles and countries. (DOCX)

S6 Table Baseline unweighted absolute postnatal care utilization rates (per 10,000) across wealth quintiles and countries. (DOCX)

S7 Table Baseline unweighted absolute stunting rates (per 10,000) across wealth quintiles and countries. (DOCX)

S8 Table Baseline unweighted absolute wasting rates (per 10,000) across wealth quintiles and countries. (DOCX)

S9 Table Facility birth rate change (per 10,000) (with 95% confidence interval and *p*-value) associated with achieving recommended antenatal care visits and quality. (DOCX)

S10 Table Diphtheria-pertussis-tetanus third dose (DPT3) vaccination rate change (per 10,000) (with 95% confidence interval and *p*-value) associated with achieving recommended antenatal care visits and quality. (DOCX)

S11 Table Measles vaccination rate change (per 10,000) (with 95% confidence interval and *p*-value) associated with achieving recommended antenatal care visits and quality. (DOCX)

S12 Table Postnatal care utilization rate change (per 10,000) (with 95% confidence interval and *p*-value) associated with achieving recommended antenatal care visits and quality. (DOCX)

S13 Table Stunting rate change (per 10,000) (with 95% confidence interval and *p*-value) associated with recommended antenatal care visits and quality. (DOCX)

S14 Table Wasting rate change (per 10,000) (with 95% confidence interval) and *p*-value associated with recommended antenatal care visits and quality. (DOCX)

S1 Fig The statistical analysis process. (DOCX)

S2 Fig Stunting rate change associated with recommended antenatal care visits and quality across 5 wealth quintiles and countries. (DOCX)

S3 Fig Wasting rate change associated with recommended antenatal care visits and quality across 5 wealth quintiles and countries. (DOCX)

S1 STROBE Checklist STROBE Statement—Checklist of items that should be included in reports of observational studies. (DOCX)

S1 File Inclusivity in global research questionnaire. (DOCX)

## Abbreviations

ANC: antenatal care
DHS: Demographic and Health Surveys
DPT3: diphtheria-pertussis-tetanus third dose
IQR: interquartile range
LASSO: least absolute shrinkage and selection operator
LMIC: low- and middle-income country
PNC: postnatal care
SD: standard deviation
WHO: World Health Organization
