# Supplementary material for: Association between achieving adequate antenatal care and health-seeking behaviors: A study of Demographic and Health Surveys in 47 low- and middle-income countries
Source: PLoS Med. 2024 Jul 5;21(7):e1004421. doi: 10.1371/journal.pmed.1004421 (PMC11226092; doi:10.1371/journal.pmed.1004421)
Supplement: S1 Table — (DOCX) [file pmed.1004421.s001.docx]

**S1 Table.** Countries included and corresponding Demographic and Health Surveys (DHS), along with gross national income (GNI) estimates.

| **Country** | **Standard DHS survey** | **GNI (2022 USD)*** |
| --- | --- | --- |
| Angola | 2015–2016 | 1,900 |
| Bangladesh | 2011; 2014; 2017–2018 | 2,820 |
| Benin | 2011–2012; 2017–2018 | 1,400 |
| Burkina Faso | 2010 | 840 |
| Burundi | 2010; 2016–2017 | 240 |
| Cambodia | 2010; 2014; 2021–2022 | 1,700 |
| Cameroon | 2011; 2018 | 1,660 |
| Chad | 2014–2015 | 690 |
| Comoros | 2012 | 1,610 |
| Congo | 2011–2012 | 2,060 |
| Congo, Democratic Republic of | 2013–2014; 2017 | 590 |
| Côte d'Ivoire | 2011–2012 | 2,620 |
| Dominican Republic | 2013 | 9,050 |
| Egypt | 2014 | 4,100 |
| Ethiopia | 2011; 2016; 2019 (interim) | 1,020 |
| Gabon | 2012; 2019–2021 | 7,540 |
| Gambia | 2013; 2019–2020 | 810 |
| Ghana | 2014 | 2,350 |
| Guatemala | 2014–2015 | 5,350 |
| Guinea | 2012; 2018 | 1,180 |
| Haiti | 2012; 2016–2017 | 1,610 |
| Honduras | 2011–2012 | 2,740 |
| India | 2015–2016; 2019–2021 | 2,380 |
| Jordan | 2012; 2017–2018 | 4,260 |
| Kenya | 2014; 2022 | 2,170 |
| Lesotho | 2014 | 1,260 |
| Liberia | 2013; 2019–2020 | 680 |
| Madagascar | 2021 | 510 |
| Malawi | 2010; 2015–2016 | 640 |
| Maldives | 2016–2017 | 11,030 |
| Mali | 2012–2013; 2018 | 850 |
| Mauritania | 2019–2021 | 2,160 |
| Mozambique | 2011 | 500 |
| Myanmar | 2015–2016 | 1,210 |
| Nepal | 2011; 2016; 2022 | 1,340 |
| Niger | 2012 | 610 |
| Nigeria | 2013; 2018 | 2,140 |
| Pakistan | 2012–2013; 2017–2018 | 1,580 |
| Rwanda | 2010; 2014–2015; 2019–2020 | 930 |
| Sierra Leone | 2013; 2019 | 510 |
| South Africa | 2016 | 6,780 |
| Tanzania | 2010; 2015–2016 | 1,200 |
| Timor-Leste | 2009–2010; 2016 | 1,970 |
| Togo | 2013–2014 | 990 |
| Uganda | 2011; 2016 | 930 |
| Zambia | 2013–2014; 2018 | 1,170 |
| Zimbabwe | 2010–2011; 2015 | 1,500 |

*Source: The World Bank.
