# Supplementary material for: Association between achieving adequate antenatal care and health-seeking behaviors: A study of Demographic and Health Surveys in 47 low- and middle-income countries
Source: PLoS Med. 2024 Jul 5;21(7):e1004421. doi: 10.1371/journal.pmed.1004421 (PMC11226092; doi:10.1371/journal.pmed.1004421)
Supplement: S2 Table — (DOCX) [file pmed.1004421.s002.docx]

**S2 Table**. Distribution of demographic and health variables (presented as n or mean (%), number or mean and proportion of participants) for the sample with missing values on antenatal care utilization or quality (not included in the analysis).

|  | **Poorest** | **Poorer** | **Middle** | **Richer** | **Richest** | **Total** |
| --- | --- | --- | --- | --- | --- | --- |
| **Age at childbirth** |  |  |  |  |  |  |
| <25 years | 1,263 (20.3%) | 1,078 (21.1%) | 740 (20.7%) | 601 (19.4%) | 359 (14.6%) | 4,041 (19.7%) |
| 25–34 years | 3,206 (51.4%) | 2,820 (55.2%) | 2,071 (57.8%) | 1,860 (60.1%) | 1,562 (63.6%) | 11,519 (56.3%) |
| >34 years | 1,767 (28.3%) | 1,209 (23.7%) | 772 (21.5%) | 635 (20.5%) | 534 (21.8%) | 4,917 (24.0%) |
| **Education** |  |  |  |  |  |  |
| No education | 12,664 (85.5%) | 8,689 (73.5%) | 5,363 (58.1%) | 3,011 (41.3%) | 1,116 (19.4%) | 30,843 (63.1%) |
| Primacy | 1,481 (10.0%) | 1,804 (15.3%) | 1,731 (18.8%) | 1,555 (21.3%) | 734 (12.7%) | 7,305 (14.9%) |
| Secondary | 568 (3.8%) | 1,063 (9.0%) | 1,588 (17.2%) | 1,851 (25.4%) | 2,002 (34.7%) | 7,072 (14.5%) |
| Higher | 94 (0.6%) | 263 (2.2%) | 545 (5.9%) | 867 (11.9%) | 1,915 (33.2%) | 3,684 (7.5%) |
| **Marital status** |  |  |  |  |  |  |
| Never in union | 9 (0.1%) | 10 (0.1%) | 7 (0.1%) | 5 (0.1%) | 0 (0.0%) | 31 (0.1%) |
| Married/ living with partner | 14,426 (97.4%) | 11,501 (97.3%) | 8,955 (97.1%) | 7,119 (97.7%) | 5,659 (98.1%) | 47,660 (97.5%) |
| Windowed/divorced/separated | 372 (2.5%) | 308 (2.6%) | 265 (2.9%) | 160 (2.2%) | 108 (1.9%) | 1,213 (2.5%) |
| **Place of residence** |  |  |  |  |  |  |
| Urban | 2,226 (15.0%) | 3,615 (30.6%) | 4,688 (50.8%) | 5,011 (68.8%) | 4,700 (81.5%) | 20,240 (41.4%) |
| Rural | 12,581 (85.0%) | 8,204 (69.4%) | 4,539 (49.2%) | 2,273 (31.2%) | 1,067 (18.5%) | 28,664 (58.6%) |
| **Literacy** |  |  |  |  |  |  |
| Cannot real at all | 12,805 (86.5%) | 8,862 (75.0%) | 5,371 (58.3%) | 3,084 (42.4%) | 1,126 (19.5%) | 31,248 (63.9%) |
| Able to read | 1,912 (12.9%) | 2,940 (24.9%) | 3,823 (41.5%) | 4,187 (57.5%) | 4,638 (80.5%) | 17,500 (35.8%) |
| Not ascertained | 81 (0.5%) | 8 (0.1%) | 25 (0.3%) | 9 (0.1%) | 0 (0.0%) | 123 (0.3%) |
| **Birth order** | 3.300 (2.238) | 3.062 (2.132) | 2.829 (1.999) | 2.578 (1.823) | 2.212 (1.547) | 2.918 (2.067) |
| **Sex of child** |  |  |  |  |  |  |
| Male | 7,512 (50.7%) | 6,012 (50.9%) | 4,656 (50.5%) | 3,640 (50.0%) | 2,778 (48.2%) | 24,598 (50.3%) |
| Female | 7,295 (49.3%) | 5,807 (49.1%) | 4,571 (49.5%) | 3,644 (50.0%) | 2,989 (51.8%) | 24,306 (49.7%) |
| **BMI** |  |  |  |  |  |  |
| Underweight | 688 (4.6%) | 361 (3.1%) | 243 (2.6%) | 134 (1.8%) | 55 (1.0%) | 1,481 (3.0%) |
| Normal | 3,406 (23.0%) | 2,507 (21.2%) | 1,449 (15.7%) | 1,176 (16.1%) | 761 (13.2%) | 9,299 (19.0%) |
| Overweight | 1,424 (9.6%) | 1,403 (11.9%) | 1,140 (12.4%) | 1,016 (13.9%) | 828 (14.4%) | 5,811 (11.9%) |
| Obese | 9,289 (62.7%) | 7,548 (63.9%) | 6,395 (69.3%) | 4,958 (68.1%) | 4,123 (71.5%) | 32,313 (66.1%) |
