# Supplementary material for: Association between achieving adequate antenatal care and health-seeking behaviors: A study of Demographic and Health Surveys in 47 low- and middle-income countries
Source: PLoS Med. 2024 Jul 5;21(7):e1004421. doi: 10.1371/journal.pmed.1004421 (PMC11226092; doi:10.1371/journal.pmed.1004421)
Supplement: S3 Table — (DOCX) [file pmed.1004421.s003.docx]

**S3 Table**. Baseline unweighted absolute facility birth rates (per 10,000) across wealth quintiles and countries.

| **Country** | **Poorest** | **Poorer** | **Middle** | **Richer** | **Richest** |
| --- | --- | --- | --- | --- | --- |
| Angola | 1380 | 2929 | 5863 | 7634 | 8733 |
| Bangladesh | 1574 | 2435 | 3405 | 4707 | 7173 |
| Benin | 6846 | 8207 | 8794 | 9649 | 9918 |
| Burkina Faso | 5289 | 6329 | 7466 | 8292 | 9622 |
| Burundi | 6906 | 7386 | 7704 | 7717 | 9171 |
| Cambodia | 6276 | 7005 | 7719 | 8503 | 9254 |
| Cameroon | 2439 | 5675 | 7543 | 9024 | 9633 |
| Chad | 1231 | 1419 | 1185 | 1315 | 5603 |
| Comoros | 5931 | 7302 | 8521 | 8689 | 9114 |
| Congo | 7574 | 9013 | 9549 | 9808 | 9815 |
| Congo, Democratic Republic of | 5780 | 6599 | 7663 | 8925 | 9856 |
| Côte d'Ivoire | 3107 | 4126 | 6182 | 8035 | 9191 |
| Dominican Republic | 9379 | 9941 | 9971 | 9964 | 9955 |
| Egypt | 8185 | 8412 | 8986 | 9410 | 9816 |
| Ethiopia | 921 | 1605 | 1647 | 2154 | 6940 |
| Gabon | 7517 | 9391 | 9543 | 9612 | 9783 |
| Gambia | 6238 | 6372 | 6979 | 8391 | 9627 |
| Ghana | 4985 | 6083 | 7915 | 9437 | 9652 |
| Guatemala | 4190 | 5960 | 7540 | 9039 | 9498 |
| Guinea | 1862 | 3359 | 4257 | 6254 | 8420 |
| Haiti | 1281 | 2365 | 4240 | 5551 | 7907 |
| Honduras | 5944 | 8099 | 9083 | 9607 | 9801 |
| India | 8007 | 8901 | 9358 | 9611 | 9788 |
| Jordan | 9788 | 9926 | 9973 | 9977 | 9668 |
| Kenya | 4016 | 6804 | 7783 | 8786 | 9464 |
| Lesotho | 6277 | 7388 | 8321 | 8983 | 9401 |
| Liberia | 5509 | 6664 | 7200 | 7712 | 8533 |
| Madagascar | 1779 | 2962 | 3901 | 5006 | 7370 |
| Malawi | 7852 | 8161 | 8343 | 8875 | 9513 |
| Maldives | 9648 | 9609 | 9314 | 9714 | 9126 |
| Mali | 3482 | 4577 | 5438 | 7710 | 9470 |
| Mauritania | 4183 | 5895 | 8421 | 9470 | 9841 |
| Mozambique | 3790 | 4574 | 5952 | 7765 | 9318 |
| Myanmar | 1781 | 2553 | 3800 | 5259 | 8038 |
| Nepal | 4019 | 4941 | 6138 | 7232 | 8850 |
| Niger | 2067 | 2650 | 2832 | 4322 | 8195 |
| Nigeria | 868 | 2162 | 4131 | 5969 | 8132 |
| Pakistan | 5364 | 6145 | 7014 | 7866 | 9120 |
| Rwanda | 8032 | 8482 | 8666 | 8869 | 9494 |
| Sierra Leone | 6489 | 6875 | 6788 | 7423 | 8112 |
| South Africa | 9080 | 9559 | 9760 | 9834 | 10000 |
| Tanzania | 4069 | 4820 | 5890 | 7306 | 9000 |
| Timor Leste | 1226 | 1933 | 2972 | 4740 | 7172 |
| Togo | 4736 | 6302 | 7780 | 9420 | 9750 |
| Uganda | 5578 | 6266 | 6791 | 7458 | 9102 |
| Zambia | 6426 | 7231 | 7712 | 8826 | 9654 |
| Zimbabwe | 5597 | 6465 | 7123 | 8636 | 9344 |
