# Supplementary material for: Association between achieving adequate antenatal care and health-seeking behaviors: A study of Demographic and Health Surveys in 47 low- and middle-income countries
Source: PLoS Med. 2024 Jul 5;21(7):e1004421. doi: 10.1371/journal.pmed.1004421 (PMC11226092; doi:10.1371/journal.pmed.1004421)
Supplement: S4 Table — (DOCX) [file pmed.1004421.s004.docx]

**S4 Table.** Baseline unweighted absolute diphtheria-pertussis-tetanus 3rd dose (DPT3) vaccination rate (per 10,000) across wealth quintiles and countries.

| **Country** | **Poorest** | **Poorer** | **Middle** | **Richer** | **Richest** |
| --- | --- | --- | --- | --- | --- |
| Angola | 2076 | 2714 | 4311 | 6087 | 6106 |
| Bangladesh | 8854 | 9271 | 9248 | 9461 | 9644 |
| Benin | 5377 | 6340 | 6764 | 7308 | 7997 |
| Burkina Faso | 8256 | 9028 | 9184 | 9228 | 9573 |
| Burundi | 9595 | 9603 | 9639 | 9665 | 9464 |
| Cambodia | 7111 | 8105 | 8136 | 9126 | 9223 |
| Cameroon | 5014 | 6747 | 7548 | 7846 | 8734 |
| Chad | 2631 | 2872 | 2553 | 3034 | 4155 |
| Comoros | 6102 | 6700 | 8182 | 8642 | 8125 |
| Congo | 4145 | 5885 | 5254 | 6737 | 6053 |
| Congo, Democratic Republic of | 3826 | 4394 | 5057 | 6217 | 8165 |
| Côte d'Ivoire | 4462 | 5902 | 7109 | 7812 | 9036 |
| Dominican Republic | 8927 | 9390 | 8981 | 9351 | 9710 |
| Egypt | 9587 | 9730 | 9676 | 9746 | 9849 |
| Ethiopia | 2782 | 4769 | 5109 | 5570 | 7601 |
| Gabon | 3499 | 4952 | 5136 | 5806 | 5672 |
| Gambia | 9129 | 9032 | 9180 | 8710 | 8917 |
| Ghana | 8624 | 8852 | 8764 | 8690 | 9351 |
| Guatemala | 8078 | 8440 | 8773 | 9148 | 8986 |
| Guinea | 2848 | 4207 | 3860 | 4510 | 5860 |
| Haiti | 5177 | 5797 | 6122 | 6434 | 7239 |
| Honduras | 9615 | 9673 | 9454 | 9668 | 9829 |
| India | 8504 | 8753 | 8985 | 9037 | 9064 |
| Jordan | 8967 | 8990 | 9171 | 9176 | 8750 |
| Kenya | 8345 | 9273 | 9091 | 9062 | 9376 |
| Lesotho | 8767 | 8871 | 9344 | 8704 | 8182 |
| Liberia | 5664 | 7259 | 7692 | 7516 | 7500 |
| Madagascar | 5296 | 6316 | 7857 | 7556 | 8750 |
| Malawi | 9395 | 9353 | 9455 | 9525 | 9578 |
| Maldives | 8504 | 8472 | 8069 | 8600 | 8500 |
| Mali | 5294 | 6195 | 6236 | 6831 | 7682 |
| Mauritania | 7254 | 6995 | 8361 | 7933 | 8312 |
| Mozambique | 6577 | 7106 | 7948 | 8626 | 8998 |
| Myanmar | 5100 | 5699 | 6933 | 7688 | 8696 |
| Nepal | 8992 | 9070 | 9041 | 9361 | 9474 |
| Niger | 6604 | 6905 | 7935 | 7861 | 8955 |
| Nigeria | 1466 | 2450 | 4778 | 6459 | 8040 |
| Pakistan | 4810 | 7343 | 7377 | 7752 | 8976 |
| Rwanda | 9674 | 9792 | 9868 | 9905 | 9852 |
| Sierra Leone | 7742 | 8094 | 8244 | 8140 | 7814 |
| South Africa | 7848 | 7246 | 7015 | 7255 | 5652 |
| Tanzania | 8163 | 8779 | 8938 | 9364 | 9521 |
| Timor Leste | 5382 | 6155 | 6639 | 7299 | 7430 |
| Togo | 9194 | 8493 | 8370 | 8729 | 9216 |
| Uganda | 7925 | 7391 | 7801 | 7556 | 8093 |
| Zambia | 8341 | 8762 | 8966 | 9400 | 9527 |
| Zimbabwe | 7568 | 7852 | 8234 | 8357 | 8420 |
