# Supplementary material for: Association between achieving adequate antenatal care and health-seeking behaviors: A study of Demographic and Health Surveys in 47 low- and middle-income countries
Source: PLoS Med. 2024 Jul 5;21(7):e1004421. doi: 10.1371/journal.pmed.1004421 (PMC11226092; doi:10.1371/journal.pmed.1004421)
Supplement: S5 Table — (DOCX) [file pmed.1004421.s005.docx]

**S5 Table.** Baseline unweighted absolute measles vaccination rates (per 10,000) across wealth quintiles and countries.

| **Country** | **Poorest** | **Poorer** | **Middle** | **Richer** | **Richest** |
| --- | --- | --- | --- | --- | --- |
| Angola | 3090 | 4126 | 6099 | 7189 | 8407 |
| Bangladesh | 8020 | 8577 | 8669 | 8830 | 9241 |
| Benin | 5456 | 6523 | 7325 | 7715 | 8591 |
| Burkina Faso | 7791 | 8854 | 9085 | 9018 | 9188 |
| Burundi | 9063 | 9428 | 9381 | 9381 | 9542 |
| Cambodia | 6717 | 7697 | 7818 | 8857 | 9128 |
| Cameroon | 5201 | 6495 | 7228 | 7742 | 8610 |
| Chad | 5200 | 5230 | 5035 | 4907 | 6604 |
| Comoros | 6870 | 6400 | 8000 | 8519 | 8438 |
| Congo | 6119 | 7115 | 7458 | 8421 | 8684 |
| Congo, Democratic Republic of | 5516 | 6247 | 6752 | 6997 | 8670 |
| Côte d'Ivoire | 4703 | 5912 | 6484 | 7209 | 8675 |
| Dominican Republic | 9018 | 9236 | 9151 | 9559 | 9500 |
| Egypt | 3463 | 3633 | 3849 | 4102 | 4264 |
| Ethiopia | 4166 | 5762 | 6066 | 6311 | 8127 |
| Gabon | 6053 | 7152 | 7123 | 8141 | 7368 |
| Gambia | 9279 | 9220 | 9309 | 9128 | 8535 |
| Ghana | 8519 | 8852 | 8764 | 8571 | 9351 |
| Guatemala | 6037 | 5996 | 6369 | 7018 | 7029 |
| Guinea | 3793 | 4466 | 4416 | 4921 | 7222 |
| Haiti | 6040 | 6058 | 6769 | 6875 | 7125 |
| Honduras | 9047 | 9006 | 8725 | 8907 | 9106 |
| India | 8426 | 8675 | 8894 | 8993 | 9080 |
| Jordan | 7988 | 7961 | 7790 | 8471 | 7750 |
| Kenya | 7653 | 8881 | 8885 | 9163 | 9376 |
| Lesotho | 8784 | 8710 | 9677 | 9259 | 8837 |
| Liberia | 6135 | 7440 | 7393 | 7255 | 8571 |
| Madagascar | 4721 | 5356 | 7188 | 7333 | 8478 |
| Malawi | 9337 | 9217 | 9111 | 9430 | 9577 |
| Maldives | 9206 | 8819 | 8414 | 8800 | 9000 |
| Mali | 6118 | 6727 | 6602 | 7280 | 7787 |
| Mauritania | 6649 | 7192 | 8667 | 8652 | 8438 |
| Mozambique | 7520 | 7454 | 8208 | 9054 | 9406 |
| Myanmar | 7510 | 6891 | 8267 | 8608 | 9304 |
| Nepal | 9018 | 8937 | 9074 | 9323 | 9408 |
| Niger | 6226 | 7063 | 7308 | 7861 | 8172 |
| Nigeria | 1870 | 3187 | 5065 | 6302 | 8060 |
| Pakistan | 4650 | 7019 | 7220 | 7397 | 8498 |
| Rwanda | 6454 | 7260 | 7252 | 7424 | 7135 |
| Sierra Leone | 7931 | 7545 | 7677 | 7664 | 8433 |
| South Africa | 8462 | 8841 | 9385 | 8800 | 8696 |
| Tanzania | 7828 | 8150 | 8415 | 9104 | 9227 |
| Timor Leste | 5582 | 6462 | 7355 | 7892 | 7991 |
| Togo | 8280 | 7397 | 7407 | 8220 | 8431 |
| Uganda | 7736 | 7492 | 7875 | 7788 | 8494 |
| Zambia | 8381 | 8505 | 8799 | 9154 | 9437 |
| Zimbabwe | 7905 | 7980 | 8212 | 8607 | 8649 |
