# Supplementary material for: Association between achieving adequate antenatal care and health-seeking behaviors: A study of Demographic and Health Surveys in 47 low- and middle-income countries
Source: PLoS Med. 2024 Jul 5;21(7):e1004421. doi: 10.1371/journal.pmed.1004421 (PMC11226092; doi:10.1371/journal.pmed.1004421)
Supplement: S6 Table — (DOCX) [file pmed.1004421.s006.docx]

**S6 Table.** Baseline unweighted absolute postnatal care utilization rates (per 10,000) across wealth quintiles and countries.

| **Country** | **Poorest** | **Poorer** | **Middle** | **Richer** | **Richest** |
| --- | --- | --- | --- | --- | --- |
| Angola | 1406 | 1427 | 2421 | 2313 | 3341 |
| Bangladesh | 5074 | 5255 | 5661 | 6129 | 6802 |
| Benin | 3255 | 3548 | 3849 | 4338 | 4842 |
| Burkina Faso | 7492 | 8158 | 8539 | 8650 | 8523 |
| Burundi | 1188 | 956 | 1002 | 1157 | 1765 |
| Cambodia | 4777 | 5368 | 5812 | 6248 | 6772 |
| Cameroon | 1463 | 2388 | 2796 | 2931 | 3479 |
| Chad | 1282 | 1338 | 1074 | 1128 | 2947 |
| Comoros | 2691 | 2869 | 3303 | 3894 | 3801 |
| Congo | 4900 | 5545 | 5274 | 6088 | 6642 |
| Congo, Democratic Republic of | 1208 | 1456 | 1496 | 1761 | 2638 |
| Côte d'Ivoire | 6780 | 7299 | 7331 | 7466 | 7278 |
| Dominican Republic | 9077 | 9470 | 9669 | 9750 | 9921 |
| Egypt | 3216 | 3343 | 3642 | 3459 | 3904 |
| Ethiopia | 405 | 592 | 644 | 869 | 1389 |
| Gabon | 5034 | 6232 | 6318 | 6265 | 7253 |
| Gambia | 6988 | 6699 | 6321 | 5923 | 4599 |
| Ghana | 7666 | 7112 | 6936 | 7211 | 7902 |
| Guatemala | 8779 | 8855 | 8938 | 9061 | 9542 |
| Guinea | 3376 | 4224 | 4364 | 5098 | 5265 |
| Haiti | 5197 | 5929 | 6906 | 7547 | 8259 |
| Honduras | 9005 | 9529 | 9746 | 9825 | 9840 |
| India | 4554 | 4465 | 4577 | 4625 | 4925 |
| Jordan | 4518 | 4689 | 5606 | 5278 | 6135 |
| Kenya | 4105 | 4966 | 4894 | 4951 | 5459 |
| Lesotho | 6655 | 7873 | 8314 | 8553 | 8981 |
| Liberia | 4805 | 4855 | 4751 | 4631 | 4523 |
| Madagascar | 2491 | 2625 | 3436 | 3517 | 4725 |
| Malawi | 3804 | 3929 | 4069 | 4610 | 5044 |
| Maldives | 7204 | 7329 | 7233 | 7778 | 9109 |
| Mali | 2519 | 2616 | 2557 | 3268 | 4739 |
| Mauritania | 2034 | 2335 | 2432 | 1824 | 1544 |
| Myanmar | 4283 | 4824 | 5036 | 5000 | 5019 |
| Nepal | 2860 | 3476 | 3667 | 4195 | 5108 |
| Niger | 5948 | 6694 | 6493 | 6418 | 7445 |
| Nigeria | 1054 | 1691 | 2766 | 3738 | 4908 |
| Pakistan | 3065 | 3019 | 3379 | 4133 | 5351 |
| Rwanda | 2977 | 3130 | 3420 | 3321 | 3317 |
| Sierra Leone | 5534 | 6097 | 6005 | 6314 | 5498 |
| South Africa | 8092 | 8249 | 8503 | 8667 | 8138 |
| Tanzania | 2504 | 2861 | 2943 | 3259 | 3608 |
| Timor Leste | 3038 | 2909 | 3304 | 3047 | 2979 |
| Togo | 7328 | 7094 | 7878 | 7463 | 7450 |
| Uganda | 2902 | 2576 | 1827 | 2198 | 3174 |
| Zambia | 5751 | 5809 | 6065 | 6275 | 6406 |
| Zimbabwe | 6889 | 6946 | 7414 | 8073 | 8429 |
