# Supplementary material for: Association between achieving adequate antenatal care and health-seeking behaviors: A study of Demographic and Health Surveys in 47 low- and middle-income countries
Source: PLoS Med. 2024 Jul 5;21(7):e1004421. doi: 10.1371/journal.pmed.1004421 (PMC11226092; doi:10.1371/journal.pmed.1004421)
Supplement: S7 Table — (DOCX) [file pmed.1004421.s007.docx]

**S7 Table.** Baseline unweighted absolute stunting rates (per 10,000) across wealth quintiles and countries.

| **Country** | **Poorest** | **Poorer** | **Middle** | **Richer** | **Richest** |
| --- | --- | --- | --- | --- | --- |
| Bangladesh | 4673 | 3904 | 3586 | 3032 | 2125 |
| Benin | 4213 | 3991 | 3647 | 3445 | 2646 |
| Burkina Faso | 3852 | 3270 | 3429 | 3095 | 1942 |
| Burundi | 6574 | 5765 | 5469 | 4655 | 3046 |
| Cambodia | 3948 | 3584 | 3340 | 2828 | 2077 |
| Cameroon | 3974 | 3689 | 2659 | 2121 | 1161 |
| Chad | 3952 | 4059 | 4186 | 4591 | 3197 |
| Comoros | 3421 | 2632 | 2239 | 2444 | 2030 |
| Congo | 3179 | 2377 | 2027 | 1307 | 947 |
| Congo, Democratic Republic of | 4110 | 4236 | 3929 | 3593 | 2000 |
| Côte d'Ivoire | 3345 | 3211 | 2643 | 2277 | 1614 |
| Dominican Republic | 1238 | 785 | 429 | 532 | 350 |
| Egypt | 2173 | 2109 | 1790 | 1923 | 1811 |
| Ethiopia | 4229 | 4396 | 3912 | 3766 | 2429 |
| Gabon | 3016 | 2029 | 1563 | 1051 | 573 |
| Gambia | 2609 | 2296 | 2159 | 1709 | 1392 |
| Ghana | 2146 | 2084 | 1384 | 1411 | 769 |
| Guatemala | 6060 | 5335 | 4100 | 2603 | 1680 |
| Guinea | 3519 | 3256 | 3440 | 2605 | 1731 |
| Haiti | 2715 | 2176 | 1616 | 1491 | 825 |
| Honduras | 3858 | 2445 | 1499 | 1033 | 664 |
| India | 4209 | 3700 | 3261 | 2745 | 2232 |
| Kenya | 2977 | 2562 | 2021 | 1760 | 1109 |
| Lesotho | 4208 | 3289 | 3484 | 2514 | 1627 |
| Liberia | 3023 | 3061 | 2923 | 2580 | 1570 |
| Madagascar | 4013 | 3871 | 3690 | 3763 | 2915 |
| Malawi | 4458 | 4181 | 3946 | 3658 | 2740 |
| Maldives | 1805 | 1569 | 1465 | 1233 | 1273 |
| Mali | 4063 | 3511 | 3542 | 2693 | 1814 |
| Mauritania | 3262 | 3069 | 2556 | 2142 | 1201 |
| Mozambique | 4863 | 4562 | 4163 | 3557 | 2281 |
| Myanmar | 3569 | 2984 | 3025 | 2074 | 1630 |
| Nepal | 4512 | 3597 | 2830 | 2584 | 1888 |
| Niger | 4199 | 4242 | 3955 | 4208 | 3200 |
| Nigeria | 5145 | 4316 | 3392 | 2381 | 1678 |
| Pakistan | 5091 | 4198 | 3483 | 2921 | 2328 |
| Rwanda | 4690 | 4402 | 3779 | 3163 | 1768 |
| Sierra Leone | 3650 | 3461 | 3547 | 2986 | 2311 |
| South Africa | 3448 | 2442 | 2658 | 1859 | 1333 |
| Tanzania | 4080 | 3846 | 3789 | 3199 | 2213 |
| Timor Leste | 5366 | 5277 | 4977 | 4629 | 4193 |
| Togo | 3014 | 3105 | 2905 | 1895 | 1226 |
| Uganda | 3364 | 2918 | 3293 | 2600 | 1669 |
| Zambia | 4158 | 3931 | 3596 | 3528 | 2499 |
| Zimbabwe | 3323 | 2920 | 3049 | 2776 | 1913 |
