# Supplementary material for: Association between achieving adequate antenatal care and health-seeking behaviors: A study of Demographic and Health Surveys in 47 low- and middle-income countries
Source: PLoS Med. 2024 Jul 5;21(7):e1004421. doi: 10.1371/journal.pmed.1004421 (PMC11226092; doi:10.1371/journal.pmed.1004421)
Supplement: S8 Table — (DOCX) [file pmed.1004421.s008.docx]

**S8 Table.** Baseline unweighted absolute wasting rates (per 10,000) across wealth quintiles and countries.

| **Country** | **Poorest** | **Poorer** | **Middle** | **Richer** | **Richest** |
| --- | --- | --- | --- | --- | --- |
| Bangladesh | 1709 | 1474 | 1322 | 1180 | 1015 |
| Benin | 1404 | 1289 | 1303 | 1115 | 1035 |
| Burkina Faso | 2210 | 1921 | 1843 | 1826 | 1338 |
| Burundi | 866 | 749 | 639 | 625 | 431 |
| Cambodia | 1744 | 1436 | 1435 | 1335 | 1020 |
| Cameroon | 1127 | 706 | 458 | 321 | 277 |
| Chad | 1632 | 1579 | 1757 | 1709 | 1704 |
| Comoros | 1577 | 1439 | 1245 | 1036 | 914 |
| Congo | 685 | 609 | 507 | 578 | 700 |
| Congo, Democratic Republic of | 1116 | 1083 | 1101 | 891 | 491 |
| Côte d'Ivoire | 1007 | 847 | 891 | 681 | 712 |
| Dominican Republic | 305 | 231 | 199 | 206 | 225 |
| Egypt | 857 | 911 | 1117 | 1407 | 1215 |
| Ethiopia | 1815 | 1364 | 1231 | 1035 | 754 |
| Gabon | 519 | 355 | 336 | 389 | 385 |
| Gambia | 778 | 1010 | 1073 | 781 | 632 |
| Ghana | 874 | 520 | 261 | 629 | 347 |
| Guatemala | 96 | 65 | 81 | 88 | 87 |
| Guinea | 1240 | 1135 | 1018 | 1053 | 838 |
| Haiti | 546 | 473 | 499 | 517 | 329 |
| Honduras | 214 | 137 | 150 | 103 | 135 |
| India | 2401 | 2080 | 1922 | 1794 | 1586 |
| Kenya | 1580 | 514 | 720 | 624 | 464 |
| Lesotho | 586 | 543 | 317 | 337 | 120 |
| Liberia | 629 | 559 | 684 | 723 | 822 |
| Madagascar | 1028 | 745 | 718 | 550 | 742 |
| Malawi | 460 | 419 | 467 | 347 | 341 |
| Maldives | 937 | 972 | 843 | 833 | 1296 |
| Mali | 1742 | 1596 | 1300 | 1130 | 1046 |
| Mauritania | 978 | 809 | 851 | 571 | 291 |
| Mozambique | 1231 | 792 | 603 | 441 | 257 |
| Myanmar | 863 | 609 | 714 | 586 | 887 |
| Nepal | 1594 | 1438 | 1438 | 1510 | 962 |
| Niger | 2708 | 2341 | 1979 | 2229 | 1604 |
| Nigeria | 2134 | 1813 | 1498 | 1324 | 1163 |
| Pakistan | 1471 | 1137 | 975 | 945 | 713 |
| Rwanda | 283 | 257 | 260 | 179 | 217 |
| Sierra Leone | 769 | 770 | 759 | 940 | 817 |
| South Africa | 276 | 288 | 374 | 268 | 137 |
| Tanzania | 608 | 585 | 679 | 639 | 559 |
| Timor Leste | 2538 | 2488 | 2339 | 2190 | 1817 |
| Togo | 1088 | 862 | 835 | 745 | 588 |
| Uganda | 831 | 638 | 499 | 300 | 340 |
| Zambia | 638 | 579 | 458 | 634 | 555 |
| Zimbabwe | 491 | 471 | 335 | 419 | 170 |
