# Supplementary material for: Association between achieving adequate antenatal care and health-seeking behaviors: A study of Demographic and Health Surveys in 47 low- and middle-income countries
Source: PLoS Med. 2024 Jul 5;21(7):e1004421. doi: 10.1371/journal.pmed.1004421 (PMC11226092; doi:10.1371/journal.pmed.1004421)
Supplement: S9 Table — (DOCX) [file pmed.1004421.s009.docx]

**S9 Table.** Facility birth rate change (per 10,000) (with 95% confidence interval and p-value) associated with achieving recommended antenatal care visits and quality.

| **Country** | **Poorest** | **Poorer** | **Middle** | **Richer** | **Richest** |
| --- | --- | --- | --- | --- | --- |
| Angola | 1332 (1200, 1464) (p<0.001) | 1200 (1020, 1380) (p<0.001) | 764 (661, 866) (p<0.001) | 294 (186, 401) (p<0.001) | 116 (56, 176)  (p<0.001) |
| Bangladesh | 2022 (1861, 2183) (p<0.001) | 2168 (2022, 2313) (p<0.001) | 2336 (2104, 2567) (p<0.001) | 1759 (1600, 1919) (p<0.001) | 1098 (996, 1200) (p<0.001) |
| Benin | 2278 (2108, 2447) (p<0.001) | 1408 (1274, 1542) (p<0.001) | 806 (696, 916) (p<0.001) | 202 (159, 244) (p<0.001) | 15 (-7, 37)  (p=0.179) |
| Burkina Faso | 2647 (1941, 3352) (p<0.001) | 2006 (1432, 2580) (p<0.001) | 1085 (378, 1791) (p=0.003) | 765 (335, 1196) (p<0.001) | 196 (87, 305)  (p<0.001) |
| Burundi | 1344 (1060, 1628) (p<0.001) | 620 (172, 1068) (p=0.007) | 1098 (801, 1395) (p<0.001) | 1361 (1042, 1680) (p<0.001) | 411 (221, 600) (p<0.001) |
| Cambodia | 2284 (2097, 2472) (p<0.001) | 1749 (1556, 1942) (p<0.001) | 1312 (1104, 1520) (p<0.001) | 812 (700, 925) (p<0.001) | 289 (210, 369) (p<0.001) |
| Cameroon | 1834 (1646, 2022) (p<0.001) | 1501 (1345, 1658) (p<0.001) | 869 (764, 974) (p<0.001) | 295 (244, 346) (p<0.001) | 61 (34, 87)  (p<0.001) |
| Chad | 1569 (1192, 1946) (p<0.001) | 1878 (1498, 2258) (p<0.001) | 1539 (1202, 1876) (p<0.001) | 1578 (1222, 1934) (p<0.001) | 1443 (1234, 1651) (p<0.001) |
| Comoros | 989 (627, 1351) (p<0.001) | 530 (157, 903) (p=0.005) | 420 (180, 661) (p<0.001) | -79 (-333, 175) (p=0.553) | 106 (-78, 289) (p=0.261) |
| Congo | 930 (723, 1136) (p<0.001) | 353 (212, 493) (p<0.001) | 108 (42, 173)  (p=0.001) | 21 (8, 35)  (p=0.002) | 1 (-4, 5)  (p=0.82) |
| Congo, Democratic Republic of | 1890 (1489, 2292) (p<0.001) | 1112 (691, 1532) (p<0.001) | 891 (615, 1168) (p<0.001) | 397 (212, 583) (p<0.001) | 79 (29, 129)  (p=0.002) |
| Côte d'Ivoire | 1983 (1526, 2441) (p<0.001) | 1430 (1075, 1786) (p<0.001) | 866 (502, 1230) (p<0.001) | 444 (213, 675) (p<0.001) | 282 (71, 494)  (p=0.009) |
| Dominican Republic | 117 (63, 171)  (p<0.001) | 8 (0, 17)  (p=0.045) | 10 (-16, 36)  (p=0.458) | 10 (-14, 34)  (p=0.422) | 4 (-2, 10)  (p=0.193) |
| Egypt | 129 (9, 249)  (p=0.035) | 115 (3, 227)  (p=0.045) | 26 (-51, 104)  (p=0.515) | 3 (-28, 34)  (p=0.842) | 9 (1, 17)  (p=0.02) |
| Ethiopia | 2722 (2373, 3071) (p<0.001) | 3372 (2844, 3899) (p<0.001) | 2230 (1847, 2614) (p<0.001) | 1999 (1588, 2411) (p<0.001) | 1535 (1362, 1709) (p<0.001) |
| Gabon | 1002 (885, 1119) (p<0.001) | 220 (149, 291) (p<0.001) | 61 (6, 117)  (p=0.03) | 117 (31, 204)  (p=0.008) | 21 (7, 35)  (p=0.004) |
| Gambia | 181 (119, 243) (p<0.001) | 143 (79, 208)  (p<0.001) | 57 (-42, 157)  (p=0.261) | 94 (28, 161)  (p=0.005) | 38 (10, 67)  (p=0.009) |
| Ghana | 494 (378, 609) (p<0.001) | 356 (218, 494) (p<0.001) | 155 (73, 236)  (p<0.001) | 59 (17, 100)  (p=0.005) | 32 (-26, 90)  (p=0.285) |
| Guatemala | 1231 (1034, 1427) (p<0.001) | 941 (751, 1130) (p<0.001) | 647 (530, 764) (p<0.001) | 185 (118, 252) (p<0.001) | 121 (58, 184)  (p<0.001) |
| Guinea | 1683 (1350, 2017) (p<0.001) | 1035 (641, 1429) (p<0.001) | 1437 (1120, 1754) (p<0.001) | 676 (426, 925) (p<0.001) | 199 (114, 284) (p<0.001) |
| Haiti | 459 (378, 541) (p<0.001) | 575 (436, 714) (p<0.001) | 597 (493, 700) (p<0.001) | 472 (378, 566) (p<0.001) | 241 (151, 331) (p<0.001) |
| Honduras | 706 (610, 803) (p<0.001) | 387 (290, 483) (p<0.001) | 177 (125, 229) (p<0.001) | 73 (43, 103)  (p<0.001) | 33 (7, 58)  (p=0.011) |
| India | 349 (329, 369) (p<0.001) | 182 (169, 196) (p<0.001) | 126 (116, 135) (p<0.001) | 65 (57, 73)  (p<0.001) | 28 (24, 32)  (p<0.001) |
| Jordan | 29 (15, 43)  (p<0.001) | 16 (6, 25)  (p=0.001) | 3 (-1, 7)  (p=0.137) | 4 (0, 7)  (p=0.029) | -5 (-13, 3)  (p=0.191) |
| Kenya | 808 (726, 890) (p<0.001) | 619 (497, 741) (p<0.001) | 478 (393, 563) (p<0.001) | 238 (192, 284) (p<0.001) | 72 (51, 93)  (p<0.001) |
| Lesotho | 480 (215, 745) (p<0.001) | 554 (173, 934) (p=0.004) | 266 (89, 443)  (p=0.003) | 42 (-90, 173)  (p=0.544) | 96 (33, 159)  (p=0.003) |
| Liberia | 779 (652, 906) (p<0.001) | 517 (380, 653) (p<0.001) | 259 (148, 369) (p<0.001) | 247 (148, 347) (p<0.001) | 90 (-1, 181)  (p=0.052) |
| Madagascar | 1335 (726, 1945) (p<0.001) | 1717 (1208, 2225) (p<0.001) | 746 (259, 1233) (p=0.003) | 796 (353, 1238) (p<0.001) | 512 (227, 797) (p<0.001) |
| Malawi | 952 (815, 1088) (p<0.001) | 752 (619, 886) (p<0.001) | 568 (365, 770) (p<0.001) | 572 (463, 681) (p<0.001) | 87 (51, 124)  (p<0.001) |
| Maldives | 28 (11, 45)  (p=0.002) | 9 (-5, 23)  (p=0.205) | 12 (-27, 50)  (p=0.562) | 25 (-10, 60)  (p=0.156) | -7 (-26, 11)  (p=0.454) |
| Mali | 3016 (2611, 3421) (p<0.001) | 2315 (1760, 2870) (p<0.001) | 2179 (1829, 2529) (p<0.001) | 1373 (1196, 1551) (p<0.001) | 262 (216, 308) (p<0.001) |
| Mauritania | 2241 (1668, 2813) (p<0.001) | 1988 (1528, 2448) (p<0.001) | 664 (471, 857) (p<0.001) | 153 (38, 269)  (p=0.009) | 73 (4, 141)  (p=0.037) |
| Mozambique | 1339 (979, 1699) (p<0.001) | 1420 (975, 1864) (p<0.001) | 1076 (778, 1374) (p<0.001) | 741 (518, 964) (p<0.001) | 121 (15, 227)  (p=0.024) |
| Myanmar | 1257 (1104, 1411) (p<0.001) | 1042 (803, 1281) (p<0.001) | 1025 (696, 1354) (p<0.001) | 718 (430, 1007) (p<0.001) | 443 (238, 648) (p<0.001) |
| Nepal | 2192 (1947, 2436) (p<0.001) | 1862 (1631, 2093) (p<0.001) | 1534 (1333, 1736) (p<0.001) | 894 (720, 1069) (p<0.001) | 387 (231, 543) (p<0.001) |
| Niger | 1926 (891, 2961) (p<0.001) | 1317 (458, 2176) (p=0.003) | 981 (-102, 2063) (p=0.076) | 1720 (1122, 2318) (p<0.001) | 974 (736, 1212) (p<0.001) |
| Nigeria | 1111 (985, 1238) (p<0.001) | 1023 (882, 1164) (p<0.001) | 1041 (946, 1137) (p<0.001) | 678 (614, 742) (p<0.001) | 405 (344, 467) (p<0.001) |
| Pakistan | 1835 (1336, 2335) (p<0.001) | 1981 (1587, 2375) (p<0.001) | 1126 (799, 1453) (p<0.001) | 579 (350, 808) (p<0.001) | 221 (120, 323) (p<0.001) |
| Rwanda | 979 (802, 1157) (p<0.001) | 728 (536, 920) (p<0.001) | 801 (631, 971) (p<0.001) | 693 (558, 828) (p<0.001) | 270 (196, 344) (p<0.001) |
| Sierra Leone | 453 (380, 527) (p<0.001) | 385 (254, 516) (p<0.001) | 325 (254, 397) (p<0.001) | 249 (147, 352) (p<0.001) | 109 (2, 217)  (p=0.046) |
| South Africa | 65 (-5, 135)  (p=0.069) | 30 (-9, 70)  (p=0.132) | 15 (0, 29)  (p=0.053) | 7 (-4, 18)  (p=0.217) | 0 (-2, 2)  (p=0.998) |
| Tanzania | 819 (301, 1337) (p=0.002) | 932 (483, 1380) (p<0.001) | 533 (136, 931) (p=0.009) | 93 (-169, 356) (p=0.496) | 112 (-53, 278) (p=0.185) |
| Timor Leste | 781 (560, 1003) (p<0.001) | 1329 (974, 1684) (p<0.001) | 1778 (1386, 2169) (p<0.001) | 1741 (1417, 2064) (p<0.001) | 885 (696, 1074) (p<0.001) |
| Togo | 1735 (882, 2588) (p<0.001) | 1358 (501, 2215) (p=0.002) | 1066 (518, 1615) (p<0.001) | 211 (105, 317) (p<0.001) | 167 (47, 288)  (p=0.007) |
| Uganda | 1272 (881, 1663) (p<0.001) | 1621 (1179, 2063) (p<0.001) | 1371 (918, 1823) (p<0.001) | 1191 (795, 1587) (p<0.001) | 362 (234, 490) (p<0.001) |
| Zambia | 1324 (1186, 1463) (p<0.001) | 657 (515, 798) (p<0.001) | 633 (506, 760) (p<0.001) | 255 (169, 342) (p<0.001) | 120 (73, 168)  (p<0.001) |
| Zimbabwe | 1599 (1423, 1774) (p<0.001) | 1279 (1120, 1439) (p<0.001) | 1104 (940, 1268) (p<0.001) | 507 (386, 627) (p<0.001) | 239 (157, 320) (p<0.001) |
