# Supplementary material for: Association between achieving adequate antenatal care and health-seeking behaviors: A study of Demographic and Health Surveys in 47 low- and middle-income countries
Source: PLoS Med. 2024 Jul 5;21(7):e1004421. doi: 10.1371/journal.pmed.1004421 (PMC11226092; doi:10.1371/journal.pmed.1004421)
Supplement: S10 Table — (DOCX) [file pmed.1004421.s010.docx]

**S10 Table.** Diphtheria-pertussis-tetanus 3rd dose (DPT3) vaccination rate change (per 10,000) (with 95% confidence interval and p-value) associated with achieving recommended antenatal care visits and quality.

| **Country** | **Poorest** | **Poorer** | **Middle** | **Richer** | **Richest** |
| --- | --- | --- | --- | --- | --- |
| Angola | 1336 (1126, 1546) (p<0.001) | 1061 (855, 1267) (p<0.001) | 542 (404, 680) (p<0.001) | 330 (136, 524) (p<0.001) | 99 (6, 191)  (p=0.036) |
| Bangladesh | 510 (427, 593) (p<0.001) | 362 (303, 420) (p<0.001) | 294 (246, 342) (p<0.001) | 201 (160, 243) (p<0.001) | 78 (58, 97)  (p<0.001) |
| Benin | 1767 (1533, 2000) (p<0.001) | 954 (771, 1138) (p<0.001) | 561 (435, 687) (p<0.001) | 224 (147, 300) (p<0.001) | 177 (117, 237) (p<0.001) |
| Burkina Faso | 1049 (871, 1228) (p<0.001) | -100 (-900, 700) (p=0.818) | 629 (512, 746) (p<0.001) | 398 (294, 503) (p<0.001) | 280 (217, 343) (p<0.001) |
| Burundi | 217 (170, 264) (p<0.001) | 142 (106, 178) (p<0.001) | 109 (78, 141)  (p<0.001) | 65 (37, 94)  (p<0.001) | -11 (-382, 360) (p=0.959) |
| Cambodia | 738 (583, 893) (p<0.001) | 442 (281, 602) (p<0.001) | 424 (311, 537) (p<0.001) | 132 (72, 192)  (p<0.001) | 116 (60, 172)  (p<0.001) |
| Cameroon | 1565 (1401, 1729) (p<0.001) | 718 (590, 846) (p<0.001) | 414 (332, 496) (p<0.001) | 156 (113, 199) (p<0.001) | 50 (30, 70)  (p<0.001) |
| Chad | 1993 (1467, 2518) (p<0.001) | 1469 (697, 2242) (p<0.001) | 1296 (880, 1711) (p<0.001) | 1525 (1074, 1976) (p<0.001) | 325 (41, 608)  (p=0.025) |
| Comoros | 715 (296, 1135) (p<0.001) | 334 (-26, 694) (p=0.068) | 147 (-19, 313) (p=0.082) | 305 (52, 558)  (p=0.018) | 150 (-35, 334) (p=0.111) |
| Congo | 655 (323, 988) (p<0.001) | 526 (129, 923) (p=0.009) | 148 (-35, 330) (p=0.114) | 49 (1, 97)  (p=0.044) | 11 (-15, 38)  (p=0.41) |
| Congo, Democratic Republic of | 1409 (1185, 1633) (p<0.001) | 681 (88, 1274) (p=0.024) | 1181 (725, 1637) (p<0.001) | 610 (223, 997) (p=0.002) | 278 (49, 506)  (p=0.017) |
| Côte d'Ivoire | 1330 (1080, 1581) (p<0.001) | 2011 (1325, 2697) (p<0.001) | 519 (327, 710) (p<0.001) | 413 (8, 817)  (p=0.045) | 123 (28, 219)  (p=0.011) |
| Dominican Republic | 88 (31, 146)  (p=0.003) | 42 (2, 83)  (p=0.041) | 6 (-2, 14)  (p=0.162) | 0 (0, 0)  (NA) | 0 (0, 0)  (NA) |
| Egypt | 42 (25, 59)  (p<0.001) | 36 (23, 48)  (p<0.001) | 25 (16, 35)  (p<0.001) | 19 (10, 28)  (p<0.001) | 6 (3, 9)  (p<0.001) |
| Ethiopia | 2664 (2447, 2882) (p<0.001) | 2470 (1765, 3174) (p<0.001) | 1973 (1740, 2205) (p<0.001) | 1144 (691, 1598) (p<0.001) | 1047 (733, 1361) (p<0.001) |
| Gabon | 306 (170, 441) (p<0.001) | 111 (-1, 224)  (p=0.052) | 95 (5, 185)  (p=0.039) | 70 (-186, 325) (p=0.606) | 22 (-4, 48)  (p=0.1) |
| Gambia | 67 (48, 86)  (p<0.001) | 40 (25, 54)  (p<0.001) | 54 (35, 73)  (p<0.001) | -10 (-39, 19)  (p=0.494) | -13 (-28, 1)  (p=0.075) |
| Ghana | 233 (118, 348) (p<0.001) | 196 (50, 342)  (p=0.008) | 95 (4, 185)  (p=0.04) | -5 (-20, 11)  (p=0.552) | 4 (-1, 9)  (p=0.125) |
| Guatemala | 192 (-44, 428) (p=0.111) | 148 (-21, 316) (p=0.085) | 202 (82, 321)  (p=0.001) | 37 (-10, 85)  (p=0.123) | 17 (-6, 40)  (p=0.158) |
| Guinea | 961 (420, 1502) (p<0.001) | 1255 (527, 1984) (p<0.001) | 242 (-213, 698) (p=0.301) | 332 (-43, 707) (p=0.083) | 142 (56, 229)  (p=0.001) |
| Haiti | 916 (739, 1092) (p<0.001) | 740 (485, 995) (p<0.001) | 415 (295, 534) (p<0.001) | 377 (244, 511) (p<0.001) | 174 (62, 286)  (p=0.002) |
| Honduras | 61 (40, 82)  (p<0.001) | 42 (28, 57)  (p<0.001) | 37 (14, 60)  (p=0.002) | 18 (6, 30)  (p=0.003) | 3 (0, 6)  (p=0.023) |
| India | 188 (166, 210) (p<0.001) | 132 (117, 147) (p<0.001) | 84 (74, 93)  (p<0.001) | 66 (56, 76)  (p<0.001) | 32 (21, 42)  (p<0.001) |
| Jordan | 46 (21, 72)  (p<0.001) | 25 (4, 46)  (p=0.019) | 10 (2, 18)  (p=0.011) | 9 (-3, 21)  (p=0.153) | 3 (-15, 20)  (p=0.755) |
| Kenya | 317 (263, 371) (p<0.001) | 106 (68, 145)  (p<0.001) | 97 (60, 133)  (p<0.001) | 17 (-19, 53)  (p=0.352) | 13 (0, 27)  (p=0.058) |
| Lesotho | 151 (81, 221)  (p<0.001) | 105 (54, 156)  (p<0.001) | 102 (38, 166)  (p=0.002) | 34 (12, 56)  (p=0.003) | -64 (-518, 391) (p=0.796) |
| Liberia | 361 (279, 444) (p<0.001) | 467 (275, 659) (p<0.001) | 133 (-5, 271)  (p=0.059) | 91 (2, 180)  (p=0.044) | 81 (-11, 174)  (p=0.085) |
| Madagascar | 1407 (953, 1862) (p<0.001) | 566 (-552, 1684) (p=0.326) | -260 (-895, 376) (p=0.431) | -26 (-257, 204) (p=0.835) | 68 (-15, 151)  (p=0.106) |
| Malawi | 224 (183, 265) (p<0.001) | 163 (121, 206) (p<0.001) | 126 (96, 157)  (p<0.001) | 72 (45, 98)  (p<0.001) | 82 (65, 99)  (p<0.001) |
| Maldives | 62 (-10, 134)  (p=0.093) | 48 (11, 84)  (p=0.011) | 45 (0, 90)  (p=0.049) | 30 (-9, 70)  (p=0.135) | 0 (0, 0)  (NA) |
| Mali | 2270 (1981, 2560) (p<0.001) | 1649 (1369, 1928) (p<0.001) | 871 (386, 1357) (p<0.001) | 611 (160, 1061) (p=0.008) | 427 (303, 550) (p<0.001) |
| Mauritania | 937 (441, 1433) (p<0.001) | 1295 (687, 1903) (p<0.001) | 449 (205, 693) (p<0.001) | 244 (141, 347) (p<0.001) | 21 (-157, 200) (p=0.825) |
| Mozambique | 1653 (1332, 1974) (p<0.001) | 1311 (1044, 1579) (p<0.001) | 1236 (983, 1489) (p<0.001) | 523 (186, 860) (p=0.002) | 372 (293, 451) (p<0.001) |
| Myanmar | 1056 (745, 1366) (p<0.001) | 759 (515, 1004) (p<0.001) | 610 (-131, 1351) (p=0.107) | 333 (104, 562) (p=0.004) | 30 (-16, 77)  (p=0.203) |
| Nepal | 335 (249, 422) (p<0.001) | 221 (161, 281) (p<0.001) | 127 (-25, 278) (p=0.101) | 83 (55, 112)  (p<0.001) | 21 (9, 33)  (p<0.001) |
| Niger | 1171 (987, 1356) (p<0.001) | 576 (-412, 1565) (p=0.256) | 709 (36, 1383) (p=0.039) | 439 (285, 593) (p<0.001) | 350 (265, 434) (p<0.001) |
| Nigeria | 1570 (1403, 1737) (p<0.001) | 1166 (883, 1450) (p<0.001) | 869 (751, 987) (p<0.001) | 446 (368, 525) (p<0.001) | 227 (133, 320) (p<0.001) |
| Pakistan | 948 (763, 1132) (p<0.001) | 590 (444, 736) (p<0.001) | 586 (132, 1041) (p=0.011) | 408 (161, 656) (p=0.001) | 58 (23, 94)  (p=0.001) |
| Rwanda | 87 (63, 111)  (p<0.001) | 52 (37, 68)  (p<0.001) | 38 (26, 49)  (p<0.001) | 25 (15, 34)  (p<0.001) | 27 (19, 35)  (p<0.001) |
| Sierra Leone | 254 (155, 354) (p<0.001) | 121 (32, 209)  (p=0.007) | 90 (10, 170)  (p=0.027) | -22 (-109, 65) (p=0.632) | 41 (-50, 132)  (p=0.384) |
| South Africa | 74 (-4, 152)  (p=0.062) | 24 (-32, 81)  (p=0.407) | 78 (-63, 219)  (p=0.283) | 20 (-110, 149) (p=0.779) | 105 (6, 204)  (p=0.038) |
| Tanzania | 358 (61, 654)  (p=0.018) | 130 (-281, 542) (p=0.545) | 79 (-139, 298) (p=0.486) | -10 (-120, 99) (p=0.862) | 112 (31, 193)  (p=0.007) |
| Timor Leste | 1451 (1001, 1902) (p<0.001) | 917 (530, 1303) (p<0.001) | 903 (539, 1268) (p<0.001) | 382 (68, 695)  (p=0.017) | 400 (97, 702)  (p=0.01) |
| Togo | -312 (-1009, 384) (p=0.386) | -604 (-2150, 942) (p=0.453) | -476 (-1156, 204) (p=0.171) | -459 (-832, -85) (p=0.016) | 0 (-34, 34)  (p=0.996) |
| Uganda | 637 (455, 820) (p<0.001) | 119 (-497, 735) (p=0.717) | -67 (-531, 398) (p=0.791) | 262 (85, 439)  (p=0.004) | 295 (138, 452) (p<0.001) |
| Zambia | 506 (442, 570) (p<0.001) | 277 (228, 326) (p<0.001) | 193 (153, 233) (p<0.001) | 147 (50, 243)  (p=0.003) | 74 (60, 88)  (p<0.001) |
| Zimbabwe | 1073 (815, 1331) (p<0.001) | 760 (538, 982) (p<0.001) | 649 (433, 864) (p<0.001) | 397 (250, 545) (p<0.001) | 235 (72, 398)  (p=0.005) |
