# Supplementary material for: Association between achieving adequate antenatal care and health-seeking behaviors: A study of Demographic and Health Surveys in 47 low- and middle-income countries
Source: PLoS Med. 2024 Jul 5;21(7):e1004421. doi: 10.1371/journal.pmed.1004421 (PMC11226092; doi:10.1371/journal.pmed.1004421)
Supplement: S11 Table — (DOCX) [file pmed.1004421.s011.docx]

**S11 Table.** Measles vaccination rate change (per 10,000) (with 95% confidence interval and p-value) associated with achieving recommended antenatal care visits and quality.

| **Country** | **Poorest** | **Poorer** | **Middle** | **Richer** | **Richest** |
| --- | --- | --- | --- | --- | --- |
| Angola | 1691 (1455, 1927) (p<0.001) | 1117 (908, 1326) (p<0.001) | 637 (489, 785) (p<0.001) | 394 (178, 609) (p<0.001) | 85 (0, 171)  (p=0.05) |
| Bangladesh | 803 (690, 915) (p<0.001) | 609 (528, 689) (p<0.001) | 452 (378, 526) (p<0.001) | 338 (283, 393) (p<0.001) | 136 (105, 167) (p<0.001) |
| Benin | 1746 (1500, 1992) (p<0.001) | 1000 (771, 1230) (p<0.001) | 667 (531, 802) (p<0.001) | 316 (230, 401) (p<0.001) | 194 (141, 247) (p<0.001) |
| Burkina Faso | 1299 (693, 1905) (p<0.001) | 113 (-657, 883) (p=0.786) | 509 (212, 806) (p<0.001) | 46 (-494, 587) (p=0.876) | 341 (175, 506) (p<0.001) |
| Burundi | 493 (365, 621) (p<0.001) | 299 (203, 394) (p<0.001) | 290 (188, 393) (p<0.001) | -170 (-698, 357) (p=0.538) | 180 (126, 233) (p<0.001) |
| Cambodia | 656 (514, 799) (p<0.001) | 532 (175, 888) (p=0.004) | 411 (113, 710) (p=0.007) | 146 (88, 204)  (p<0.001) | 103 (-57, 263) (p=0.207) |
| Cameroon | 1469 (1311, 1627) (p<0.001) | 645 (528, 763) (p<0.001) | 309 (235, 384) (p<0.001) | 149 (52, 247)  (p=0.003) | 42 (25, 59)  (p<0.001) |
| Chad | 2148 (1622, 2674) (p<0.001) | 1399 (718, 2080) (p<0.001) | 2175 (1524, 2827) (p<0.001) | 1806 (1256, 2356) (p<0.001) | 778 (438, 1119) (p<0.001) |
| Comoros | 561 (102, 1020) (p=0.017) | 196 (-303, 695) (p=0.45) | 137 (-84, 358) (p=0.226) | 81 (-76, 238)  (p=0.315) | 68 (-50, 186)  (p=0.264) |
| Congo | 659 (300, 1019) (p<0.001) | 448 (179, 716) (p=0.001) | 200 (71, 328)  (p=0.002) | 93 (-170, 355) (p=0.498) | 12 (-2, 25)  (p=0.098) |
| Congo, Democratic Republic of | 1512 (1082, 1942) (p<0.001) | 771 (315, 1226) (p<0.001) | 947 (542, 1352) (p<0.001) | 568 (326, 810) (p<0.001) | 181 (86, 277)  (p<0.001) |
| Côte d'Ivoire | 1323 (1055, 1591) (p<0.001) | 1323 (866, 1781) (p<0.001) | 587 (398, 776) (p<0.001) | 533 (230, 835) (p<0.001) | 288 (86, 490)  (p=0.005) |
| Dominican Republic | 101 (15, 187)  (p=0.021) | 26 (-1, 53)  (p=0.059) | 4 (-1, 8)  (p=0.107) | 0 (0, 0)  (NA) | 0 (0, 0)  (NA) |
| Egypt | -70 (-227, 87) (p=0.389) | -80 (-238, 78) (p=0.326) | -59 (-170, 52) (p=0.303) | -50 (-162, 61) (p=0.384) | -17 (-68, 33)  (p=0.508) |
| Ethiopia | 2793 (2474, 3112) (p<0.001) | 1974 (1653, 2295) (p<0.001) | 1507 (913, 2101) (p<0.001) | 1161 (665, 1657) (p<0.001) | 758 (606, 909) (p<0.001) |
| Gabon | 340 (160, 520) (p<0.001) | 130 (49, 211)  (p=0.002) | 76 (24, 128)  (p=0.004) | 10 (-43, 63)  (p=0.719) | -30 (-96, 36)  (p=0.379) |
| Gambia | 57 (36, 79)  (p<0.001) | 43 (27, 59)  (p<0.001) | 41 (22, 60)  (p<0.001) | -4 (-26, 19)  (p=0.754) | 37 (-37, 110)  (p=0.332) |
| Ghana | 229 (113, 345) (p<0.001) | 195 (49, 341)  (p=0.009) | 100 (3, 197)  (p=0.043) | 11 (-7, 29)  (p=0.229) | 2 (-4, 8)  (p=0.469) |
| Guatemala | 81 (-210, 373) (p=0.597) | 63 (-143, 269) (p=0.561) | 17 (-167, 202) (p=0.863) | 34 (-37, 106)  (p=0.351) | 2 (-38, 42)  (p=0.919) |
| Guinea | 430 (-15, 874) (p=0.058) | 318 (-200, 835) (p=0.231) | -261 (-663, 141) (p=0.204) | 81 (-115, 277) (p=0.425) | 212 (93, 330)  (p<0.001) |
| Haiti | 624 (510, 737) (p<0.001) | 308 (177, 439) (p<0.001) | 273 (197, 349) (p<0.001) | 240 (148, 333) (p<0.001) | 83 (23, 144)  (p=0.007) |
| Honduras | 119 (76, 163)  (p<0.001) | 87 (55, 119)  (p<0.001) | 32 (11, 54)  (p=0.003) | 17 (-3, 36)  (p=0.09) | 6 (1, 11)  (p=0.031) |
| India | 176 (157, 194) (p<0.001) | 130 (116, 144) (p<0.001) | 68 (57, 78)  (p<0.001) | 60 (52, 68)  (p<0.001) | 36 (29, 44)  (p<0.001) |
| Jordan | 62 (19, 105)  (p=0.005) | 24 (-9, 56)  (p=0.157) | 11 (-5, 27)  (p=0.189) | 10 (-8, 28)  (p=0.266) | -3 (-21, 15)  (p=0.74) |
| Kenya | 399 (331, 468) (p<0.001) | 189 (80, 297)  (p<0.001) | 124 (70, 178)  (p<0.001) | 87 (59, 114)  (p<0.001) | 33 (23, 43)  (p<0.001) |
| Lesotho | 125 (49, 201)  (p=0.001) | 98 (38, 158)  (p=0.001) | 61 (9, 114)  (p=0.021) | 3 (-59, 65)  (p=0.935) | 46 (-9, 101)  (p=0.102) |
| Liberia | 365 (280, 450) (p<0.001) | 230 (154, 305) (p<0.001) | 181 (52, 310)  (p=0.006) | 79 (-6, 165)  (p=0.07) | 93 (16, 171)  (p=0.018) |
| Madagascar | 1661 (1157, 2165) (p<0.001) | 415 (-572, 1401) (p=0.418) | -387 (-960, 185) (p=0.186) | 236 (-184, 656) (p=0.274) | 161 (47, 274)  (p=0.006) |
| Malawi | 97 (-114, 309) (p=0.372) | -108 (-397, 181) (p=0.472) | -286 (-633, 61) (p=0.106) | -106 (-329, 117) (p=0.358) | 30 (-47, 107)  (p=0.458) |
| Maldives | 37 (-9, 84)  (p=0.118) | 27 (5, 48)  (p=0.014) | 24 (-8, 56)  (p=0.137) | 16 (-8, 39)  (p=0.19) | 0 (0, 0)  (NA) |
| Mali | 1773 (1349, 2198) (p<0.001) | 1244 (767, 1722) (p<0.001) | 809 (349, 1270) (p<0.001) | 412 (25, 798)  (p=0.037) | 296 (129, 463) (p<0.001) |
| Mauritania | 1467 (1200, 1735) (p<0.001) | 1325 (779, 1872) (p<0.001) | 353 (93, 613)  (p=0.008) | 256 (154, 358) (p<0.001) | 175 (-48, 397) (p=0.124) |
| Mozambique | 1312 (1050, 1575) (p<0.001) | 1096 (812, 1381) (p<0.001) | 533 (223, 844) (p<0.001) | 488 (249, 727) (p<0.001) | 220 (127, 313) (p<0.001) |
| Myanmar | 716 (472, 960) (p<0.001) | 213 (-434, 859) (p=0.529) | 361 (229, 494) (p<0.001) | 270 (153, 387) (p<0.001) | 87 (35, 140)  (p=0.001) |
| Nepal | 321 (236, 406) (p<0.001) | 224 (164, 284) (p<0.001) | 115 (13, 216)  (p=0.028) | 83 (53, 112)  (p<0.001) | 21 (9, 32)  (p<0.001) |
| Niger | 1180 (946, 1414) (p<0.001) | -40 (-1465, 1385) (p=0.96) | -135 (-1393, 1123) (p=0.844) | 614 (414, 814) (p<0.001) | 367 (261, 473) (p<0.001) |
| Nigeria | 1548 (1325, 1770) (p<0.001) | 1090 (750, 1429) (p<0.001) | 776 (646, 907) (p<0.001) | 330 (198, 463) (p<0.001) | 190 (131, 249) (p<0.001) |
| Pakistan | 890 (708, 1071) (p<0.001) | 598 (471, 724) (p<0.001) | 427 (-24, 878) (p=0.063) | 597 (409, 785) (p<0.001) | 81 (41, 120)  (p<0.001) |
| Rwanda | 294 (45, 543)  (p=0.021) | 203 (-47, 453) (p=0.111) | 103 (-98, 304) (p=0.321) | 17 (-178, 212) (p=0.878) | 41 (-220, 302) (p=0.772) |
| Sierra Leone | 281 (190, 372) (p<0.001) | 43 (-73, 160)  (p=0.475) | -12 (-122, 97) (p=0.837) | 79 (-155, 313) (p=0.517) | 79 (12, 147)  (p=0.022) |
| South Africa | 70 (21, 118)  (p=0.005) | 61 (-2, 125)  (p=0.059) | 81 (8, 154)  (p=0.03) | 26 (-2, 54)  (p=0.067) | 58 (-71, 186)  (p=0.384) |
| Tanzania | 368 (-43, 779) (p=0.079) | 85 (-274, 443) (p=0.656) | -386 (-917, 145) (p=0.155) | 223 (-37, 483) (p=0.093) | -86 (-339, 167) (p=0.518) |
| Timor Leste | 1760 (1238, 2283) (p<0.001) | 1022 (586, 1458) (p<0.001) | 917 (562, 1271) (p<0.001) | 434 (138, 730) (p=0.004) | 547 (115, 979) (p=0.013) |
| Togo | 272 (-751, 1295) (p=0.614) | -142 (-1565, 1280) (p=0.855) | -618 (-1579, 343) (p=0.209) | 442 (-201, 1085) (p=0.178) | 64 (-85, 213)  (p=0.407) |
| Uganda | 990 (709, 1271) (p<0.001) | 231 (-447, 910) (p=0.514) | 1090 (438, 1742) (p=0.001) | -2 (-820, 815) (p=0.996) | 518 (317, 719) (p<0.001) |
| Zambia | 495 (422, 567) (p<0.001) | 265 (207, 323) (p<0.001) | 179 (125, 233) (p<0.001) | 108 (78, 138)  (p<0.001) | 71 (56, 85)  (p<0.001) |
| Zimbabwe | 872 (655, 1088) (p<0.001) | 483 (294, 673) (p<0.001) | 665 (430, 900) (p<0.001) | 301 (170, 432) (p<0.001) | 77 (-18, 172)  (p=0.111) |
