# Supplementary material for: Association between achieving adequate antenatal care and health-seeking behaviors: A study of Demographic and Health Surveys in 47 low- and middle-income countries
Source: PLoS Med. 2024 Jul 5;21(7):e1004421. doi: 10.1371/journal.pmed.1004421 (PMC11226092; doi:10.1371/journal.pmed.1004421)
Supplement: S12 Table — (DOCX) [file pmed.1004421.s012.docx]

**S12 Table.** Postnatal care utilization rate change (per 10,000) (with 95% confidence interval and p-value) associated with achieving recommended antenatal care visits and quality.

| **Country** | **Poorest** | **Poorer** | **Middle** | **Richer** | **Richest** |
| --- | --- | --- | --- | --- | --- |
| Angola | 837 (703, 971) (p<0.001) | 478 (333, 623) (p<0.001) | 368 (290, 447) (p<0.001) | 98 (64, 131)  (p<0.001) | 73 (36, 109)  (p<0.001) |
| Bangladesh | 1850 (1656, 2043) (p<0.001) | 1609 (1424, 1794) (p<0.001) | 1064 (887, 1240) (p<0.001) | 770 (659, 881) (p<0.001) | 289 (213, 364) (p<0.001) |
| Benin | 1205 (1075, 1336) (p<0.001) | 638 (534, 743) (p<0.001) | 449 (320, 579) (p<0.001) | 262 (212, 312) (p<0.001) | 101 (73, 128)  (p<0.001) |
| Burkina Faso | 1578 (1111, 2044) (p<0.001) | 652 (190, 1113) (p=0.006) | 1186 (830, 1543) (p<0.001) | -245 (-625, 135) (p=0.207) | -357 (-557, -157) (p<0.001) |
| Burundi | 61 (-234, 357) (p=0.698) | -87 (-303, 129) (p=0.438) | -59 (-320, 201) (p=0.668) | -13 (-363, 337) (p=0.947) | 203 (-55, 462) (p=0.123) |
| Cambodia | 802 (592, 1011) (p<0.001) | 799 (624, 974) (p<0.001) | 927 (677, 1177) (p<0.001) | 489 (262, 716) (p<0.001) | 205 (60, 349)  (p=0.006) |
| Cameroon | 833 (663, 1003) (p<0.001) | 590 (473, 707) (p<0.001) | 282 (179, 385) (p<0.001) | 193 (109, 277) (p<0.001) | 54 (33, 75)  (p<0.001) |
| Chad | 1811 (1501, 2121) (p<0.001) | 1488 (1170, 1806) (p<0.001) | 1311 (949, 1673) (p<0.001) | 1193 (864, 1523) (p<0.001) | 913 (755, 1070) (p<0.001) |
| Comoros | 652 (272, 1032) (p<0.001) | 246 (-38, 529) (p=0.089) | -23 (-265, 219) (p=0.862) | 23 (-264, 310) (p=0.883) | 78 (-207, 362) (p=0.605) |
| Congo | 752 (510, 994) (p<0.001) | 329 (127, 530) (p=0.001) | 127 (-20, 274)  (p=0.09) | 24 (-2, 51)  (p=0.07) | -3 (-19, 13)  (p=0.729) |
| Congo, Democratic Republic of | 884 (571, 1196) (p<0.001) | 590 (306, 874) (p<0.001) | 570 (377, 762) (p<0.001) | 315 (125, 504) (p=0.001) | 263 (182, 343) (p<0.001) |
| Côte d'Ivoire | 1723 (1379, 2068) (p<0.001) | 988 (702, 1274) (p<0.001) | 385 (1, 770)  (p=0.049) | 185 (-8, 378)  (p=0.06) | 107 (-94, 307) (p=0.301) |
| Dominican Republic | 132 (73, 190)  (p<0.001) | 50 (-13, 113)  (p=0.12) | 39 (0, 79)  (p=0.049) | 7 (-2, 16)  (p=0.106) | 1 (-2, 5)  (p=0.47) |
| Egypt | 19 (-87, 125)  (p=0.738) | 32 (-59, 123)  (p=0.498) | 92 (-36, 220)  (p=0.159) | 61 (-2, 124)  (p=0.059) | 77 (29, 125)  (p=0.002) |
| Ethiopia | 684 (487, 882) (p<0.001) | 768 (531, 1005) (p<0.001) | 668 (460, 875) (p<0.001) | 811 (489, 1133) (p<0.001) | 320 (220, 420) (p<0.001) |
| Gabon | 524 (414, 634) (p<0.001) | 213 (89, 337)  (p<0.001) | 111 (50, 172)  (p<0.001) | 78 (19, 137)  (p=0.009) | 33 (1, 64)  (p=0.04) |
| Gambia | 151 (121, 180) (p<0.001) | 163 (99, 227)  (p<0.001) | 136 (44, 227)  (p=0.004) | -164 (-337, 9) (p=0.063) | 195 (67, 322)  (p=0.003) |
| Ghana | 298 (169, 427) (p<0.001) | 164 (41, 287)  (p=0.009) | 70 (16, 125)  (p=0.011) | 27 (5, 49)  (p=0.017) | 5 (-10, 20)  (p=0.498) |
| Guatemala | 229 (114, 345) (p<0.001) | 252 (152, 353) (p<0.001) | 114 (52, 175)  (p<0.001) | 108 (56, 160)  (p<0.001) | 25 (-14, 63)  (p=0.215) |
| Guinea | 1182 (775, 1588) (p<0.001) | 1396 (854, 1938) (p<0.001) | 196 (-204, 595) (p=0.342) | 502 (313, 690) (p<0.001) | 218 (99, 336)  (p<0.001) |
| Haiti | 1152 (987, 1317) (p<0.001) | 591 (408, 774) (p<0.001) | 529 (395, 663) (p<0.001) | 341 (225, 458) (p<0.001) | 135 (81, 189)  (p<0.001) |
| Honduras | 361 (280, 443) (p<0.001) | 172 (112, 233) (p<0.001) | 61 (37, 86)  (p<0.001) | 30 (12, 48)  (p=0.001) | 10 (2, 18)  (p=0.012) |
| India | 436 (415, 458) (p<0.001) | 276 (260, 293) (p<0.001) | 163 (150, 177) (p<0.001) | 128 (115, 141) (p<0.001) | 89 (80, 98)  (p<0.001) |
| Jordan | 88 (20, 156)  (p=0.011) | -32 (-120, 56) (p=0.485) | 40 (-17, 98)  (p=0.17) | 44 (3, 84)  (p=0.033) | 56 (10, 102)  (p=0.017) |
| Kenya | 146 (55, 237)  (p=0.002) | -67 (-188, 55) (p=0.288) | -36 (-123, 52) (p=0.434) | 28 (-34, 90)  (p=0.38) | 5 (-19, 28)  (p=0.706) |
| Lesotho | 481 (258, 704) (p<0.001) | 410 (239, 581) (p<0.001) | 339 (145, 533) (p<0.001) | 296 (127, 466) (p<0.001) | 149 (51, 248)  (p=0.003) |
| Liberia | 127 (17, 238)  (p=0.024) | -80 (-216, 55) (p=0.247) | -43 (-162, 75) (p=0.483) | 0 (-44, 44)  (p=0.997) | -44 (-146, 57) (p=0.398) |
| Madagascar | 970 (579, 1361) (p<0.001) | 832 (122, 1541) (p=0.021) | 522 (32, 1013) (p=0.036) | 1059 (553, 1566) (p<0.001) | 437 (240, 633) (p<0.001) |
| Malawi | 797 (409, 1184) (p<0.001) | 523 (164, 881) (p=0.004) | 449 (14, 884)  (p=0.043) | 241 (-228, 710) (p=0.319) | 588 (267, 909) (p<0.001) |
| Maldives | 22 (-5, 49)  (p=0.117) | 14 (-4, 31)  (p=0.118) | 6 (-13, 26)  (p=0.549) | 18 (-8, 45)  (p=0.172) | 2 (-5, 9)  (p=0.58) |
| Mali | 1114 (703, 1524) (p<0.001) | 566 (217, 915) (p=0.002) | 286 (-83, 656) (p=0.129) | 278 (58, 498)  (p=0.013) | 199 (63, 334)  (p=0.004) |
| Mauritania | 918 (546, 1290) (p<0.001) | 577 (239, 914) (p<0.001) | 448 (253, 643) (p<0.001) | 374 (221, 527) (p<0.001) | 145 (21, 268)  (p=0.021) |
| Myanmar | 1331 (1036, 1627) (p<0.001) | 831 (466, 1196) (p<0.001) | 470 (92, 847)  (p=0.015) | 441 (270, 612) (p<0.001) | 84 (-41, 208)  (p=0.188) |
| Nepal | 573 (318, 829) (p<0.001) | -93 (-413, 227) (p=0.582) | 28 (-214, 271) (p=0.831) | -163 (-368, 43) (p=0.12) | -46 (-152, 61) (p=0.408) |
| Niger | 1043 (113, 1972) (p=0.028) | 1015 (179, 1851) (p=0.017) | 1009 (206, 1812) (p=0.014) | 956 (377, 1534) (p=0.001) | 338 (184, 493) (p<0.001) |
| Nigeria | 1057 (909, 1205) (p<0.001) | 750 (616, 885) (p<0.001) | 531 (432, 629) (p<0.001) | 304 (253, 355) (p<0.001) | 130 (81, 179)  (p<0.001) |
| Pakistan | 352 (54, 649)  (p=0.02) | 439 (95, 783)  (p=0.012) | 145 (-5, 294)  (p=0.057) | 106 (-11, 223) (p=0.075) | 159 (27, 291)  (p=0.018) |
| Rwanda | 594 (408, 781) (p<0.001) | 448 (276, 620) (p<0.001) | 191 (-116, 499) (p=0.224) | 281 (57, 504)  (p=0.014) | 43 (-167, 253) (p=0.701) |
| Sierra Leone | 54 (-49, 158)  (p=0.31) | 22 (-124, 168) (p=0.781) | 41 (-33, 115)  (p=0.281) | -36 (-109, 37) (p=0.339) | 56 (-6, 118)  (p=0.075) |
| South Africa | 116 (63, 168)  (p<0.001) | 179 (72, 287)  (p=0.001) | 61 (19, 103)  (p=0.005) | 33 (-42, 108)  (p=0.396) | 50 (-3, 104)  (p=0.066) |
| Tanzania | -540 (-945, -136) (p=0.009) | -729 (-1159, -298) (p<0.001) | -805 (-1279, -331) (p<0.001) | -839 (-1214, -465) (p<0.001) | -889 (-1175, -604) (p<0.001) |
| Timor Leste | 350 (89, 611)  (p=0.009) | 190 (-55, 435) (p=0.128) | -91 (-298, 117) (p=0.398) | -205 (-434, 23) (p=0.078) | -177 (-430, 77) (p=0.174) |
| Togo | 906 (160, 1653) (p=0.017) | 880 (112, 1648) (p=0.024) | 292 (-182, 765) (p=0.23) | -12 (-307, 282) (p=0.94) | -30 (-204, 143) (p=0.744) |
| Uganda | 162 (-200, 524) (p=0.387) | 268 (-216, 753) (p=0.281) | 696 (339, 1054) (p<0.001) | 439 (67, 810)  (p=0.021) | 596 (310, 882) (p<0.001) |
| Zambia | 657 (531, 783) (p<0.001) | 327 (185, 470) (p<0.001) | 98 (-79, 275)  (p=0.28) | -192 (-371, -13) (p=0.035) | -155 (-239, -71) (p<0.001) |
| Zimbabwe | 1207 (1050, 1365) (p<0.001) | 916 (767, 1064) (p<0.001) | 862 (721, 1002) (p<0.001) | 481 (382, 580) (p<0.001) | 246 (189, 302) (p<0.001) |
