# Supplementary material for: Association between achieving adequate antenatal care and health-seeking behaviors: A study of Demographic and Health Surveys in 47 low- and middle-income countries
Source: PLoS Med. 2024 Jul 5;21(7):e1004421. doi: 10.1371/journal.pmed.1004421 (PMC11226092; doi:10.1371/journal.pmed.1004421)
Supplement: S13 Table — (DOCX) [file pmed.1004421.s013.docx]

**S13 Table.** Stunting rate change (per 10,000) (with 95% confidence interval and p-value) associated with recommended antenatal care visits and quality.

| **Country** | **Poorest** | **Poorer** | **Middle** | **Richer** | **Richest** |
| --- | --- | --- | --- | --- | --- |
| Bangladesh | -672 (-808, -535) (p<0.001) | -554 (-675, -433) (p<0.001) | -473 (-568, -378) (p<0.001) | -335 (-414, -255) (p<0.001) | -159 (-197, -121) (p<0.001) |
| Benin | -124 (-178, -71) (p<0.001) | -81 (-116, -46) (p<0.001) | -65 (-93, -37)  (p<0.001) | -36 (-52, -21)  (p<0.001) | -18 (-27, -9)  (p<0.001) |
| Burkina Faso | -427 (-793, -60) (p=0.022) | -747 (-1483, -10) (p=0.047) | 90 (-587, 768) (p=0.806) | -342 (-629, -56) (p=0.019) | -146 (-272, -21) (p=0.022) |
| Burundi | -123 (-195, -51) (p<0.001) | 671 (67, 1276) (p=0.029) | -275 (-451, -99) (p=0.002) | -354 (-868, 160) (p=0.178) | -485 (-743, -226) (p<0.001) |
| Cambodia | -502 (-649, -356) (p<0.001) | -374 (-498, -249) (p<0.001) | -356 (-474, -237) (p<0.001) | -218 (-434, -1) (p=0.048) | -142 (-194, -90) (p<0.001) |
| Cameroon | -110 (-211, -9) (p=0.033) | -41 (-200, 117) (p=0.623) | -116 (-179, -53) (p<0.001) | -52 (-112, 9)  (p=0.095) | -8 (-39, 24)  (p=0.65) |
| Chad | -636 (-823, -448) (p<0.001) | -527 (-925, -130) (p=0.009) | -359 (-598, -120) (p=0.003) | -294 (-660, 73) (p=0.116) | -157 (-331, 17) (p=0.076) |
| Comoros | -102 (-150, -55) (p<0.001) | -275 (-475, -75) (p=0.007) | -34 (-52, -15)  (p<0.001) | -28 (-143, 86) (p=0.639) | -8 (-76, 61)  (p=0.837) |
| Congo | -122 (-176, -68) (p<0.001) | -81 (-188, 27) (p=0.141) | -89 (-146, -33) (p=0.002) | -5 (-8, -2)  (p=0.004) | -11 (-24, 2)  (p=0.098) |
| Congo, Democratic Republic of | -160 (-236, -84) (p<0.001) | -107 (-362, 149) (p=0.42) | 135 (-258, 529) (p=0.511) | 95 (-78, 268)  (p=0.286) | -137 (-246, -27) (p=0.014) |
| Côte d'Ivoire | -121 (-177, -66) (p<0.001) | -24 (-192, 144) (p=0.794) | -412 (-695, -129) (p=0.004) | -228 (-453, -3) (p=0.047) | -48 (-77, -18)  (p=0.002) |
| Dominican Republic | -36 (-48, -23)  (p<0.001) | -13 (-22, -5)  (p=0.002) | -4 (-13, 6)  (p=0.452) | -4 (-9, 1)  (p=0.12) | -3 (-9, 2)  (p=0.241) |
| Egypt | -73 (-154, 8)  (p=0.077) | 12 (-67, 91)  (p=0.78) | 14 (-35, 62)  (p=0.595) | 33 (-51, 117)  (p=0.447) | 60 (38, 82)  (p<0.001) |
| Ethiopia | -534 (-772, -295) (p<0.001) | -277 (-596, 42) (p=0.088) | -62 (-475, 350) (p=0.781) | -182 (-529, 164) (p=0.306) | -228 (-327, -129) (p<0.001) |
| Gabon | -55 (-81, -29)  (p<0.001) | -36 (-71, -1)  (p=0.046) | -38 (-96, 21)  (p=0.212) | -3 (-64, 57)  (p=0.926) | -1 (-2, 0)  (p=0.037) |
| Gambia | -12 (-19, -6)  (p<0.001) | -26 (-50, -2)  (p=0.037) | -1 (-23, 22)  (p=0.956) | -8 (-13, -4)  (p<0.001) | -6 (-10, -2)  (p=0.002) |
| Ghana | -25 (-37, -13)  (p<0.001) | 5 (-33, 42)  (p=0.824) | -15 (-33, 3)  (p=0.094) | -1 (-4, 1)  (p=0.301) | -2 (-6, 2)  (p=0.304) |
| Guatemala | -366 (-542, -190) (p<0.001) | -395 (-520, -270) (p<0.001) | -245 (-393, -97) (p=0.001) | -131 (-234, -28) (p=0.013) | -45 (-78, -13)  (p=0.007) |
| Guinea | -413 (-715, -110) (p=0.008) | 247 (-80, 575) (p=0.139) | -290 (-507, -73) (p=0.009) | -105 (-213, 2) (p=0.055) | -78 (-128, -28) (p=0.002) |
| Haiti | -346 (-475, -218) (p<0.001) | -267 (-363, -172) (p<0.001) | -135 (-247, -23) (p=0.018) | -78 (-115, -41) (p<0.001) | -42 (-60, -23)  (p<0.001) |
| Honduras | -135 (-167, -103) (p<0.001) | -162 (-244, -80) (p<0.001) | -65 (-135, 5)  (p=0.07) | -57 (-116, 3)  (p=0.062) | -27 (-42, -13)  (p<0.001) |
| India | -47 (-61, -33)  (p<0.001) | -22 (-30, -13)  (p<0.001) | -18 (-24, -12)  (p<0.001) | -2 (-11, 8)  (p=0.726) | 3 (-4, 10)  (p=0.464) |
| Kenya | -151 (-223, -80) (p<0.001) | -96 (-140, -52) (p<0.001) | -107 (-152, -62) (p<0.001) | -76 (-106, -46) (p<0.001) | -15 (-24, -6)  (p=0.002) |
| Lesotho | -30 (-45, -15)  (p<0.001) | -53 (-404, 298) (p=0.78) | 49 (-253, 351) (p=0.763) | -51 (-94, -7)  (p=0.023) | -46 (-82, -11)  (p=0.01) |
| Liberia | -69 (-97, -41)  (p<0.001) | 5 (-103, 113)  (p=0.933) | -44 (-65, -22)  (p<0.001) | -30 (-49, -11)  (p=0.002) | -12 (-21, -4)  (p=0.003) |
| Madagascar | 153 (-226, 531) (p=0.437) | -279 (-727, 168) (p=0.223) | 207 (-60, 474) (p=0.128) | 122 (-133, 378) (p=0.353) | 114 (-121, 348) (p=0.347) |
| Malawi | 42 (-155, 239)  (p=0.69) | 42 (-159, 244) (p=0.694) | -139 (-400, 122) (p=0.302) | -93 (-263, 78) (p=0.289) | 572 (235, 909) (p<0.001) |
| Maldives | -17 (-27, -8)  (p<0.001) | -9 (-15, -4)  (p=0.001) | -5 (-14, 3)  (p=0.23) | -8 (-23, 7)  (p=0.307) | 1 (-5, 7)  (p=0.771) |
| Mali | -66 (-483, 350) (p=0.768) | -319 (-739, 101) (p=0.136) | -551 (-875, -227) (p<0.001) | -422 (-610, -235) (p<0.001) | -56 (-169, 56)  (p=0.33) |
| Mauritania | -203 (-534, 129) (p=0.233) | -642 (-1040, -244) (p=0.002) | -599 (-839, -359) (p<0.001) | -60 (-183, 64) (p=0.349) | -28 (-92, 36)  (p=0.394) |
| Mozambique | -140 (-207, -74) (p<0.001) | 59 (-309, 428) (p=0.765) | 156 (-204, 517) (p=0.403) | -81 (-121, -40) (p<0.001) | -64 (-256, 128) (p=0.526) |
| Myanmar | -447 (-566, -327) (p<0.001) | 37 (-398, 472) (p=0.877) | -236 (-347, -126) (p<0.001) | -52 (-177, 72)  (p=0.42) | -73 (-131, -14) (p=0.015) |
| Nepal | -703 (-882, -524) (p<0.001) | -720 (-1006, -434) (p<0.001) | -457 (-668, -247) (p<0.001) | -250 (-337, -163) (p<0.001) | -93 (-127, -59) (p<0.001) |
| Niger | -101 (-314, 111) (p=0.355) | -697 (-1456, 62) (p=0.071) | -88 (-285, 109) (p=0.387) | 391 (15, 767)  (p=0.041) | 38 (-248, 323) (p=0.807) |
| Nigeria | -179 (-338, -21) (p=0.026) | -328 (-446, -210) (p<0.001) | -117 (-201, -32) (p=0.007) | 25 (-39, 90)  (p=0.449) | -11 (-23, 0)  (p=0.056) |
| Pakistan | -256 (-478, -33) (p=0.024) | -408 (-630, -186) (p<0.001) | -235 (-357, -113) (p<0.001) | -219 (-328, -111) (p<0.001) | -32 (-65, 0)  (p=0.051) |
| Rwanda | -187 (-399, 25) (p=0.083) | -176 (-381, 30) (p=0.093) | -438 (-652, -224) (p<0.001) | -529 (-804, -254) (p<0.001) | -330 (-527, -134) (p=0.001) |
| Sierra Leone | -43 (-75, -11)  (p=0.009) | -39 (-67, -11)  (p=0.006) | -20 (-50, 11)  (p=0.207) | -27 (-54, -1)  (p=0.045) | -28 (-43, -13)  (p<0.001) |
| South Africa | -11 (-19, -2)  (p=0.012) | -54 (-190, 81)  (p=0.44) | -17 (-38, 5)  (p=0.127) | -8 (-110, 93)  (p=0.884) | -33 (-101, 34) (p=0.337) |
| Tanzania | 471 (263, 678) (p<0.001) | 126 (-308, 560) (p=0.581) | 26 (-244, 296) (p=0.861) | 363 (210, 516) (p<0.001) | 80 (-61, 222)  (p=0.269) |
| Timor Leste | 140 (-125, 405) (p=0.304) | -358 (-785, 68)  (p=0.1) | 578 (238, 918) (p<0.001) | -25 (-271, 220) (p=0.85) | 121 (-42, 285) (p=0.147) |
| Togo | 545 (-324, 1414) (p=0.221) | -51 (-992, 891) (p=0.923) | 108 (-392, 609) (p=0.684) | -187 (-335, -38) (p=0.013) | 28 (-17, 72)  (p=0.229) |
| Uganda | -119 (-193, -45) (p=0.002) | -103 (-169, -37) (p=0.002) | -598 (-1034, -162) (p=0.007) | -83 (-136, -31) (p=0.002) | -162 (-268, -57) (p=0.003) |
| Zambia | -67 (-126, -8)  (p=0.026) | 119 (-26, 265) (p=0.108) | -41 (-85, 3)  (p=0.065) | -26 (-171, 119) (p=0.739) | -116 (-200, -32) (p=0.007) |
| Zimbabwe | -132 (-186, -78) (p<0.001) | -146 (-245, -46) (p=0.004) | 50 (-81, 180)  (p=0.463) | -85 (-119, -52) (p<0.001) | -34 (-63, -5)  (p=0.021) |
