# Supplementary material for: Association between achieving adequate antenatal care and health-seeking behaviors: A study of Demographic and Health Surveys in 47 low- and middle-income countries
Source: PLoS Med. 2024 Jul 5;21(7):e1004421. doi: 10.1371/journal.pmed.1004421 (PMC11226092; doi:10.1371/journal.pmed.1004421)
Supplement: S14 Table — (DOCX) [file pmed.1004421.s014.docx]

**S14 Table.** Wasting rate change (per 10,000) (with 95% confidence interval) and p-value associated with recommended antenatal care visits and quality.

| **Country** | **Poorest** | **Poorer** | **Middle** | **Richer** | **Richest** |
| --- | --- | --- | --- | --- | --- |
| Bangladesh | -5 (-59, 50)  (p=0.873) | -3 (-47, 40)  (p=0.889) | -3 (-37, 32)  (p=0.896) | -2 (-26, 23)  (p=0.912) | 0 (-12, 12)  (p=0.997) |
| Benin | -38 (-65, -11)  (p=0.006) | -23 (-40, -7)  (p=0.006) | -19 (-32, -7)  (p=0.003) | -11 (-18, -4)  (p=0.002) | -6 (-9, -2)  (p<0.001) |
| Burkina Faso | -71 (-142, 0)  (p=0.051) | -66 (-133, 1)  (p=0.052) | 231 (-251, 713) (p=0.353) | -413 (-923, 97) (p=0.112) | -86 (-273, 101) (p=0.374) |
| Burundi | 127 (-40, 295) (p=0.136) | 180 (-88, 449) (p=0.189) | 103 (-37, 242)  (p=0.15) | 93 (-35, 221)  (p=0.156) | -5 (-117, 107) (p=0.934) |
| Cambodia | -10 (-39, 18)  (p=0.493) | -11 (-35, 14)  (p=0.395) | -10 (-30, 10)  (p=0.343) | -10 (-26, 7)  (p=0.265) | -6 (-17, 4)  (p=0.252) |
| Cameroon | -213 (-337, -89) (p<0.001) | -104 (-166, -42) (p=0.001) | -88 (-123, -53) (p<0.001) | -13 (-23, -3)  (p=0.014) | -4 (-7, -1)  (p=0.015) |
| Chad | -148 (-358, 62) (p=0.169) | -276 (-524, -29) (p=0.028) | -424 (-661, -188) (p<0.001) | -604 (-915, -293) (p<0.001) | 64 (-18, 145)  (p=0.127) |
| Comoros | 13 (-57, 83)  (p=0.732) | 81 (-95, 257)  (p=0.373) | 25 (-12, 62)  (p=0.186) | -1 (-33, 31)  (p=0.958) | -3 (-20, 14)  (p=0.744) |
| Congo | -13 (-28, 3)  (p=0.105) | -9 (-17, -1)  (p=0.033) | -7 (-12, -3)  (p=0.001) | -2 (-3, -1)  (p=0.008) | 24 (0, 48)  (p=0.053) |
| Congo, Democratic Republic of | -147 (-285, -8) (p=0.038) | -118 (-241, 4) (p=0.057) | -116 (-236, 4) (p=0.058) | -74 (-149, 0)  (p=0.049) | -85 (-141, -28) (p=0.003) |
| Côte d'Ivoire | -43 (-175, 89) (p=0.532) | -35 (-141, 71) (p=0.527) | 24 (-91, 139)  (p=0.692) | 31 (-81, 142)  (p=0.602) | 3 (-30, 36)  (p=0.872) |
| Dominican Republic | 0 (-1, 1)  (p=0.91) | 0 (0, 0)  (p=0.988) | 0 (0, 0)  (p=0.996) | 0 (0, 0)  (p=0.973) | 0 (0, 0)  (p=0.823) |
| Egypt | -7 (-19, 5)  (p=0.243) | -6 (-15, 4)  (p=0.243) | -5 (-12, 3)  (p=0.213) | -4 (-9, 2)  (p=0.215) | -2 (-5, 1)  (p=0.214) |
| Ethiopia | -199 (-323, -74) (p=0.002) | -147 (-241, -53) (p=0.002) | 83 (-170, 335)  (p=0.53) | -101 (-170, -33) (p=0.004) | -72 (-178, 35) (p=0.187) |
| Gabon | -8 (-13, -2)  (p=0.004) | -3 (-5, -1)  (p<0.001) | -2 (-3, 0)  (p=0.014) | -1 (-3, 0)  (p=0.026) | 2 (-2, 6)  (p=0.346) |
| Gambia | -5 (-7, -2)  (p<0.001) | 26 (7, 44)  (p=0.006) | 10 (-42, 63)  (p=0.714) | -1 (-7, 6)  (p=0.862) | -4 (-5, -2)  (p<0.001) |
| Ghana | -6 (-11, -2)  (p=0.01) | -5 (-8, -2)  (p=0.003) | -11 (-21, -2)  (p=0.018) | 3 (-5, 11)  (p=0.47) | 0 (-1, 0)  (p=0.14) |
| Guatemala | -1 (-3, 1)  (p=0.371) | -1 (-2, 1  ) (p=0.443) | 0 (-1, 1)  (p=0.448) | 0 (-1, 0)  (p=0.491) | 0 (0, 0)  (p=0.498) |
| Guinea | -34 (-71, 3)  (p=0.071) | -100 (-245, 45) (p=0.178) | -104 (-225, 17) (p=0.093) | -15 (-28, -2)  (p=0.025) | -8 (-12, -3)  (p<0.001) |
| Haiti | -1 (-10, 8)  (p=0.798) | -1 (-7, 5)  (p=0.833) | 0 (-4, 3)  (p=0.899) | 0 (-3, 2)  (p=0.921) | 0 (-1, 1)  (p=0.935) |
| Honduras | -1 (-2, 1)  (p=0.49) | 0 (-1, 1)  (p=0.617) | 0 (-1, 0)  (p=0.681) | 0 (0, 0)  (p=0.843) | 0 (0, 0)  (p=0.735) |
| India | 5 (-6, 16)  (p=0.401) | 4 (-2, 11)  (p=0.213) | 4 (-1, 9)  (p=0.128) | 3 (0, 7)  (p=0.082) | 3 (0, 5)  (p=0.052) |
| Kenya | -21 (-37, -5)  (p=0.01) | -8 (-12, -3)  (p<0.001) | -10 (-15, -5)  (p<0.001) | -9 (-12, -5)  (p<0.001) | -4 (-6, -2)  (p<0.001) |
| Lesotho | -1 (-5, 2)  (p=0.399) | 6 (-11, 24)  (p=0.483) | -1 (-4, 1)  (p=0.317) | -2 (-4, 0)  (p=0.033) | -3 (-7, 0)  (p=0.036) |
| Liberia | -4 (-9, 2)  (p=0.211) | -9 (-39, 22)  (p=0.591) | -2 (-5, 2)  (p=0.296) | 8 (-18, 34)  (p=0.558) | 9 (-6, 24)  (p=0.234) |
| Madagascar | 4 (-32, 39)  (p=0.847) | -174 (-356, 8) (p=0.061) | -136 (-258, -15) (p=0.027) | 8 (-6, 22)  (p=0.25) | 33 (-38, 103)  (p=0.373) |
| Malawi | 2 (-6, 11)  (p=0.609) | 3 (-5, 12)  (p=0.445) | 13 (-100, 126) (p=0.832) | 1 (-5, 8)  (p=0.693) | 1 (-4, 6)  (p=0.639) |
| Maldives | 0 (-2, 1)  (p=0.91) | 0 (-1, 1)  (p=0.903) | 0 (-1, 1)  (p=0.959) | 0 (-1, 1)  (p=0.944) | 0 (-1, 0)  (p=0.475) |
| Mali | -303 (-482, -124) (p<0.001) | -254 (-411, -97) (p=0.002) | -329 (-513, -145) (p<0.001) | -217 (-335, -98) (p<0.001) | -50 (-76, -23)  (p<0.001) |
| Mauritania | -26 (-50, -1)  (p=0.039) | 25 (-102, 152) (p=0.713) | 8 (-97, 114)  (p=0.888) | -79 (-202, 45) (p=0.213) | -23 (-33, -13)  (p<0.001) |
| Mozambique | -3 (-29, 23)  (p=0.828) | 1 (-22, 25)  (p=0.91) | -76 (-241, 88) (p=0.369) | -33 (-143, 77) (p=0.567) | 0 (-3, 3)  (p=0.978) |
| Myanmar | -6 (-27, 15)  (p=0.611) | -4 (-20, 11)  (p=0.581) | -4 (-16, 8)  (p=0.513) | -3 (-10, 4)  (p=0.456) | -2 (-5, 2)  (p=0.342) |
| Nepal | -11 (-49, 28)  (p=0.602) | -8 (-38, 21)  (p=0.587) | -6 (-27, 16)  (p=0.615) | -3 (-16, 9)  (p=0.591) | -1 (-6, 3)  (p=0.632) |
| Niger | 36 (-33, 105)  (p=0.307) | 137 (-84, 358) (p=0.225) | 562 (-318, 1442) (p=0.212) | 138 (-30, 307) (p=0.107) | -96 (-234, 42) (p=0.173) |
| Nigeria | -63 (-133, 6)  (p=0.073) | 0 (-113, 113)  (p=0.997) | -22 (-50, 6)  (p=0.117) | -12 (-26, 1)  (p=0.072) | 1 (-12, 14)  (p=0.903) |
| Pakistan | -7 (-34, 20)  (p=0.634) | -5 (-24, 14)  (p=0.622) | -3 (-15, 9)  (p=0.67) | -1 (-9, 6)  (p=0.741) | -1 (-4, 2)  (p=0.561) |
| Rwanda | -3 (-7, 1)  (p=0.101) | -2 (-6, 1)  (p=0.157) | -2 (-5, 1)  (p=0.224) | -43 (-70, -16)  (p=0.002) | -3 (-4, -1)  (p=0.001) |
| Sierra Leone | 4 (-3, 12)  (p=0.258) | 15 (-54, 84)  (p=0.682) | 2 (-2, 7)  (p=0.349) | 47 (16, 78)  (p=0.003) | 4 (-12, 19)  (p=0.66) |
| South Africa | -3 (-5, -1)  (p=0.002) | -2 (-4, -1)  (p=0.008) | -2 (-3, -1)  (p=0.003) | -1 (-3, 0)  (p=0.038) | -2 (-4, 0)  (p=0.075) |
| Tanzania | 151 (-5, 306)  (p=0.057) | 66 (-129, 262) (p=0.516) | 147 (-47, 342) (p=0.138) | 104 (-15, 222) (p=0.086) | 137 (40, 235)  (p=0.006) |
| Timor Leste | -23 (-79, 33)  (p=0.435) | -20 (-70, 30)  (p=0.434) | -22 (-67, 23)  (p=0.349) | -19 (-55, 16)  (p=0.283) | -17 (-45, 12)  (p=0.25) |
| Togo | -45 (-88, -1)  (p=0.043) | -323 (-617, -29) (p=0.031) | -29 (-56, -2)  (p=0.034) | -14 (-25, -2)  (p=0.019) | -7 (-11, -3)  (p<0.001) |
| Uganda | 37 (-20, 93)  (p=0.203) | 32 (-17, 81)  (p=0.202) | 33 (-23, 89)  (p=0.254) | -89 (-133, -45) (p<0.001) | 11 (-7, 29)  (p=0.232) |
| Zambia | 2 (-10, 13)  (p=0.809) | 1 (-8, 10)  (p=0.833) | -26 (-49, -3)  (p=0.025) | 31 (3, 59)  (p=0.029) | 2 (-53, 57)  (p=0.947) |
| Zimbabwe | 1 (-8, 9)  (p=0.878) | 20 (-31, 70)  (p=0.454) | -15 (-33, 3)  (p=0.094) | -2 (-6, 1)  (p=0.225) | 0 (-1, 1)  (p=0.581) |
