# Supplementary figures and images for: Association between achieving adequate antenatal care and health-seeking behaviors: A study of Demographic and Health Surveys in 47 low- and middle-income countries
Source: PLoS Med. 2024 Jul 5;21(7):e1004421. doi: 10.1371/journal.pmed.1004421 (PMC11226092; doi:10.1371/journal.pmed.1004421)

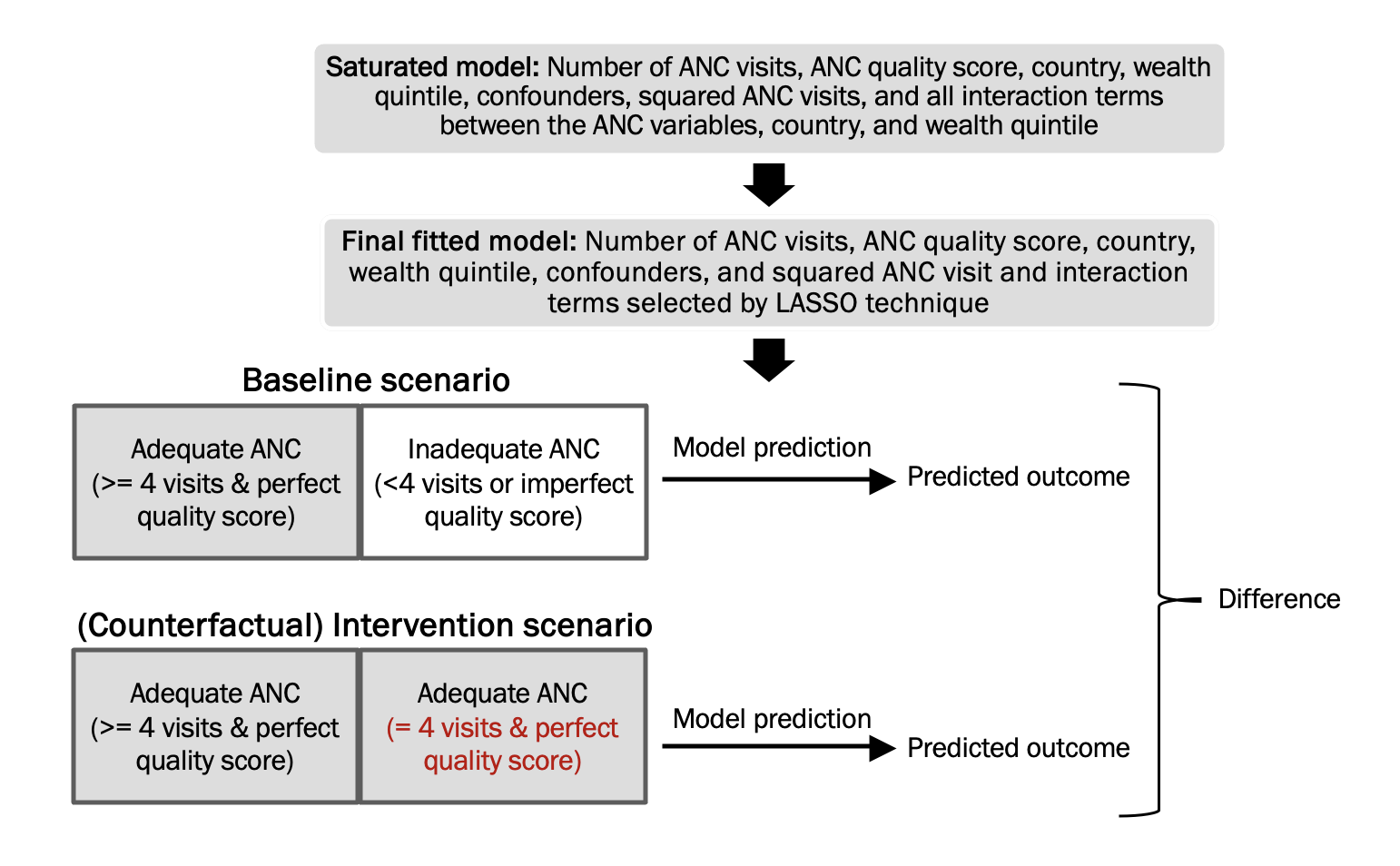


**S1 Figure.** The process of statistical analysis.

Supplement: S1 Fig — (DOCX) [file pmed.1004421.s015.docx]
