# Supplementary material for: Association between achieving adequate antenatal care and health-seeking behaviors: A study of Demographic and Health Surveys in 47 low- and middle-income countries
Source: PLoS Med. 2024 Jul 5;21(7):e1004421. doi: 10.1371/journal.pmed.1004421 (PMC11226092; doi:10.1371/journal.pmed.1004421)
Supplement: S3 Fig — (DOCX) [file pmed.1004421.s017.docx]

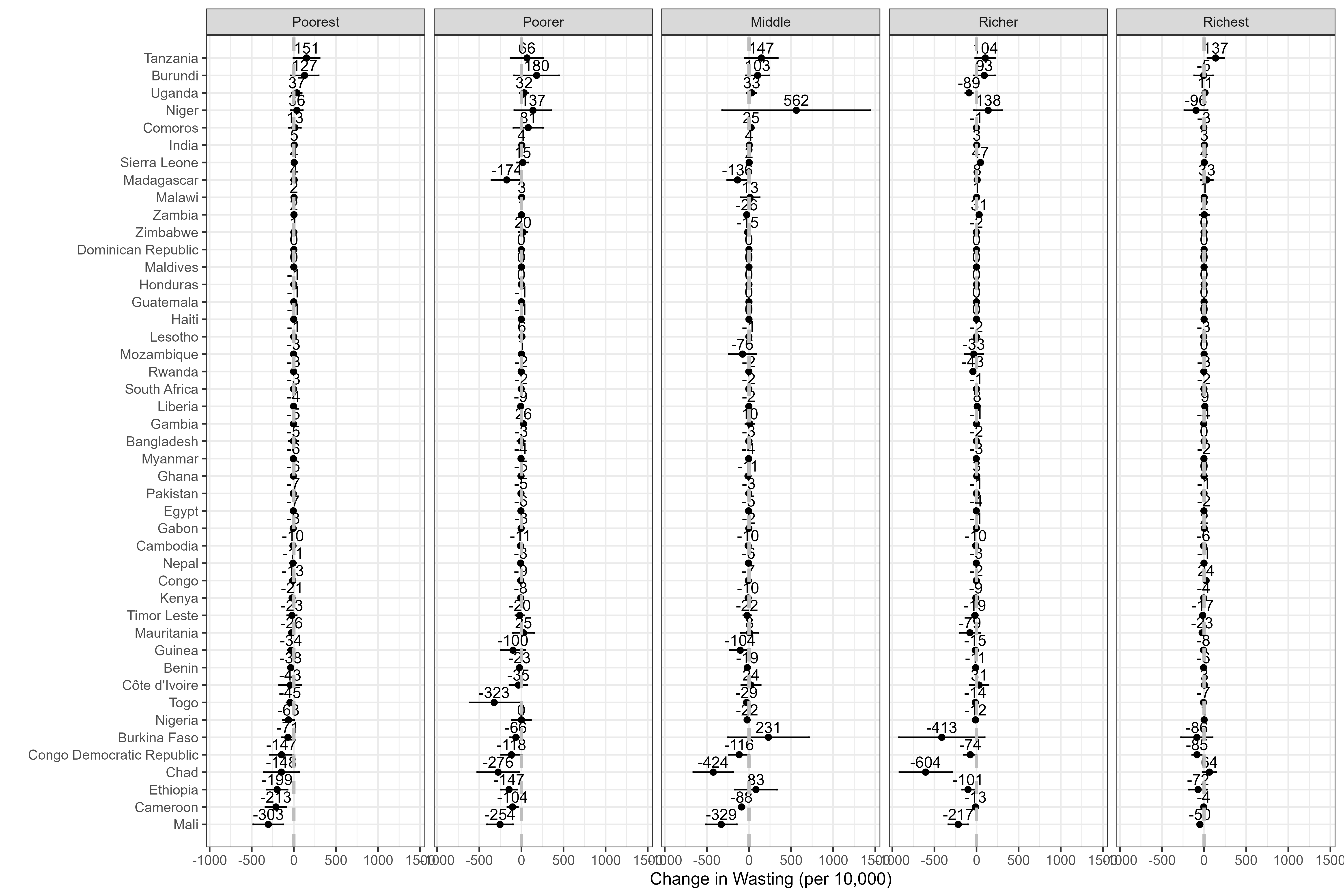


**S3 Figure**. Wasting rate change associated with recommended antenatal care visits and quality across five wealth quintiles and countries. This change, represented by a line in the figure, is measured by the predicted difference in wasting rate between two scenarios: an intervention that ensures all women achieve the adequate level of ANC utilization and quality, and a baseline scenario represents the current state of ANC in each country.
