# Supplementary material for: Association between achieving adequate antenatal care and health-seeking behaviors: A study of Demographic and Health Surveys in 47 low- and middle-income countries
Source: PLoS Med. 2024 Jul 5;21(7):e1004421. doi: 10.1371/journal.pmed.1004421 (PMC11226092; doi:10.1371/journal.pmed.1004421)
Supplement: S1 STROBE Checklist — (DOCX) [file pmed.1004421.s018.docx]

**Supplementary webappendix**

**Content**

**Table S1.** Countries included and corresponding Demographic and Health Surveys (DHS), along with gross national income (GNI) estimates.

**Table S2**. Distribution of demographic and health variables (number and proportion of participants) for the sample with missing values on antenatal care utilization or quality (not included in the analysis).

**Table S3**. Baseline unweighted absolute facility birth rates (per 10,000) across wealth quintiles and countries.

**Table S4**. Baseline unweighted absolute diphtheria-pertussis-tetanus 3rd dose (DPT3) vaccination rate (per 10,000) across wealth quintiles and countries.

**Table S5**. Baseline unweighted absolute measles vaccination rates (per 10,000) across wealth quintiles and countries.

**Table S6**. Baseline unweighted absolute postnatal care utilization rates (per 10,000) across wealth quintiles and countries.

**Table S7**. Baseline unweighted absolute stunting rates (per 10,000) across wealth quintiles and countries.

**Table S8**. Baseline unweighted absolute wasting rates (per 10,000) across wealth quintiles and countries.

**Table S9.** Facility birth rate change (per 10,000) (with 95% confidence interval and p value) associated with achieving recommended antenatal care visits and quality.

**Table S10.** Diphtheria-pertussis-tetanus 3rd dose (DPT3) vaccination rate change (per 10,000) (with 95% confidence interval and p value) associated with achieving recommended antenatal care visits and quality.

**Table S11.** Measles vaccination rate change (per 10,000) (with 95% confidence interval and p value) associated with achieving recommended antenatal care visits and quality.

**Table S12.** Postnatal care utilization rate change (per 10,000) (with 95% confidence interval and p value) associated with achieving recommended antenatal care visits and quality.

**Table S13.** Stunting rate change (per 10,000) (with 95% confidence interval and p value) associated with recommended antenatal care visits and quality.

**Table S14.** Wasting rate change (per 10,000) (with 95% confidence interval) and p value associated with recommended antenatal care visits and quality.

**Figure S1**. The statistical analysis process.

**Figure S2**. Stunting rate change associated with recommended antenatal care visits and quality across five wealth quintiles and countries.

**Figure S3**. Wasting rate change associated with recommended antenatal care visits and quality across five wealth quintiles and countries.

**Checklist S1.** STROBE Statement—checklist of items that should be included in reports of observational studies.

**Table S1.** Countries included and corresponding Demographic and Health Surveys (DHS), along with gross national income (GNI) estimates.

| **Country** | **Standard DHS survey** | **GNI (2022 USD)*** |
| --- | --- | --- |
| Angola | 2015–2016 | 1,900 |
| Bangladesh | 2011; 2014; 2017–2018 | 2,820 |
| Benin | 2011–2012; 2017–2018 | 1,400 |
| Burkina Faso | 2010 | 840 |
| Burundi | 2010; 2016–2017 | 240 |
| Cambodia | 2010; 2014; 2021–2022 | 1,700 |
| Cameroon | 2011; 2018 | 1,660 |
| Chad | 2014–2015 | 690 |
| Comoros | 2012 | 1,610 |
| Congo | 2011–2012 | 2,060 |
| Congo Democratic Republic | 2013–2014; 2017 | 590 |
| Cote d'Ivoire | 2011–2012 | 2,620 |
| Dominican Republic | 2013 | 9,050 |
| Egypt | 2014 | 4,100 |
| Ethiopia | 2011; 2016; 2019 (interim) | 1,020 |
| Gabon | 2012; 2019–2021 | 7,540 |
| Gambia | 2013; 2019–2020 | 810 |
| Ghana | 2014 | 2,350 |
| Guatemala | 2014–2015 | 5,350 |
| Guinea | 2012; 2018 | 1,180 |
| Haiti | 2012; 2016–2017 | 1,610 |
| Honduras | 2011–2012 | 2,740 |
| India | 2015–2016; 2019–2021 | 2,380 |
| Jordan | 2012; 2017–2018 | 4,260 |
| Kenya | 2014; 2022 | 2,170 |
| Lesotho | 2014 | 1,260 |
| Liberia | 2013; 2019–2020 | 680 |
| Madagascar | 2021 | 510 |
| Malawi | 2010; 2015–2016 | 640 |
| Maldives | 2016–2017 | 11,030 |
| Mali | 2012–2013; 2018 | 850 |
| Mauritania | 2019–2021 | 2,160 |
| Mozambique | 2011 | 500 |
| Myanmar | 2015–2016 | 1,210 |
| Nepal | 2011; 2016; 2022 | 1,340 |
| Niger | 2012 | 610 |
| Nigeria | 2013; 2018 | 2,140 |
| Pakistan | 2012–2013; 2017–2018 | 1,580 |
| Rwanda | 2010; 2014–2015; 2019–2020 | 930 |
| Sierra Leone | 2013; 2019 | 510 |
| South Africa | 2016 | 6,780 |
| Tanzania | 2010; 2015–2016 | 1,200 |
| Timor-Leste | 2009–2010; 2016 | 1,970 |
| Togo | 2013–2014 | 990 |
| Uganda | 2011; 2016 | 930 |
| Zambia | 2013–2014; 2018 | 1,170 |
| Zimbabwe | 2010–2011; 2015 | 1,500 |

*Source: The World Bank.

**Table S2**. Distribution of demographic and health variables (presented as n (%), number and proportion of participants) for the sample with missing values on antenatal care utilization or quality (not included in the analysis).

|  | **Poorest** | **Poorer** | **Middle** | **Richer** | **Richest** | **Total** |
| --- | --- | --- | --- | --- | --- | --- |
| **Age at childbirth** |  |  |  |  |  |  |
| <25 years | 1,263 (20.3%) | 1,078 (21.1%) | 740 (20.7%) | 601 (19.4%) | 359 (14.6%) | 4,041 (19.7%) |
| 25–34 years | 3,206 (51.4%) | 2,820 (55.2%) | 2,071 (57.8%) | 1,860 (60.1%) | 1,562 (63.6%) | 11,519 (56.3%) |
| >34 years | 1,767 (28.3%) | 1,209 (23.7%) | 772 (21.5%) | 635 (20.5%) | 534 (21.8%) | 4,917 (24.0%) |
| **Education** |  |  |  |  |  |  |
| No education | 12,664 (85.5%) | 8,689 (73.5%) | 5,363 (58.1%) | 3,011 (41.3%) | 1,116 (19.4%) | 30,843 (63.1%) |
| Primacy | 1,481 (10.0%) | 1,804 (15.3%) | 1,731 (18.8%) | 1,555 (21.3%) | 734 (12.7%) | 7,305 (14.9%) |
| Secondary | 568 (3.8%) | 1,063 (9.0%) | 1,588 (17.2%) | 1,851 (25.4%) | 2,002 (34.7%) | 7,072 (14.5%) |
| Higher | 94 (0.6%) | 263 (2.2%) | 545 (5.9%) | 867 (11.9%) | 1,915 (33.2%) | 3,684 (7.5%) |
| **Marital status** |  |  |  |  |  |  |
| Never in union | 9 (0.1%) | 10 (0.1%) | 7 (0.1%) | 5 (0.1%) | 0 (0.0%) | 31 (0.1%) |
| Married/ living with partner | 14,426 (97.4%) | 11,501 (97.3%) | 8,955 (97.1%) | 7,119 (97.7%) | 5,659 (98.1%) | 47,660 (97.5%) |
| Windowed/divorced/separated | 372 (2.5%) | 308 (2.6%) | 265 (2.9%) | 160 (2.2%) | 108 (1.9%) | 1,213 (2.5%) |
| **Place of residence** |  |  |  |  |  |  |
| Urban | 2,226 (15.0%) | 3,615 (30.6%) | 4,688 (50.8%) | 5,011 (68.8%) | 4,700 (81.5%) | 20,240 (41.4%) |
| Rural | 12,581 (85.0%) | 8,204 (69.4%) | 4,539 (49.2%) | 2,273 (31.2%) | 1,067 (18.5%) | 28,664 (58.6%) |
| **Literacy** |  |  |  |  |  |  |
| Cannot real at all | 12,805 (86.5%) | 8,862 (75.0%) | 5,371 (58.3%) | 3,084 (42.4%) | 1,126 (19.5%) | 31,248 (63.9%) |
| Able to read | 1,912 (12.9%) | 2,940 (24.9%) | 3,823 (41.5%) | 4,187 (57.5%) | 4,638 (80.5%) | 17,500 (35.8%) |
| Not ascertained | 81 (0.5%) | 8 (0.1%) | 25 (0.3%) | 9 (0.1%) | 0 (0.0%) | 123 (0.3%) |
| **Birth order** | 3.300 (2.238) | 3.062 (2.132) | 2.829 (1.999) | 2.578 (1.823) | 2.212 (1.547) | 2.918 (2.067) |
| **Sex of child** |  |  |  |  |  |  |
| Male | 7,512 (50.7%) | 6,012 (50.9%) | 4,656 (50.5%) | 3,640 (50.0%) | 2,778 (48.2%) | 24,598 (50.3%) |
| Female | 7,295 (49.3%) | 5,807 (49.1%) | 4,571 (49.5%) | 3,644 (50.0%) | 2,989 (51.8%) | 24,306 (49.7%) |
| **BMI** |  |  |  |  |  |  |
| Underweight | 688 (4.6%) | 361 (3.1%) | 243 (2.6%) | 134 (1.8%) | 55 (1.0%) | 1,481 (3.0%) |
| Normal | 3,406 (23.0%) | 2,507 (21.2%) | 1,449 (15.7%) | 1,176 (16.1%) | 761 (13.2%) | 9,299 (19.0%) |
| Overweight | 1,424 (9.6%) | 1,403 (11.9%) | 1,140 (12.4%) | 1,016 (13.9%) | 828 (14.4%) | 5,811 (11.9%) |
| Obese | 9,289 (62.7%) | 7,548 (63.9%) | 6,395 (69.3%) | 4,958 (68.1%) | 4,123 (71.5%) | 32,313 (66.1%) |

**Table S3**. Baseline unweighted absolute facility birth rates (per 10,000) across wealth quintiles and countries.

| **Country** | **Poorest** | **Poorer** | **Middle** | **Richer** | **Richest** |
| --- | --- | --- | --- | --- | --- |
| Angola | 1380 | 2929 | 5863 | 7634 | 8733 |
| Bangladesh | 1574 | 2435 | 3405 | 4707 | 7173 |
| Benin | 6846 | 8207 | 8794 | 9649 | 9918 |
| Burkina Faso | 5289 | 6329 | 7466 | 8292 | 9622 |
| Burundi | 6906 | 7386 | 7704 | 7717 | 9171 |
| Cambodia | 6276 | 7005 | 7719 | 8503 | 9254 |
| Cameroon | 2439 | 5675 | 7543 | 9024 | 9633 |
| Chad | 1231 | 1419 | 1185 | 1315 | 5603 |
| Comoros | 5931 | 7302 | 8521 | 8689 | 9114 |
| Congo | 7574 | 9013 | 9549 | 9808 | 9815 |
| Congo Democratic Republic | 5780 | 6599 | 7663 | 8925 | 9856 |
| Cote d'Ivoire | 3107 | 4126 | 6182 | 8035 | 9191 |
| Dominican Republic | 9379 | 9941 | 9971 | 9964 | 9955 |
| Egypt | 8185 | 8412 | 8986 | 9410 | 9816 |
| Ethiopia | 921 | 1605 | 1647 | 2154 | 6940 |
| Gabon | 7517 | 9391 | 9543 | 9612 | 9783 |
| Gambia | 6238 | 6372 | 6979 | 8391 | 9627 |
| Ghana | 4985 | 6083 | 7915 | 9437 | 9652 |
| Guatemala | 4190 | 5960 | 7540 | 9039 | 9498 |
| Guinea | 1862 | 3359 | 4257 | 6254 | 8420 |
| Haiti | 1281 | 2365 | 4240 | 5551 | 7907 |
| Honduras | 5944 | 8099 | 9083 | 9607 | 9801 |
| India | 8007 | 8901 | 9358 | 9611 | 9788 |
| Jordan | 9788 | 9926 | 9973 | 9977 | 9668 |
| Kenya | 4016 | 6804 | 7783 | 8786 | 9464 |
| Lesotho | 6277 | 7388 | 8321 | 8983 | 9401 |
| Liberia | 5509 | 6664 | 7200 | 7712 | 8533 |
| Madagascar | 1779 | 2962 | 3901 | 5006 | 7370 |
| Malawi | 7852 | 8161 | 8343 | 8875 | 9513 |
| Maldives | 9648 | 9609 | 9314 | 9714 | 9126 |
| Mali | 3482 | 4577 | 5438 | 7710 | 9470 |
| Mauritania | 4183 | 5895 | 8421 | 9470 | 9841 |
| Mozambique | 3790 | 4574 | 5952 | 7765 | 9318 |
| Myanmar | 1781 | 2553 | 3800 | 5259 | 8038 |
| Nepal | 4019 | 4941 | 6138 | 7232 | 8850 |
| Niger | 2067 | 2650 | 2832 | 4322 | 8195 |
| Nigeria | 868 | 2162 | 4131 | 5969 | 8132 |
| Pakistan | 5364 | 6145 | 7014 | 7866 | 9120 |
| Rwanda | 8032 | 8482 | 8666 | 8869 | 9494 |
| Sierra Leone | 6489 | 6875 | 6788 | 7423 | 8112 |
| South Africa | 9080 | 9559 | 9760 | 9834 | 10000 |
| Tanzania | 4069 | 4820 | 5890 | 7306 | 9000 |
| Timor Leste | 1226 | 1933 | 2972 | 4740 | 7172 |
| Togo | 4736 | 6302 | 7780 | 9420 | 9750 |
| Uganda | 5578 | 6266 | 6791 | 7458 | 9102 |
| Zambia | 6426 | 7231 | 7712 | 8826 | 9654 |
| Zimbabwe | 5597 | 6465 | 7123 | 8636 | 9344 |

**Table S4.** Baseline unweighted absolute diphtheria-pertussis-tetanus 3rd dose (DPT3) vaccination rate (per 10,000) across wealth quintiles and countries.

| **Country** | **Poorest** | **Poorer** | **Middle** | **Richer** | **Richest** |
| --- | --- | --- | --- | --- | --- |
| Angola | 2076 | 2714 | 4311 | 6087 | 6106 |
| Bangladesh | 8854 | 9271 | 9248 | 9461 | 9644 |
| Benin | 5377 | 6340 | 6764 | 7308 | 7997 |
| Burkina Faso | 8256 | 9028 | 9184 | 9228 | 9573 |
| Burundi | 9595 | 9603 | 9639 | 9665 | 9464 |
| Cambodia | 7111 | 8105 | 8136 | 9126 | 9223 |
| Cameroon | 5014 | 6747 | 7548 | 7846 | 8734 |
| Chad | 2631 | 2872 | 2553 | 3034 | 4155 |
| Comoros | 6102 | 6700 | 8182 | 8642 | 8125 |
| Congo | 4145 | 5885 | 5254 | 6737 | 6053 |
| Congo Democratic Republic | 3826 | 4394 | 5057 | 6217 | 8165 |
| Cote d'Ivoire | 4462 | 5902 | 7109 | 7812 | 9036 |
| Dominican Republic | 8927 | 9390 | 8981 | 9351 | 9710 |
| Egypt | 9587 | 9730 | 9676 | 9746 | 9849 |
| Ethiopia | 2782 | 4769 | 5109 | 5570 | 7601 |
| Gabon | 3499 | 4952 | 5136 | 5806 | 5672 |
| Gambia | 9129 | 9032 | 9180 | 8710 | 8917 |
| Ghana | 8624 | 8852 | 8764 | 8690 | 9351 |
| Guatemala | 8078 | 8440 | 8773 | 9148 | 8986 |
| Guinea | 2848 | 4207 | 3860 | 4510 | 5860 |
| Haiti | 5177 | 5797 | 6122 | 6434 | 7239 |
| Honduras | 9615 | 9673 | 9454 | 9668 | 9829 |
| India | 8504 | 8753 | 8985 | 9037 | 9064 |
| Jordan | 8967 | 8990 | 9171 | 9176 | 8750 |
| Kenya | 8345 | 9273 | 9091 | 9062 | 9376 |
| Lesotho | 8767 | 8871 | 9344 | 8704 | 8182 |
| Liberia | 5664 | 7259 | 7692 | 7516 | 7500 |
| Madagascar | 5296 | 6316 | 7857 | 7556 | 8750 |
| Malawi | 9395 | 9353 | 9455 | 9525 | 9578 |
| Maldives | 8504 | 8472 | 8069 | 8600 | 8500 |
| Mali | 5294 | 6195 | 6236 | 6831 | 7682 |
| Mauritania | 7254 | 6995 | 8361 | 7933 | 8312 |
| Mozambique | 6577 | 7106 | 7948 | 8626 | 8998 |
| Myanmar | 5100 | 5699 | 6933 | 7688 | 8696 |
| Nepal | 8992 | 9070 | 9041 | 9361 | 9474 |
| Niger | 6604 | 6905 | 7935 | 7861 | 8955 |
| Nigeria | 1466 | 2450 | 4778 | 6459 | 8040 |
| Pakistan | 4810 | 7343 | 7377 | 7752 | 8976 |
| Rwanda | 9674 | 9792 | 9868 | 9905 | 9852 |
| Sierra Leone | 7742 | 8094 | 8244 | 8140 | 7814 |
| South Africa | 7848 | 7246 | 7015 | 7255 | 5652 |
| Tanzania | 8163 | 8779 | 8938 | 9364 | 9521 |
| Timor Leste | 5382 | 6155 | 6639 | 7299 | 7430 |
| Togo | 9194 | 8493 | 8370 | 8729 | 9216 |
| Uganda | 7925 | 7391 | 7801 | 7556 | 8093 |
| Zambia | 8341 | 8762 | 8966 | 9400 | 9527 |
| Zimbabwe | 7568 | 7852 | 8234 | 8357 | 8420 |

**Table S5.** Baseline unweighted absolute measles vaccination rates (per 10,000) across wealth quintiles and countries.

| **Country** | **Poorest** | **Poorer** | **Middle** | **Richer** | **Richest** |
| --- | --- | --- | --- | --- | --- |
| Angola | 3090 | 4126 | 6099 | 7189 | 8407 |
| Bangladesh | 8020 | 8577 | 8669 | 8830 | 9241 |
| Benin | 5456 | 6523 | 7325 | 7715 | 8591 |
| Burkina Faso | 7791 | 8854 | 9085 | 9018 | 9188 |
| Burundi | 9063 | 9428 | 9381 | 9381 | 9542 |
| Cambodia | 6717 | 7697 | 7818 | 8857 | 9128 |
| Cameroon | 5201 | 6495 | 7228 | 7742 | 8610 |
| Chad | 5200 | 5230 | 5035 | 4907 | 6604 |
| Comoros | 6870 | 6400 | 8000 | 8519 | 8438 |
| Congo | 6119 | 7115 | 7458 | 8421 | 8684 |
| Congo Democratic Republic | 5516 | 6247 | 6752 | 6997 | 8670 |
| Cote d'Ivoire | 4703 | 5912 | 6484 | 7209 | 8675 |
| Dominican Republic | 9018 | 9236 | 9151 | 9559 | 9500 |
| Egypt | 3463 | 3633 | 3849 | 4102 | 4264 |
| Ethiopia | 4166 | 5762 | 6066 | 6311 | 8127 |
| Gabon | 6053 | 7152 | 7123 | 8141 | 7368 |
| Gambia | 9279 | 9220 | 9309 | 9128 | 8535 |
| Ghana | 8519 | 8852 | 8764 | 8571 | 9351 |
| Guatemala | 6037 | 5996 | 6369 | 7018 | 7029 |
| Guinea | 3793 | 4466 | 4416 | 4921 | 7222 |
| Haiti | 6040 | 6058 | 6769 | 6875 | 7125 |
| Honduras | 9047 | 9006 | 8725 | 8907 | 9106 |
| India | 8426 | 8675 | 8894 | 8993 | 9080 |
| Jordan | 7988 | 7961 | 7790 | 8471 | 7750 |
| Kenya | 7653 | 8881 | 8885 | 9163 | 9376 |
| Lesotho | 8784 | 8710 | 9677 | 9259 | 8837 |
| Liberia | 6135 | 7440 | 7393 | 7255 | 8571 |
| Madagascar | 4721 | 5356 | 7188 | 7333 | 8478 |
| Malawi | 9337 | 9217 | 9111 | 9430 | 9577 |
| Maldives | 9206 | 8819 | 8414 | 8800 | 9000 |
| Mali | 6118 | 6727 | 6602 | 7280 | 7787 |
| Mauritania | 6649 | 7192 | 8667 | 8652 | 8438 |
| Mozambique | 7520 | 7454 | 8208 | 9054 | 9406 |
| Myanmar | 7510 | 6891 | 8267 | 8608 | 9304 |
| Nepal | 9018 | 8937 | 9074 | 9323 | 9408 |
| Niger | 6226 | 7063 | 7308 | 7861 | 8172 |
| Nigeria | 1870 | 3187 | 5065 | 6302 | 8060 |
| Pakistan | 4650 | 7019 | 7220 | 7397 | 8498 |
| Rwanda | 6454 | 7260 | 7252 | 7424 | 7135 |
| Sierra Leone | 7931 | 7545 | 7677 | 7664 | 8433 |
| South Africa | 8462 | 8841 | 9385 | 8800 | 8696 |
| Tanzania | 7828 | 8150 | 8415 | 9104 | 9227 |
| Timor Leste | 5582 | 6462 | 7355 | 7892 | 7991 |
| Togo | 8280 | 7397 | 7407 | 8220 | 8431 |
| Uganda | 7736 | 7492 | 7875 | 7788 | 8494 |
| Zambia | 8381 | 8505 | 8799 | 9154 | 9437 |
| Zimbabwe | 7905 | 7980 | 8212 | 8607 | 8649 |

**Table S6.** Baseline unweighted absolute postnatal care utilization rates (per 10,000) across wealth quintiles and countries.

| **Country** | **Poorest** | **Poorer** | **Middle** | **Richer** | **Richest** |
| --- | --- | --- | --- | --- | --- |
| Angola | 1406 | 1427 | 2421 | 2313 | 3341 |
| Bangladesh | 5074 | 5255 | 5661 | 6129 | 6802 |
| Benin | 3255 | 3548 | 3849 | 4338 | 4842 |
| Burkina Faso | 7492 | 8158 | 8539 | 8650 | 8523 |
| Burundi | 1188 | 956 | 1002 | 1157 | 1765 |
| Cambodia | 4777 | 5368 | 5812 | 6248 | 6772 |
| Cameroon | 1463 | 2388 | 2796 | 2931 | 3479 |
| Chad | 1282 | 1338 | 1074 | 1128 | 2947 |
| Comoros | 2691 | 2869 | 3303 | 3894 | 3801 |
| Congo | 4900 | 5545 | 5274 | 6088 | 6642 |
| Congo Democratic Republic | 1208 | 1456 | 1496 | 1761 | 2638 |
| Cote d'Ivoire | 6780 | 7299 | 7331 | 7466 | 7278 |
| Dominican Republic | 9077 | 9470 | 9669 | 9750 | 9921 |
| Egypt | 3216 | 3343 | 3642 | 3459 | 3904 |
| Ethiopia | 405 | 592 | 644 | 869 | 1389 |
| Gabon | 5034 | 6232 | 6318 | 6265 | 7253 |
| Gambia | 6988 | 6699 | 6321 | 5923 | 4599 |
| Ghana | 7666 | 7112 | 6936 | 7211 | 7902 |
| Guatemala | 8779 | 8855 | 8938 | 9061 | 9542 |
| Guinea | 3376 | 4224 | 4364 | 5098 | 5265 |
| Haiti | 5197 | 5929 | 6906 | 7547 | 8259 |
| Honduras | 9005 | 9529 | 9746 | 9825 | 9840 |
| India | 4554 | 4465 | 4577 | 4625 | 4925 |
| Jordan | 4518 | 4689 | 5606 | 5278 | 6135 |
| Kenya | 4105 | 4966 | 4894 | 4951 | 5459 |
| Lesotho | 6655 | 7873 | 8314 | 8553 | 8981 |
| Liberia | 4805 | 4855 | 4751 | 4631 | 4523 |
| Madagascar | 2491 | 2625 | 3436 | 3517 | 4725 |
| Malawi | 3804 | 3929 | 4069 | 4610 | 5044 |
| Maldives | 7204 | 7329 | 7233 | 7778 | 9109 |
| Mali | 2519 | 2616 | 2557 | 3268 | 4739 |
| Mauritania | 2034 | 2335 | 2432 | 1824 | 1544 |
| Myanmar | 4283 | 4824 | 5036 | 5000 | 5019 |
| Nepal | 2860 | 3476 | 3667 | 4195 | 5108 |
| Niger | 5948 | 6694 | 6493 | 6418 | 7445 |
| Nigeria | 1054 | 1691 | 2766 | 3738 | 4908 |
| Pakistan | 3065 | 3019 | 3379 | 4133 | 5351 |
| Rwanda | 2977 | 3130 | 3420 | 3321 | 3317 |
| Sierra Leone | 5534 | 6097 | 6005 | 6314 | 5498 |
| South Africa | 8092 | 8249 | 8503 | 8667 | 8138 |
| Tanzania | 2504 | 2861 | 2943 | 3259 | 3608 |
| Timor Leste | 3038 | 2909 | 3304 | 3047 | 2979 |
| Togo | 7328 | 7094 | 7878 | 7463 | 7450 |
| Uganda | 2902 | 2576 | 1827 | 2198 | 3174 |
| Zambia | 5751 | 5809 | 6065 | 6275 | 6406 |
| Zimbabwe | 6889 | 6946 | 7414 | 8073 | 8429 |

**Table S7.** Baseline unweighted absolute stunting rates (per 10,000) across wealth quintiles and countries.

| **Country** | **Poorest** | **Poorer** | **Middle** | **Richer** | **Richest** |
| --- | --- | --- | --- | --- | --- |
| Bangladesh | 4673 | 3904 | 3586 | 3032 | 2125 |
| Benin | 4213 | 3991 | 3647 | 3445 | 2646 |
| Burkina Faso | 3852 | 3270 | 3429 | 3095 | 1942 |
| Burundi | 6574 | 5765 | 5469 | 4655 | 3046 |
| Cambodia | 3948 | 3584 | 3340 | 2828 | 2077 |
| Cameroon | 3974 | 3689 | 2659 | 2121 | 1161 |
| Chad | 3952 | 4059 | 4186 | 4591 | 3197 |
| Comoros | 3421 | 2632 | 2239 | 2444 | 2030 |
| Congo | 3179 | 2377 | 2027 | 1307 | 947 |
| Congo Democratic Republic | 4110 | 4236 | 3929 | 3593 | 2000 |
| Cote d'Ivoire | 3345 | 3211 | 2643 | 2277 | 1614 |
| Dominican Republic | 1238 | 785 | 429 | 532 | 350 |
| Egypt | 2173 | 2109 | 1790 | 1923 | 1811 |
| Ethiopia | 4229 | 4396 | 3912 | 3766 | 2429 |
| Gabon | 3016 | 2029 | 1563 | 1051 | 573 |
| Gambia | 2609 | 2296 | 2159 | 1709 | 1392 |
| Ghana | 2146 | 2084 | 1384 | 1411 | 769 |
| Guatemala | 6060 | 5335 | 4100 | 2603 | 1680 |
| Guinea | 3519 | 3256 | 3440 | 2605 | 1731 |
| Haiti | 2715 | 2176 | 1616 | 1491 | 825 |
| Honduras | 3858 | 2445 | 1499 | 1033 | 664 |
| India | 4209 | 3700 | 3261 | 2745 | 2232 |
| Kenya | 2977 | 2562 | 2021 | 1760 | 1109 |
| Lesotho | 4208 | 3289 | 3484 | 2514 | 1627 |
| Liberia | 3023 | 3061 | 2923 | 2580 | 1570 |
| Madagascar | 4013 | 3871 | 3690 | 3763 | 2915 |
| Malawi | 4458 | 4181 | 3946 | 3658 | 2740 |
| Maldives | 1805 | 1569 | 1465 | 1233 | 1273 |
| Mali | 4063 | 3511 | 3542 | 2693 | 1814 |
| Mauritania | 3262 | 3069 | 2556 | 2142 | 1201 |
| Mozambique | 4863 | 4562 | 4163 | 3557 | 2281 |
| Myanmar | 3569 | 2984 | 3025 | 2074 | 1630 |
| Nepal | 4512 | 3597 | 2830 | 2584 | 1888 |
| Niger | 4199 | 4242 | 3955 | 4208 | 3200 |
| Nigeria | 5145 | 4316 | 3392 | 2381 | 1678 |
| Pakistan | 5091 | 4198 | 3483 | 2921 | 2328 |
| Rwanda | 4690 | 4402 | 3779 | 3163 | 1768 |
| Sierra Leone | 3650 | 3461 | 3547 | 2986 | 2311 |
| South Africa | 3448 | 2442 | 2658 | 1859 | 1333 |
| Tanzania | 4080 | 3846 | 3789 | 3199 | 2213 |
| Timor Leste | 5366 | 5277 | 4977 | 4629 | 4193 |
| Togo | 3014 | 3105 | 2905 | 1895 | 1226 |
| Uganda | 3364 | 2918 | 3293 | 2600 | 1669 |
| Zambia | 4158 | 3931 | 3596 | 3528 | 2499 |
| Zimbabwe | 3323 | 2920 | 3049 | 2776 | 1913 |

**Table S8.** Baseline unweighted absolute wasting rates (per 10,000) across wealth quintiles and countries.

| **Country** | **Poorest** | **Poorer** | **Middle** | **Richer** | **Richest** |
| --- | --- | --- | --- | --- | --- |
| Bangladesh | 1709 | 1474 | 1322 | 1180 | 1015 |
| Benin | 1404 | 1289 | 1303 | 1115 | 1035 |
| Burkina Faso | 2210 | 1921 | 1843 | 1826 | 1338 |
| Burundi | 866 | 749 | 639 | 625 | 431 |
| Cambodia | 1744 | 1436 | 1435 | 1335 | 1020 |
| Cameroon | 1127 | 706 | 458 | 321 | 277 |
| Chad | 1632 | 1579 | 1757 | 1709 | 1704 |
| Comoros | 1577 | 1439 | 1245 | 1036 | 914 |
| Congo | 685 | 609 | 507 | 578 | 700 |
| Congo Democratic Republic | 1116 | 1083 | 1101 | 891 | 491 |
| Cote d'Ivoire | 1007 | 847 | 891 | 681 | 712 |
| Dominican Republic | 305 | 231 | 199 | 206 | 225 |
| Egypt | 857 | 911 | 1117 | 1407 | 1215 |
| Ethiopia | 1815 | 1364 | 1231 | 1035 | 754 |
| Gabon | 519 | 355 | 336 | 389 | 385 |
| Gambia | 778 | 1010 | 1073 | 781 | 632 |
| Ghana | 874 | 520 | 261 | 629 | 347 |
| Guatemala | 96 | 65 | 81 | 88 | 87 |
| Guinea | 1240 | 1135 | 1018 | 1053 | 838 |
| Haiti | 546 | 473 | 499 | 517 | 329 |
| Honduras | 214 | 137 | 150 | 103 | 135 |
| India | 2401 | 2080 | 1922 | 1794 | 1586 |
| Kenya | 1580 | 514 | 720 | 624 | 464 |
| Lesotho | 586 | 543 | 317 | 337 | 120 |
| Liberia | 629 | 559 | 684 | 723 | 822 |
| Madagascar | 1028 | 745 | 718 | 550 | 742 |
| Malawi | 460 | 419 | 467 | 347 | 341 |
| Maldives | 937 | 972 | 843 | 833 | 1296 |
| Mali | 1742 | 1596 | 1300 | 1130 | 1046 |
| Mauritania | 978 | 809 | 851 | 571 | 291 |
| Mozambique | 1231 | 792 | 603 | 441 | 257 |
| Myanmar | 863 | 609 | 714 | 586 | 887 |
| Nepal | 1594 | 1438 | 1438 | 1510 | 962 |
| Niger | 2708 | 2341 | 1979 | 2229 | 1604 |
| Nigeria | 2134 | 1813 | 1498 | 1324 | 1163 |
| Pakistan | 1471 | 1137 | 975 | 945 | 713 |
| Rwanda | 283 | 257 | 260 | 179 | 217 |
| Sierra Leone | 769 | 770 | 759 | 940 | 817 |
| South Africa | 276 | 288 | 374 | 268 | 137 |
| Tanzania | 608 | 585 | 679 | 639 | 559 |
| Timor Leste | 2538 | 2488 | 2339 | 2190 | 1817 |
| Togo | 1088 | 862 | 835 | 745 | 588 |
| Uganda | 831 | 638 | 499 | 300 | 340 |
| Zambia | 638 | 579 | 458 | 634 | 555 |
| Zimbabwe | 491 | 471 | 335 | 419 | 170 |

**Table S9.** Facility birth rate change (per 10,000) (with 95% confidence interval and p value) associated with achieving recommended antenatal care visits and quality.

| **Country** | **Poorest** | **Poorer** | **Middle** | **Richer** | **Richest** |
| --- | --- | --- | --- | --- | --- |
| Angola | 1332 (1200, 1464) (p<0.001) | 1200 (1020, 1380) (p<0.001) | 764 (661, 866) (p<0.001) | 294 (186, 401) (p<0.001) | 116 (56, 176)  (p<0.001) |
| Bangladesh | 2022 (1861, 2183) (p<0.001) | 2168 (2022, 2313) (p<0.001) | 2336 (2104, 2567) (p<0.001) | 1759 (1600, 1919) (p<0.001) | 1098 (996, 1200) (p<0.001) |
| Benin | 2278 (2108, 2447) (p<0.001) | 1408 (1274, 1542) (p<0.001) | 806 (696, 916) (p<0.001) | 202 (159, 244) (p<0.001) | 15 (-7, 37)  (p=0.179) |
| Burkina Faso | 2647 (1941, 3352) (p<0.001) | 2006 (1432, 2580) (p<0.001) | 1085 (378, 1791) (p=0.003) | 765 (335, 1196) (p<0.001) | 196 (87, 305)  (p<0.001) |
| Burundi | 1344 (1060, 1628) (p<0.001) | 620 (172, 1068) (p=0.007) | 1098 (801, 1395) (p<0.001) | 1361 (1042, 1680) (p<0.001) | 411 (221, 600) (p<0.001) |
| Cambodia | 2284 (2097, 2472) (p<0.001) | 1749 (1556, 1942) (p<0.001) | 1312 (1104, 1520) (p<0.001) | 812 (700, 925) (p<0.001) | 289 (210, 369) (p<0.001) |
| Cameroon | 1834 (1646, 2022) (p<0.001) | 1501 (1345, 1658) (p<0.001) | 869 (764, 974) (p<0.001) | 295 (244, 346) (p<0.001) | 61 (34, 87)  (p<0.001) |
| Chad | 1569 (1192, 1946) (p<0.001) | 1878 (1498, 2258) (p<0.001) | 1539 (1202, 1876) (p<0.001) | 1578 (1222, 1934) (p<0.001) | 1443 (1234, 1651) (p<0.001) |
| Comoros | 989 (627, 1351) (p<0.001) | 530 (157, 903) (p=0.005) | 420 (180, 661) (p<0.001) | -79 (-333, 175) (p=0.553) | 106 (-78, 289) (p=0.261) |
| Congo | 930 (723, 1136) (p<0.001) | 353 (212, 493) (p<0.001) | 108 (42, 173)  (p=0.001) | 21 (8, 35)  (p=0.002) | 1 (-4, 5)  (p=0.82) |
| Congo Democratic Republic | 1890 (1489, 2292) (p<0.001) | 1112 (691, 1532) (p<0.001) | 891 (615, 1168) (p<0.001) | 397 (212, 583) (p<0.001) | 79 (29, 129)  (p=0.002) |
| Cote d'Ivoire | 1983 (1526, 2441) (p<0.001) | 1430 (1075, 1786) (p<0.001) | 866 (502, 1230) (p<0.001) | 444 (213, 675) (p<0.001) | 282 (71, 494)  (p=0.009) |
| Dominican Republic | 117 (63, 171)  (p<0.001) | 8 (0, 17)  (p=0.045) | 10 (-16, 36)  (p=0.458) | 10 (-14, 34)  (p=0.422) | 4 (-2, 10)  (p=0.193) |
| Egypt | 129 (9, 249)  (p=0.035) | 115 (3, 227)  (p=0.045) | 26 (-51, 104)  (p=0.515) | 3 (-28, 34)  (p=0.842) | 9 (1, 17)  (p=0.02) |
| Ethiopia | 2722 (2373, 3071) (p<0.001) | 3372 (2844, 3899) (p<0.001) | 2230 (1847, 2614) (p<0.001) | 1999 (1588, 2411) (p<0.001) | 1535 (1362, 1709) (p<0.001) |
| Gabon | 1002 (885, 1119) (p<0.001) | 220 (149, 291) (p<0.001) | 61 (6, 117)  (p=0.03) | 117 (31, 204)  (p=0.008) | 21 (7, 35)  (p=0.004) |
| Gambia | 181 (119, 243) (p<0.001) | 143 (79, 208)  (p<0.001) | 57 (-42, 157)  (p=0.261) | 94 (28, 161)  (p=0.005) | 38 (10, 67)  (p=0.009) |
| Ghana | 494 (378, 609) (p<0.001) | 356 (218, 494) (p<0.001) | 155 (73, 236)  (p<0.001) | 59 (17, 100)  (p=0.005) | 32 (-26, 90)  (p=0.285) |
| Guatemala | 1231 (1034, 1427) (p<0.001) | 941 (751, 1130) (p<0.001) | 647 (530, 764) (p<0.001) | 185 (118, 252) (p<0.001) | 121 (58, 184)  (p<0.001) |
| Guinea | 1683 (1350, 2017) (p<0.001) | 1035 (641, 1429) (p<0.001) | 1437 (1120, 1754) (p<0.001) | 676 (426, 925) (p<0.001) | 199 (114, 284) (p<0.001) |
| Haiti | 459 (378, 541) (p<0.001) | 575 (436, 714) (p<0.001) | 597 (493, 700) (p<0.001) | 472 (378, 566) (p<0.001) | 241 (151, 331) (p<0.001) |
| Honduras | 706 (610, 803) (p<0.001) | 387 (290, 483) (p<0.001) | 177 (125, 229) (p<0.001) | 73 (43, 103)  (p<0.001) | 33 (7, 58)  (p=0.011) |
| India | 349 (329, 369) (p<0.001) | 182 (169, 196) (p<0.001) | 126 (116, 135) (p<0.001) | 65 (57, 73)  (p<0.001) | 28 (24, 32)  (p<0.001) |
| Jordan | 29 (15, 43)  (p<0.001) | 16 (6, 25)  (p=0.001) | 3 (-1, 7)  (p=0.137) | 4 (0, 7)  (p=0.029) | -5 (-13, 3)  (p=0.191) |
| Kenya | 808 (726, 890) (p<0.001) | 619 (497, 741) (p<0.001) | 478 (393, 563) (p<0.001) | 238 (192, 284) (p<0.001) | 72 (51, 93)  (p<0.001) |
| Lesotho | 480 (215, 745) (p<0.001) | 554 (173, 934) (p=0.004) | 266 (89, 443)  (p=0.003) | 42 (-90, 173)  (p=0.544) | 96 (33, 159)  (p=0.003) |
| Liberia | 779 (652, 906) (p<0.001) | 517 (380, 653) (p<0.001) | 259 (148, 369) (p<0.001) | 247 (148, 347) (p<0.001) | 90 (-1, 181)  (p=0.052) |
| Madagascar | 1335 (726, 1945) (p<0.001) | 1717 (1208, 2225) (p<0.001) | 746 (259, 1233) (p=0.003) | 796 (353, 1238) (p<0.001) | 512 (227, 797) (p<0.001) |
| Malawi | 952 (815, 1088) (p<0.001) | 752 (619, 886) (p<0.001) | 568 (365, 770) (p<0.001) | 572 (463, 681) (p<0.001) | 87 (51, 124)  (p<0.001) |
| Maldives | 28 (11, 45)  (p=0.002) | 9 (-5, 23)  (p=0.205) | 12 (-27, 50)  (p=0.562) | 25 (-10, 60)  (p=0.156) | -7 (-26, 11)  (p=0.454) |
| Mali | 3016 (2611, 3421) (p<0.001) | 2315 (1760, 2870) (p<0.001) | 2179 (1829, 2529) (p<0.001) | 1373 (1196, 1551) (p<0.001) | 262 (216, 308) (p<0.001) |
| Mauritania | 2241 (1668, 2813) (p<0.001) | 1988 (1528, 2448) (p<0.001) | 664 (471, 857) (p<0.001) | 153 (38, 269)  (p=0.009) | 73 (4, 141)  (p=0.037) |
| Mozambique | 1339 (979, 1699) (p<0.001) | 1420 (975, 1864) (p<0.001) | 1076 (778, 1374) (p<0.001) | 741 (518, 964) (p<0.001) | 121 (15, 227)  (p=0.024) |
| Myanmar | 1257 (1104, 1411) (p<0.001) | 1042 (803, 1281) (p<0.001) | 1025 (696, 1354) (p<0.001) | 718 (430, 1007) (p<0.001) | 443 (238, 648) (p<0.001) |
| Nepal | 2192 (1947, 2436) (p<0.001) | 1862 (1631, 2093) (p<0.001) | 1534 (1333, 1736) (p<0.001) | 894 (720, 1069) (p<0.001) | 387 (231, 543) (p<0.001) |
| Niger | 1926 (891, 2961) (p<0.001) | 1317 (458, 2176) (p=0.003) | 981 (-102, 2063) (p=0.076) | 1720 (1122, 2318) (p<0.001) | 974 (736, 1212) (p<0.001) |
| Nigeria | 1111 (985, 1238) (p<0.001) | 1023 (882, 1164) (p<0.001) | 1041 (946, 1137) (p<0.001) | 678 (614, 742) (p<0.001) | 405 (344, 467) (p<0.001) |
| Pakistan | 1835 (1336, 2335) (p<0.001) | 1981 (1587, 2375) (p<0.001) | 1126 (799, 1453) (p<0.001) | 579 (350, 808) (p<0.001) | 221 (120, 323) (p<0.001) |
| Rwanda | 979 (802, 1157) (p<0.001) | 728 (536, 920) (p<0.001) | 801 (631, 971) (p<0.001) | 693 (558, 828) (p<0.001) | 270 (196, 344) (p<0.001) |
| Sierra Leone | 453 (380, 527) (p<0.001) | 385 (254, 516) (p<0.001) | 325 (254, 397) (p<0.001) | 249 (147, 352) (p<0.001) | 109 (2, 217)  (p=0.046) |
| South Africa | 65 (-5, 135)  (p=0.069) | 30 (-9, 70)  (p=0.132) | 15 (0, 29)  (p=0.053) | 7 (-4, 18)  (p=0.217) | 0 (-2, 2)  (p=0.998) |
| Tanzania | 819 (301, 1337) (p=0.002) | 932 (483, 1380) (p<0.001) | 533 (136, 931) (p=0.009) | 93 (-169, 356) (p=0.496) | 112 (-53, 278) (p=0.185) |
| Timor Leste | 781 (560, 1003) (p<0.001) | 1329 (974, 1684) (p<0.001) | 1778 (1386, 2169) (p<0.001) | 1741 (1417, 2064) (p<0.001) | 885 (696, 1074) (p<0.001) |
| Togo | 1735 (882, 2588) (p<0.001) | 1358 (501, 2215) (p=0.002) | 1066 (518, 1615) (p<0.001) | 211 (105, 317) (p<0.001) | 167 (47, 288)  (p=0.007) |
| Uganda | 1272 (881, 1663) (p<0.001) | 1621 (1179, 2063) (p<0.001) | 1371 (918, 1823) (p<0.001) | 1191 (795, 1587) (p<0.001) | 362 (234, 490) (p<0.001) |
| Zambia | 1324 (1186, 1463) (p<0.001) | 657 (515, 798) (p<0.001) | 633 (506, 760) (p<0.001) | 255 (169, 342) (p<0.001) | 120 (73, 168)  (p<0.001) |
| Zimbabwe | 1599 (1423, 1774) (p<0.001) | 1279 (1120, 1439) (p<0.001) | 1104 (940, 1268) (p<0.001) | 507 (386, 627) (p<0.001) | 239 (157, 320) (p<0.001) |

**Table S10.** Diphtheria-pertussis-tetanus 3rd dose (DPT3) vaccination rate change (per 10,000) (with 95% confidence interval and p value) associated with achieving recommended antenatal care visits and quality.

| **Country** | **Poorest** | **Poorer** | **Middle** | **Richer** | **Richest** |
| --- | --- | --- | --- | --- | --- |
| Angola | 1336 (1126, 1546) (p<0.001) | 1061 (855, 1267) (p<0.001) | 542 (404, 680) (p<0.001) | 330 (136, 524) (p<0.001) | 99 (6, 191)  (p=0.036) |
| Bangladesh | 510 (427, 593) (p<0.001) | 362 (303, 420) (p<0.001) | 294 (246, 342) (p<0.001) | 201 (160, 243) (p<0.001) | 78 (58, 97)  (p<0.001) |
| Benin | 1767 (1533, 2000) (p<0.001) | 954 (771, 1138) (p<0.001) | 561 (435, 687) (p<0.001) | 224 (147, 300) (p<0.001) | 177 (117, 237) (p<0.001) |
| Burkina Faso | 1049 (871, 1228) (p<0.001) | -100 (-900, 700) (p=0.818) | 629 (512, 746) (p<0.001) | 398 (294, 503) (p<0.001) | 280 (217, 343) (p<0.001) |
| Burundi | 217 (170, 264) (p<0.001) | 142 (106, 178) (p<0.001) | 109 (78, 141)  (p<0.001) | 65 (37, 94)  (p<0.001) | -11 (-382, 360) (p=0.959) |
| Cambodia | 738 (583, 893) (p<0.001) | 442 (281, 602) (p<0.001) | 424 (311, 537) (p<0.001) | 132 (72, 192)  (p<0.001) | 116 (60, 172)  (p<0.001) |
| Cameroon | 1565 (1401, 1729) (p<0.001) | 718 (590, 846) (p<0.001) | 414 (332, 496) (p<0.001) | 156 (113, 199) (p<0.001) | 50 (30, 70)  (p<0.001) |
| Chad | 1993 (1467, 2518) (p<0.001) | 1469 (697, 2242) (p<0.001) | 1296 (880, 1711) (p<0.001) | 1525 (1074, 1976) (p<0.001) | 325 (41, 608)  (p=0.025) |
| Comoros | 715 (296, 1135) (p<0.001) | 334 (-26, 694) (p=0.068) | 147 (-19, 313) (p=0.082) | 305 (52, 558)  (p=0.018) | 150 (-35, 334) (p=0.111) |
| Congo | 655 (323, 988) (p<0.001) | 526 (129, 923) (p=0.009) | 148 (-35, 330) (p=0.114) | 49 (1, 97)  (p=0.044) | 11 (-15, 38)  (p=0.41) |
| Congo Democratic Republic | 1409 (1185, 1633) (p<0.001) | 681 (88, 1274) (p=0.024) | 1181 (725, 1637) (p<0.001) | 610 (223, 997) (p=0.002) | 278 (49, 506)  (p=0.017) |
| Cote d'Ivoire | 1330 (1080, 1581) (p<0.001) | 2011 (1325, 2697) (p<0.001) | 519 (327, 710) (p<0.001) | 413 (8, 817)  (p=0.045) | 123 (28, 219)  (p=0.011) |
| Dominican Republic | 88 (31, 146)  (p=0.003) | 42 (2, 83)  (p=0.041) | 6 (-2, 14)  (p=0.162) | 0 (0, 0)  (NA) | 0 (0, 0)  (NA) |
| Egypt | 42 (25, 59)  (p<0.001) | 36 (23, 48)  (p<0.001) | 25 (16, 35)  (p<0.001) | 19 (10, 28)  (p<0.001) | 6 (3, 9)  (p<0.001) |
| Ethiopia | 2664 (2447, 2882) (p<0.001) | 2470 (1765, 3174) (p<0.001) | 1973 (1740, 2205) (p<0.001) | 1144 (691, 1598) (p<0.001) | 1047 (733, 1361) (p<0.001) |
| Gabon | 306 (170, 441) (p<0.001) | 111 (-1, 224)  (p=0.052) | 95 (5, 185)  (p=0.039) | 70 (-186, 325) (p=0.606) | 22 (-4, 48)  (p=0.1) |
| Gambia | 67 (48, 86)  (p<0.001) | 40 (25, 54)  (p<0.001) | 54 (35, 73)  (p<0.001) | -10 (-39, 19)  (p=0.494) | -13 (-28, 1)  (p=0.075) |
| Ghana | 233 (118, 348) (p<0.001) | 196 (50, 342)  (p=0.008) | 95 (4, 185)  (p=0.04) | -5 (-20, 11)  (p=0.552) | 4 (-1, 9)  (p=0.125) |
| Guatemala | 192 (-44, 428) (p=0.111) | 148 (-21, 316) (p=0.085) | 202 (82, 321)  (p=0.001) | 37 (-10, 85)  (p=0.123) | 17 (-6, 40)  (p=0.158) |
| Guinea | 961 (420, 1502) (p<0.001) | 1255 (527, 1984) (p<0.001) | 242 (-213, 698) (p=0.301) | 332 (-43, 707) (p=0.083) | 142 (56, 229)  (p=0.001) |
| Haiti | 916 (739, 1092) (p<0.001) | 740 (485, 995) (p<0.001) | 415 (295, 534) (p<0.001) | 377 (244, 511) (p<0.001) | 174 (62, 286)  (p=0.002) |
| Honduras | 61 (40, 82)  (p<0.001) | 42 (28, 57)  (p<0.001) | 37 (14, 60)  (p=0.002) | 18 (6, 30)  (p=0.003) | 3 (0, 6)  (p=0.023) |
| India | 188 (166, 210) (p<0.001) | 132 (117, 147) (p<0.001) | 84 (74, 93)  (p<0.001) | 66 (56, 76)  (p<0.001) | 32 (21, 42)  (p<0.001) |
| Jordan | 46 (21, 72)  (p<0.001) | 25 (4, 46)  (p=0.019) | 10 (2, 18)  (p=0.011) | 9 (-3, 21)  (p=0.153) | 3 (-15, 20)  (p=0.755) |
| Kenya | 317 (263, 371) (p<0.001) | 106 (68, 145)  (p<0.001) | 97 (60, 133)  (p<0.001) | 17 (-19, 53)  (p=0.352) | 13 (0, 27)  (p=0.058) |
| Lesotho | 151 (81, 221)  (p<0.001) | 105 (54, 156)  (p<0.001) | 102 (38, 166)  (p=0.002) | 34 (12, 56)  (p=0.003) | -64 (-518, 391) (p=0.796) |
| Liberia | 361 (279, 444) (p<0.001) | 467 (275, 659) (p<0.001) | 133 (-5, 271)  (p=0.059) | 91 (2, 180)  (p=0.044) | 81 (-11, 174)  (p=0.085) |
| Madagascar | 1407 (953, 1862) (p<0.001) | 566 (-552, 1684) (p=0.326) | -260 (-895, 376) (p=0.431) | -26 (-257, 204) (p=0.835) | 68 (-15, 151)  (p=0.106) |
| Malawi | 224 (183, 265) (p<0.001) | 163 (121, 206) (p<0.001) | 126 (96, 157)  (p<0.001) | 72 (45, 98)  (p<0.001) | 82 (65, 99)  (p<0.001) |
| Maldives | 62 (-10, 134)  (p=0.093) | 48 (11, 84)  (p=0.011) | 45 (0, 90)  (p=0.049) | 30 (-9, 70)  (p=0.135) | 0 (0, 0)  (NA) |
| Mali | 2270 (1981, 2560) (p<0.001) | 1649 (1369, 1928) (p<0.001) | 871 (386, 1357) (p<0.001) | 611 (160, 1061) (p=0.008) | 427 (303, 550) (p<0.001) |
| Mauritania | 937 (441, 1433) (p<0.001) | 1295 (687, 1903) (p<0.001) | 449 (205, 693) (p<0.001) | 244 (141, 347) (p<0.001) | 21 (-157, 200) (p=0.825) |
| Mozambique | 1653 (1332, 1974) (p<0.001) | 1311 (1044, 1579) (p<0.001) | 1236 (983, 1489) (p<0.001) | 523 (186, 860) (p=0.002) | 372 (293, 451) (p<0.001) |
| Myanmar | 1056 (745, 1366) (p<0.001) | 759 (515, 1004) (p<0.001) | 610 (-131, 1351) (p=0.107) | 333 (104, 562) (p=0.004) | 30 (-16, 77)  (p=0.203) |
| Nepal | 335 (249, 422) (p<0.001) | 221 (161, 281) (p<0.001) | 127 (-25, 278) (p=0.101) | 83 (55, 112)  (p<0.001) | 21 (9, 33)  (p<0.001) |
| Niger | 1171 (987, 1356) (p<0.001) | 576 (-412, 1565) (p=0.256) | 709 (36, 1383) (p=0.039) | 439 (285, 593) (p<0.001) | 350 (265, 434) (p<0.001) |
| Nigeria | 1570 (1403, 1737) (p<0.001) | 1166 (883, 1450) (p<0.001) | 869 (751, 987) (p<0.001) | 446 (368, 525) (p<0.001) | 227 (133, 320) (p<0.001) |
| Pakistan | 948 (763, 1132) (p<0.001) | 590 (444, 736) (p<0.001) | 586 (132, 1041) (p=0.011) | 408 (161, 656) (p=0.001) | 58 (23, 94)  (p=0.001) |
| Rwanda | 87 (63, 111)  (p<0.001) | 52 (37, 68)  (p<0.001) | 38 (26, 49)  (p<0.001) | 25 (15, 34)  (p<0.001) | 27 (19, 35)  (p<0.001) |
| Sierra Leone | 254 (155, 354) (p<0.001) | 121 (32, 209)  (p=0.007) | 90 (10, 170)  (p=0.027) | -22 (-109, 65) (p=0.632) | 41 (-50, 132)  (p=0.384) |
| South Africa | 74 (-4, 152)  (p=0.062) | 24 (-32, 81)  (p=0.407) | 78 (-63, 219)  (p=0.283) | 20 (-110, 149) (p=0.779) | 105 (6, 204)  (p=0.038) |
| Tanzania | 358 (61, 654)  (p=0.018) | 130 (-281, 542) (p=0.545) | 79 (-139, 298) (p=0.486) | -10 (-120, 99) (p=0.862) | 112 (31, 193)  (p=0.007) |
| Timor Leste | 1451 (1001, 1902) (p<0.001) | 917 (530, 1303) (p<0.001) | 903 (539, 1268) (p<0.001) | 382 (68, 695)  (p=0.017) | 400 (97, 702)  (p=0.01) |
| Togo | -312 (-1009, 384) (p=0.386) | -604 (-2150, 942) (p=0.453) | -476 (-1156, 204) (p=0.171) | -459 (-832, -85) (p=0.016) | 0 (-34, 34)  (p=0.996) |
| Uganda | 637 (455, 820) (p<0.001) | 119 (-497, 735) (p=0.717) | -67 (-531, 398) (p=0.791) | 262 (85, 439)  (p=0.004) | 295 (138, 452) (p<0.001) |
| Zambia | 506 (442, 570) (p<0.001) | 277 (228, 326) (p<0.001) | 193 (153, 233) (p<0.001) | 147 (50, 243)  (p=0.003) | 74 (60, 88)  (p<0.001) |
| Zimbabwe | 1073 (815, 1331) (p<0.001) | 760 (538, 982) (p<0.001) | 649 (433, 864) (p<0.001) | 397 (250, 545) (p<0.001) | 235 (72, 398)  (p=0.005) |

**Table S11.** Measles vaccination rate change (per 10,000) (with 95% confidence interval and p value) associated with achieving recommended antenatal care visits and quality.

| **Country** | **Poorest** | **Poorer** | **Middle** | **Richer** | **Richest** |
| --- | --- | --- | --- | --- | --- |
| Angola | 1691 (1455, 1927) (p<0.001) | 1117 (908, 1326) (p<0.001) | 637 (489, 785) (p<0.001) | 394 (178, 609) (p<0.001) | 85 (0, 171)  (p=0.05) |
| Bangladesh | 803 (690, 915) (p<0.001) | 609 (528, 689) (p<0.001) | 452 (378, 526) (p<0.001) | 338 (283, 393) (p<0.001) | 136 (105, 167) (p<0.001) |
| Benin | 1746 (1500, 1992) (p<0.001) | 1000 (771, 1230) (p<0.001) | 667 (531, 802) (p<0.001) | 316 (230, 401) (p<0.001) | 194 (141, 247) (p<0.001) |
| Burkina Faso | 1299 (693, 1905) (p<0.001) | 113 (-657, 883) (p=0.786) | 509 (212, 806) (p<0.001) | 46 (-494, 587) (p=0.876) | 341 (175, 506) (p<0.001) |
| Burundi | 493 (365, 621) (p<0.001) | 299 (203, 394) (p<0.001) | 290 (188, 393) (p<0.001) | -170 (-698, 357) (p=0.538) | 180 (126, 233) (p<0.001) |
| Cambodia | 656 (514, 799) (p<0.001) | 532 (175, 888) (p=0.004) | 411 (113, 710) (p=0.007) | 146 (88, 204)  (p<0.001) | 103 (-57, 263) (p=0.207) |
| Cameroon | 1469 (1311, 1627) (p<0.001) | 645 (528, 763) (p<0.001) | 309 (235, 384) (p<0.001) | 149 (52, 247)  (p=0.003) | 42 (25, 59)  (p<0.001) |
| Chad | 2148 (1622, 2674) (p<0.001) | 1399 (718, 2080) (p<0.001) | 2175 (1524, 2827) (p<0.001) | 1806 (1256, 2356) (p<0.001) | 778 (438, 1119) (p<0.001) |
| Comoros | 561 (102, 1020) (p=0.017) | 196 (-303, 695) (p=0.45) | 137 (-84, 358) (p=0.226) | 81 (-76, 238)  (p=0.315) | 68 (-50, 186)  (p=0.264) |
| Congo | 659 (300, 1019) (p<0.001) | 448 (179, 716) (p=0.001) | 200 (71, 328)  (p=0.002) | 93 (-170, 355) (p=0.498) | 12 (-2, 25)  (p=0.098) |
| Congo Democratic Republic | 1512 (1082, 1942) (p<0.001) | 771 (315, 1226) (p<0.001) | 947 (542, 1352) (p<0.001) | 568 (326, 810) (p<0.001) | 181 (86, 277)  (p<0.001) |
| Cote d'Ivoire | 1323 (1055, 1591) (p<0.001) | 1323 (866, 1781) (p<0.001) | 587 (398, 776) (p<0.001) | 533 (230, 835) (p<0.001) | 288 (86, 490)  (p=0.005) |
| Dominican Republic | 101 (15, 187)  (p=0.021) | 26 (-1, 53)  (p=0.059) | 4 (-1, 8)  (p=0.107) | 0 (0, 0)  (NA) | 0 (0, 0)  (NA) |
| Egypt | -70 (-227, 87) (p=0.389) | -80 (-238, 78) (p=0.326) | -59 (-170, 52) (p=0.303) | -50 (-162, 61) (p=0.384) | -17 (-68, 33)  (p=0.508) |
| Ethiopia | 2793 (2474, 3112) (p<0.001) | 1974 (1653, 2295) (p<0.001) | 1507 (913, 2101) (p<0.001) | 1161 (665, 1657) (p<0.001) | 758 (606, 909) (p<0.001) |
| Gabon | 340 (160, 520) (p<0.001) | 130 (49, 211)  (p=0.002) | 76 (24, 128)  (p=0.004) | 10 (-43, 63)  (p=0.719) | -30 (-96, 36)  (p=0.379) |
| Gambia | 57 (36, 79)  (p<0.001) | 43 (27, 59)  (p<0.001) | 41 (22, 60)  (p<0.001) | -4 (-26, 19)  (p=0.754) | 37 (-37, 110)  (p=0.332) |
| Ghana | 229 (113, 345) (p<0.001) | 195 (49, 341)  (p=0.009) | 100 (3, 197)  (p=0.043) | 11 (-7, 29)  (p=0.229) | 2 (-4, 8)  (p=0.469) |
| Guatemala | 81 (-210, 373) (p=0.597) | 63 (-143, 269) (p=0.561) | 17 (-167, 202) (p=0.863) | 34 (-37, 106)  (p=0.351) | 2 (-38, 42)  (p=0.919) |
| Guinea | 430 (-15, 874) (p=0.058) | 318 (-200, 835) (p=0.231) | -261 (-663, 141) (p=0.204) | 81 (-115, 277) (p=0.425) | 212 (93, 330)  (p<0.001) |
| Haiti | 624 (510, 737) (p<0.001) | 308 (177, 439) (p<0.001) | 273 (197, 349) (p<0.001) | 240 (148, 333) (p<0.001) | 83 (23, 144)  (p=0.007) |
| Honduras | 119 (76, 163)  (p<0.001) | 87 (55, 119)  (p<0.001) | 32 (11, 54)  (p=0.003) | 17 (-3, 36)  (p=0.09) | 6 (1, 11)  (p=0.031) |
| India | 176 (157, 194) (p<0.001) | 130 (116, 144) (p<0.001) | 68 (57, 78)  (p<0.001) | 60 (52, 68)  (p<0.001) | 36 (29, 44)  (p<0.001) |
| Jordan | 62 (19, 105)  (p=0.005) | 24 (-9, 56)  (p=0.157) | 11 (-5, 27)  (p=0.189) | 10 (-8, 28)  (p=0.266) | -3 (-21, 15)  (p=0.74) |
| Kenya | 399 (331, 468) (p<0.001) | 189 (80, 297)  (p<0.001) | 124 (70, 178)  (p<0.001) | 87 (59, 114)  (p<0.001) | 33 (23, 43)  (p<0.001) |
| Lesotho | 125 (49, 201)  (p=0.001) | 98 (38, 158)  (p=0.001) | 61 (9, 114)  (p=0.021) | 3 (-59, 65)  (p=0.935) | 46 (-9, 101)  (p=0.102) |
| Liberia | 365 (280, 450) (p<0.001) | 230 (154, 305) (p<0.001) | 181 (52, 310)  (p=0.006) | 79 (-6, 165)  (p=0.07) | 93 (16, 171)  (p=0.018) |
| Madagascar | 1661 (1157, 2165) (p<0.001) | 415 (-572, 1401) (p=0.418) | -387 (-960, 185) (p=0.186) | 236 (-184, 656) (p=0.274) | 161 (47, 274)  (p=0.006) |
| Malawi | 97 (-114, 309) (p=0.372) | -108 (-397, 181) (p=0.472) | -286 (-633, 61) (p=0.106) | -106 (-329, 117) (p=0.358) | 30 (-47, 107)  (p=0.458) |
| Maldives | 37 (-9, 84)  (p=0.118) | 27 (5, 48)  (p=0.014) | 24 (-8, 56)  (p=0.137) | 16 (-8, 39)  (p=0.19) | 0 (0, 0)  (NA) |
| Mali | 1773 (1349, 2198) (p<0.001) | 1244 (767, 1722) (p<0.001) | 809 (349, 1270) (p<0.001) | 412 (25, 798)  (p=0.037) | 296 (129, 463) (p<0.001) |
| Mauritania | 1467 (1200, 1735) (p<0.001) | 1325 (779, 1872) (p<0.001) | 353 (93, 613)  (p=0.008) | 256 (154, 358) (p<0.001) | 175 (-48, 397) (p=0.124) |
| Mozambique | 1312 (1050, 1575) (p<0.001) | 1096 (812, 1381) (p<0.001) | 533 (223, 844) (p<0.001) | 488 (249, 727) (p<0.001) | 220 (127, 313) (p<0.001) |
| Myanmar | 716 (472, 960) (p<0.001) | 213 (-434, 859) (p=0.529) | 361 (229, 494) (p<0.001) | 270 (153, 387) (p<0.001) | 87 (35, 140)  (p=0.001) |
| Nepal | 321 (236, 406) (p<0.001) | 224 (164, 284) (p<0.001) | 115 (13, 216)  (p=0.028) | 83 (53, 112)  (p<0.001) | 21 (9, 32)  (p<0.001) |
| Niger | 1180 (946, 1414) (p<0.001) | -40 (-1465, 1385) (p=0.96) | -135 (-1393, 1123) (p=0.844) | 614 (414, 814) (p<0.001) | 367 (261, 473) (p<0.001) |
| Nigeria | 1548 (1325, 1770) (p<0.001) | 1090 (750, 1429) (p<0.001) | 776 (646, 907) (p<0.001) | 330 (198, 463) (p<0.001) | 190 (131, 249) (p<0.001) |
| Pakistan | 890 (708, 1071) (p<0.001) | 598 (471, 724) (p<0.001) | 427 (-24, 878) (p=0.063) | 597 (409, 785) (p<0.001) | 81 (41, 120)  (p<0.001) |
| Rwanda | 294 (45, 543)  (p=0.021) | 203 (-47, 453) (p=0.111) | 103 (-98, 304) (p=0.321) | 17 (-178, 212) (p=0.878) | 41 (-220, 302) (p=0.772) |
| Sierra Leone | 281 (190, 372) (p<0.001) | 43 (-73, 160)  (p=0.475) | -12 (-122, 97) (p=0.837) | 79 (-155, 313) (p=0.517) | 79 (12, 147)  (p=0.022) |
| South Africa | 70 (21, 118)  (p=0.005) | 61 (-2, 125)  (p=0.059) | 81 (8, 154)  (p=0.03) | 26 (-2, 54)  (p=0.067) | 58 (-71, 186)  (p=0.384) |
| Tanzania | 368 (-43, 779) (p=0.079) | 85 (-274, 443) (p=0.656) | -386 (-917, 145) (p=0.155) | 223 (-37, 483) (p=0.093) | -86 (-339, 167) (p=0.518) |
| Timor Leste | 1760 (1238, 2283) (p<0.001) | 1022 (586, 1458) (p<0.001) | 917 (562, 1271) (p<0.001) | 434 (138, 730) (p=0.004) | 547 (115, 979) (p=0.013) |
| Togo | 272 (-751, 1295) (p=0.614) | -142 (-1565, 1280) (p=0.855) | -618 (-1579, 343) (p=0.209) | 442 (-201, 1085) (p=0.178) | 64 (-85, 213)  (p=0.407) |
| Uganda | 990 (709, 1271) (p<0.001) | 231 (-447, 910) (p=0.514) | 1090 (438, 1742) (p=0.001) | -2 (-820, 815) (p=0.996) | 518 (317, 719) (p<0.001) |
| Zambia | 495 (422, 567) (p<0.001) | 265 (207, 323) (p<0.001) | 179 (125, 233) (p<0.001) | 108 (78, 138)  (p<0.001) | 71 (56, 85)  (p<0.001) |
| Zimbabwe | 872 (655, 1088) (p<0.001) | 483 (294, 673) (p<0.001) | 665 (430, 900) (p<0.001) | 301 (170, 432) (p<0.001) | 77 (-18, 172)  (p=0.111) |

**Table S12.** Postnatal care utilization rate change (per 10,000) (with 95% confidence interval and p value) associated with achieving recommended antenatal care visits and quality.

| **Country** | **Poorest** | **Poorer** | **Middle** | **Richer** | **Richest** |
| --- | --- | --- | --- | --- | --- |
| Angola | 837 (703, 971) (p<0.001) | 478 (333, 623) (p<0.001) | 368 (290, 447) (p<0.001) | 98 (64, 131)  (p<0.001) | 73 (36, 109)  (p<0.001) |
| Bangladesh | 1850 (1656, 2043) (p<0.001) | 1609 (1424, 1794) (p<0.001) | 1064 (887, 1240) (p<0.001) | 770 (659, 881) (p<0.001) | 289 (213, 364) (p<0.001) |
| Benin | 1205 (1075, 1336) (p<0.001) | 638 (534, 743) (p<0.001) | 449 (320, 579) (p<0.001) | 262 (212, 312) (p<0.001) | 101 (73, 128)  (p<0.001) |
| Burkina Faso | 1578 (1111, 2044) (p<0.001) | 652 (190, 1113) (p=0.006) | 1186 (830, 1543) (p<0.001) | -245 (-625, 135) (p=0.207) | -357 (-557, -157) (p<0.001) |
| Burundi | 61 (-234, 357) (p=0.698) | -87 (-303, 129) (p=0.438) | -59 (-320, 201) (p=0.668) | -13 (-363, 337) (p=0.947) | 203 (-55, 462) (p=0.123) |
| Cambodia | 802 (592, 1011) (p<0.001) | 799 (624, 974) (p<0.001) | 927 (677, 1177) (p<0.001) | 489 (262, 716) (p<0.001) | 205 (60, 349)  (p=0.006) |
| Cameroon | 833 (663, 1003) (p<0.001) | 590 (473, 707) (p<0.001) | 282 (179, 385) (p<0.001) | 193 (109, 277) (p<0.001) | 54 (33, 75)  (p<0.001) |
| Chad | 1811 (1501, 2121) (p<0.001) | 1488 (1170, 1806) (p<0.001) | 1311 (949, 1673) (p<0.001) | 1193 (864, 1523) (p<0.001) | 913 (755, 1070) (p<0.001) |
| Comoros | 652 (272, 1032) (p<0.001) | 246 (-38, 529) (p=0.089) | -23 (-265, 219) (p=0.862) | 23 (-264, 310) (p=0.883) | 78 (-207, 362) (p=0.605) |
| Congo | 752 (510, 994) (p<0.001) | 329 (127, 530) (p=0.001) | 127 (-20, 274)  (p=0.09) | 24 (-2, 51)  (p=0.07) | -3 (-19, 13)  (p=0.729) |
| Congo Democratic Republic | 884 (571, 1196) (p<0.001) | 590 (306, 874) (p<0.001) | 570 (377, 762) (p<0.001) | 315 (125, 504) (p=0.001) | 263 (182, 343) (p<0.001) |
| Cote d'Ivoire | 1723 (1379, 2068) (p<0.001) | 988 (702, 1274) (p<0.001) | 385 (1, 770)  (p=0.049) | 185 (-8, 378)  (p=0.06) | 107 (-94, 307) (p=0.301) |
| Dominican Republic | 132 (73, 190)  (p<0.001) | 50 (-13, 113)  (p=0.12) | 39 (0, 79)  (p=0.049) | 7 (-2, 16)  (p=0.106) | 1 (-2, 5)  (p=0.47) |
| Egypt | 19 (-87, 125)  (p=0.738) | 32 (-59, 123)  (p=0.498) | 92 (-36, 220)  (p=0.159) | 61 (-2, 124)  (p=0.059) | 77 (29, 125)  (p=0.002) |
| Ethiopia | 684 (487, 882) (p<0.001) | 768 (531, 1005) (p<0.001) | 668 (460, 875) (p<0.001) | 811 (489, 1133) (p<0.001) | 320 (220, 420) (p<0.001) |
| Gabon | 524 (414, 634) (p<0.001) | 213 (89, 337)  (p<0.001) | 111 (50, 172)  (p<0.001) | 78 (19, 137)  (p=0.009) | 33 (1, 64)  (p=0.04) |
| Gambia | 151 (121, 180) (p<0.001) | 163 (99, 227)  (p<0.001) | 136 (44, 227)  (p=0.004) | -164 (-337, 9) (p=0.063) | 195 (67, 322)  (p=0.003) |
| Ghana | 298 (169, 427) (p<0.001) | 164 (41, 287)  (p=0.009) | 70 (16, 125)  (p=0.011) | 27 (5, 49)  (p=0.017) | 5 (-10, 20)  (p=0.498) |
| Guatemala | 229 (114, 345) (p<0.001) | 252 (152, 353) (p<0.001) | 114 (52, 175)  (p<0.001) | 108 (56, 160)  (p<0.001) | 25 (-14, 63)  (p=0.215) |
| Guinea | 1182 (775, 1588) (p<0.001) | 1396 (854, 1938) (p<0.001) | 196 (-204, 595) (p=0.342) | 502 (313, 690) (p<0.001) | 218 (99, 336)  (p<0.001) |
| Haiti | 1152 (987, 1317) (p<0.001) | 591 (408, 774) (p<0.001) | 529 (395, 663) (p<0.001) | 341 (225, 458) (p<0.001) | 135 (81, 189)  (p<0.001) |
| Honduras | 361 (280, 443) (p<0.001) | 172 (112, 233) (p<0.001) | 61 (37, 86)  (p<0.001) | 30 (12, 48)  (p=0.001) | 10 (2, 18)  (p=0.012) |
| India | 436 (415, 458) (p<0.001) | 276 (260, 293) (p<0.001) | 163 (150, 177) (p<0.001) | 128 (115, 141) (p<0.001) | 89 (80, 98)  (p<0.001) |
| Jordan | 88 (20, 156)  (p=0.011) | -32 (-120, 56) (p=0.485) | 40 (-17, 98)  (p=0.17) | 44 (3, 84)  (p=0.033) | 56 (10, 102)  (p=0.017) |
| Kenya | 146 (55, 237)  (p=0.002) | -67 (-188, 55) (p=0.288) | -36 (-123, 52) (p=0.434) | 28 (-34, 90)  (p=0.38) | 5 (-19, 28)  (p=0.706) |
| Lesotho | 481 (258, 704) (p<0.001) | 410 (239, 581) (p<0.001) | 339 (145, 533) (p<0.001) | 296 (127, 466) (p<0.001) | 149 (51, 248)  (p=0.003) |
| Liberia | 127 (17, 238)  (p=0.024) | -80 (-216, 55) (p=0.247) | -43 (-162, 75) (p=0.483) | 0 (-44, 44)  (p=0.997) | -44 (-146, 57) (p=0.398) |
| Madagascar | 970 (579, 1361) (p<0.001) | 832 (122, 1541) (p=0.021) | 522 (32, 1013) (p=0.036) | 1059 (553, 1566) (p<0.001) | 437 (240, 633) (p<0.001) |
| Malawi | 797 (409, 1184) (p<0.001) | 523 (164, 881) (p=0.004) | 449 (14, 884)  (p=0.043) | 241 (-228, 710) (p=0.319) | 588 (267, 909) (p<0.001) |
| Maldives | 22 (-5, 49)  (p=0.117) | 14 (-4, 31)  (p=0.118) | 6 (-13, 26)  (p=0.549) | 18 (-8, 45)  (p=0.172) | 2 (-5, 9)  (p=0.58) |
| Mali | 1114 (703, 1524) (p<0.001) | 566 (217, 915) (p=0.002) | 286 (-83, 656) (p=0.129) | 278 (58, 498)  (p=0.013) | 199 (63, 334)  (p=0.004) |
| Mauritania | 918 (546, 1290) (p<0.001) | 577 (239, 914) (p<0.001) | 448 (253, 643) (p<0.001) | 374 (221, 527) (p<0.001) | 145 (21, 268)  (p=0.021) |
| Myanmar | 1331 (1036, 1627) (p<0.001) | 831 (466, 1196) (p<0.001) | 470 (92, 847)  (p=0.015) | 441 (270, 612) (p<0.001) | 84 (-41, 208)  (p=0.188) |
| Nepal | 573 (318, 829) (p<0.001) | -93 (-413, 227) (p=0.582) | 28 (-214, 271) (p=0.831) | -163 (-368, 43) (p=0.12) | -46 (-152, 61) (p=0.408) |
| Niger | 1043 (113, 1972) (p=0.028) | 1015 (179, 1851) (p=0.017) | 1009 (206, 1812) (p=0.014) | 956 (377, 1534) (p=0.001) | 338 (184, 493) (p<0.001) |
| Nigeria | 1057 (909, 1205) (p<0.001) | 750 (616, 885) (p<0.001) | 531 (432, 629) (p<0.001) | 304 (253, 355) (p<0.001) | 130 (81, 179)  (p<0.001) |
| Pakistan | 352 (54, 649)  (p=0.02) | 439 (95, 783)  (p=0.012) | 145 (-5, 294)  (p=0.057) | 106 (-11, 223) (p=0.075) | 159 (27, 291)  (p=0.018) |
| Rwanda | 594 (408, 781) (p<0.001) | 448 (276, 620) (p<0.001) | 191 (-116, 499) (p=0.224) | 281 (57, 504)  (p=0.014) | 43 (-167, 253) (p=0.701) |
| Sierra Leone | 54 (-49, 158)  (p=0.31) | 22 (-124, 168) (p=0.781) | 41 (-33, 115)  (p=0.281) | -36 (-109, 37) (p=0.339) | 56 (-6, 118)  (p=0.075) |
| South Africa | 116 (63, 168)  (p<0.001) | 179 (72, 287)  (p=0.001) | 61 (19, 103)  (p=0.005) | 33 (-42, 108)  (p=0.396) | 50 (-3, 104)  (p=0.066) |
| Tanzania | -540 (-945, -136) (p=0.009) | -729 (-1159, -298) (p<0.001) | -805 (-1279, -331) (p<0.001) | -839 (-1214, -465) (p<0.001) | -889 (-1175, -604) (p<0.001) |
| Timor Leste | 350 (89, 611)  (p=0.009) | 190 (-55, 435) (p=0.128) | -91 (-298, 117) (p=0.398) | -205 (-434, 23) (p=0.078) | -177 (-430, 77) (p=0.174) |
| Togo | 906 (160, 1653) (p=0.017) | 880 (112, 1648) (p=0.024) | 292 (-182, 765) (p=0.23) | -12 (-307, 282) (p=0.94) | -30 (-204, 143) (p=0.744) |
| Uganda | 162 (-200, 524) (p=0.387) | 268 (-216, 753) (p=0.281) | 696 (339, 1054) (p<0.001) | 439 (67, 810)  (p=0.021) | 596 (310, 882) (p<0.001) |
| Zambia | 657 (531, 783) (p<0.001) | 327 (185, 470) (p<0.001) | 98 (-79, 275)  (p=0.28) | -192 (-371, -13) (p=0.035) | -155 (-239, -71) (p<0.001) |
| Zimbabwe | 1207 (1050, 1365) (p<0.001) | 916 (767, 1064) (p<0.001) | 862 (721, 1002) (p<0.001) | 481 (382, 580) (p<0.001) | 246 (189, 302) (p<0.001) |

**Table S13.** Stunting rate change (per 10,000) (with 95% confidence interval and p value) associated with recommended antenatal care visits and quality.

| **Country** | **Poorest** | **Poorer** | **Middle** | **Richer** | **Richest** |
| --- | --- | --- | --- | --- | --- |
| Bangladesh | -672 (-808, -535) (p<0.001) | -554 (-675, -433) (p<0.001) | -473 (-568, -378) (p<0.001) | -335 (-414, -255) (p<0.001) | -159 (-197, -121) (p<0.001) |
| Benin | -124 (-178, -71) (p<0.001) | -81 (-116, -46) (p<0.001) | -65 (-93, -37)  (p<0.001) | -36 (-52, -21)  (p<0.001) | -18 (-27, -9)  (p<0.001) |
| Burkina Faso | -427 (-793, -60) (p=0.022) | -747 (-1483, -10) (p=0.047) | 90 (-587, 768) (p=0.806) | -342 (-629, -56) (p=0.019) | -146 (-272, -21) (p=0.022) |
| Burundi | -123 (-195, -51) (p<0.001) | 671 (67, 1276) (p=0.029) | -275 (-451, -99) (p=0.002) | -354 (-868, 160) (p=0.178) | -485 (-743, -226) (p<0.001) |
| Cambodia | -502 (-649, -356) (p<0.001) | -374 (-498, -249) (p<0.001) | -356 (-474, -237) (p<0.001) | -218 (-434, -1) (p=0.048) | -142 (-194, -90) (p<0.001) |
| Cameroon | -110 (-211, -9) (p=0.033) | -41 (-200, 117) (p=0.623) | -116 (-179, -53) (p<0.001) | -52 (-112, 9)  (p=0.095) | -8 (-39, 24)  (p=0.65) |
| Chad | -636 (-823, -448) (p<0.001) | -527 (-925, -130) (p=0.009) | -359 (-598, -120) (p=0.003) | -294 (-660, 73) (p=0.116) | -157 (-331, 17) (p=0.076) |
| Comoros | -102 (-150, -55) (p<0.001) | -275 (-475, -75) (p=0.007) | -34 (-52, -15)  (p<0.001) | -28 (-143, 86) (p=0.639) | -8 (-76, 61)  (p=0.837) |
| Congo | -122 (-176, -68) (p<0.001) | -81 (-188, 27) (p=0.141) | -89 (-146, -33) (p=0.002) | -5 (-8, -2)  (p=0.004) | -11 (-24, 2)  (p=0.098) |
| Congo Democratic Republic | -160 (-236, -84) (p<0.001) | -107 (-362, 149) (p=0.42) | 135 (-258, 529) (p=0.511) | 95 (-78, 268)  (p=0.286) | -137 (-246, -27) (p=0.014) |
| Cote d'Ivoire | -121 (-177, -66) (p<0.001) | -24 (-192, 144) (p=0.794) | -412 (-695, -129) (p=0.004) | -228 (-453, -3) (p=0.047) | -48 (-77, -18)  (p=0.002) |
| Dominican Republic | -36 (-48, -23)  (p<0.001) | -13 (-22, -5)  (p=0.002) | -4 (-13, 6)  (p=0.452) | -4 (-9, 1)  (p=0.12) | -3 (-9, 2)  (p=0.241) |
| Egypt | -73 (-154, 8)  (p=0.077) | 12 (-67, 91)  (p=0.78) | 14 (-35, 62)  (p=0.595) | 33 (-51, 117)  (p=0.447) | 60 (38, 82)  (p<0.001) |
| Ethiopia | -534 (-772, -295) (p<0.001) | -277 (-596, 42) (p=0.088) | -62 (-475, 350) (p=0.781) | -182 (-529, 164) (p=0.306) | -228 (-327, -129) (p<0.001) |
| Gabon | -55 (-81, -29)  (p<0.001) | -36 (-71, -1)  (p=0.046) | -38 (-96, 21)  (p=0.212) | -3 (-64, 57)  (p=0.926) | -1 (-2, 0)  (p=0.037) |
| Gambia | -12 (-19, -6)  (p<0.001) | -26 (-50, -2)  (p=0.037) | -1 (-23, 22)  (p=0.956) | -8 (-13, -4)  (p<0.001) | -6 (-10, -2)  (p=0.002) |
| Ghana | -25 (-37, -13)  (p<0.001) | 5 (-33, 42)  (p=0.824) | -15 (-33, 3)  (p=0.094) | -1 (-4, 1)  (p=0.301) | -2 (-6, 2)  (p=0.304) |
| Guatemala | -366 (-542, -190) (p<0.001) | -395 (-520, -270) (p<0.001) | -245 (-393, -97) (p=0.001) | -131 (-234, -28) (p=0.013) | -45 (-78, -13)  (p=0.007) |
| Guinea | -413 (-715, -110) (p=0.008) | 247 (-80, 575) (p=0.139) | -290 (-507, -73) (p=0.009) | -105 (-213, 2) (p=0.055) | -78 (-128, -28) (p=0.002) |
| Haiti | -346 (-475, -218) (p<0.001) | -267 (-363, -172) (p<0.001) | -135 (-247, -23) (p=0.018) | -78 (-115, -41) (p<0.001) | -42 (-60, -23)  (p<0.001) |
| Honduras | -135 (-167, -103) (p<0.001) | -162 (-244, -80) (p<0.001) | -65 (-135, 5)  (p=0.07) | -57 (-116, 3)  (p=0.062) | -27 (-42, -13)  (p<0.001) |
| India | -47 (-61, -33)  (p<0.001) | -22 (-30, -13)  (p<0.001) | -18 (-24, -12)  (p<0.001) | -2 (-11, 8)  (p=0.726) | 3 (-4, 10)  (p=0.464) |
| Kenya | -151 (-223, -80) (p<0.001) | -96 (-140, -52) (p<0.001) | -107 (-152, -62) (p<0.001) | -76 (-106, -46) (p<0.001) | -15 (-24, -6)  (p=0.002) |
| Lesotho | -30 (-45, -15)  (p<0.001) | -53 (-404, 298) (p=0.78) | 49 (-253, 351) (p=0.763) | -51 (-94, -7)  (p=0.023) | -46 (-82, -11)  (p=0.01) |
| Liberia | -69 (-97, -41)  (p<0.001) | 5 (-103, 113)  (p=0.933) | -44 (-65, -22)  (p<0.001) | -30 (-49, -11)  (p=0.002) | -12 (-21, -4)  (p=0.003) |
| Madagascar | 153 (-226, 531) (p=0.437) | -279 (-727, 168) (p=0.223) | 207 (-60, 474) (p=0.128) | 122 (-133, 378) (p=0.353) | 114 (-121, 348) (p=0.347) |
| Malawi | 42 (-155, 239)  (p=0.69) | 42 (-159, 244) (p=0.694) | -139 (-400, 122) (p=0.302) | -93 (-263, 78) (p=0.289) | 572 (235, 909) (p<0.001) |
| Maldives | -17 (-27, -8)  (p<0.001) | -9 (-15, -4)  (p=0.001) | -5 (-14, 3)  (p=0.23) | -8 (-23, 7)  (p=0.307) | 1 (-5, 7)  (p=0.771) |
| Mali | -66 (-483, 350) (p=0.768) | -319 (-739, 101) (p=0.136) | -551 (-875, -227) (p<0.001) | -422 (-610, -235) (p<0.001) | -56 (-169, 56)  (p=0.33) |
| Mauritania | -203 (-534, 129) (p=0.233) | -642 (-1040, -244) (p=0.002) | -599 (-839, -359) (p<0.001) | -60 (-183, 64) (p=0.349) | -28 (-92, 36)  (p=0.394) |
| Mozambique | -140 (-207, -74) (p<0.001) | 59 (-309, 428) (p=0.765) | 156 (-204, 517) (p=0.403) | -81 (-121, -40) (p<0.001) | -64 (-256, 128) (p=0.526) |
| Myanmar | -447 (-566, -327) (p<0.001) | 37 (-398, 472) (p=0.877) | -236 (-347, -126) (p<0.001) | -52 (-177, 72)  (p=0.42) | -73 (-131, -14) (p=0.015) |
| Nepal | -703 (-882, -524) (p<0.001) | -720 (-1006, -434) (p<0.001) | -457 (-668, -247) (p<0.001) | -250 (-337, -163) (p<0.001) | -93 (-127, -59) (p<0.001) |
| Niger | -101 (-314, 111) (p=0.355) | -697 (-1456, 62) (p=0.071) | -88 (-285, 109) (p=0.387) | 391 (15, 767)  (p=0.041) | 38 (-248, 323) (p=0.807) |
| Nigeria | -179 (-338, -21) (p=0.026) | -328 (-446, -210) (p<0.001) | -117 (-201, -32) (p=0.007) | 25 (-39, 90)  (p=0.449) | -11 (-23, 0)  (p=0.056) |
| Pakistan | -256 (-478, -33) (p=0.024) | -408 (-630, -186) (p<0.001) | -235 (-357, -113) (p<0.001) | -219 (-328, -111) (p<0.001) | -32 (-65, 0)  (p=0.051) |
| Rwanda | -187 (-399, 25) (p=0.083) | -176 (-381, 30) (p=0.093) | -438 (-652, -224) (p<0.001) | -529 (-804, -254) (p<0.001) | -330 (-527, -134) (p=0.001) |
| Sierra Leone | -43 (-75, -11)  (p=0.009) | -39 (-67, -11)  (p=0.006) | -20 (-50, 11)  (p=0.207) | -27 (-54, -1)  (p=0.045) | -28 (-43, -13)  (p<0.001) |
| South Africa | -11 (-19, -2)  (p=0.012) | -54 (-190, 81)  (p=0.44) | -17 (-38, 5)  (p=0.127) | -8 (-110, 93)  (p=0.884) | -33 (-101, 34) (p=0.337) |
| Tanzania | 471 (263, 678) (p<0.001) | 126 (-308, 560) (p=0.581) | 26 (-244, 296) (p=0.861) | 363 (210, 516) (p<0.001) | 80 (-61, 222)  (p=0.269) |
| Timor Leste | 140 (-125, 405) (p=0.304) | -358 (-785, 68)  (p=0.1) | 578 (238, 918) (p<0.001) | -25 (-271, 220) (p=0.85) | 121 (-42, 285) (p=0.147) |
| Togo | 545 (-324, 1414) (p=0.221) | -51 (-992, 891) (p=0.923) | 108 (-392, 609) (p=0.684) | -187 (-335, -38) (p=0.013) | 28 (-17, 72)  (p=0.229) |
| Uganda | -119 (-193, -45) (p=0.002) | -103 (-169, -37) (p=0.002) | -598 (-1034, -162) (p=0.007) | -83 (-136, -31) (p=0.002) | -162 (-268, -57) (p=0.003) |
| Zambia | -67 (-126, -8)  (p=0.026) | 119 (-26, 265) (p=0.108) | -41 (-85, 3)  (p=0.065) | -26 (-171, 119) (p=0.739) | -116 (-200, -32) (p=0.007) |
| Zimbabwe | -132 (-186, -78) (p<0.001) | -146 (-245, -46) (p=0.004) | 50 (-81, 180)  (p=0.463) | -85 (-119, -52) (p<0.001) | -34 (-63, -5)  (p=0.021) |

**Table S14.** Wasting rate change (per 10,000) (with 95% confidence interval) and p value associated with recommended antenatal care visits and quality.

| **Country** | **Poorest** | **Poorer** | **Middle** | **Richer** | **Richest** |
| --- | --- | --- | --- | --- | --- |
| Bangladesh | -5 (-59, 50)  (p=0.873) | -3 (-47, 40)  (p=0.889) | -3 (-37, 32)  (p=0.896) | -2 (-26, 23)  (p=0.912) | 0 (-12, 12)  (p=0.997) |
| Benin | -38 (-65, -11)  (p=0.006) | -23 (-40, -7)  (p=0.006) | -19 (-32, -7)  (p=0.003) | -11 (-18, -4)  (p=0.002) | -6 (-9, -2)  (p<0.001) |
| Burkina Faso | -71 (-142, 0)  (p=0.051) | -66 (-133, 1)  (p=0.052) | 231 (-251, 713) (p=0.353) | -413 (-923, 97) (p=0.112) | -86 (-273, 101) (p=0.374) |
| Burundi | 127 (-40, 295) (p=0.136) | 180 (-88, 449) (p=0.189) | 103 (-37, 242)  (p=0.15) | 93 (-35, 221)  (p=0.156) | -5 (-117, 107) (p=0.934) |
| Cambodia | -10 (-39, 18)  (p=0.493) | -11 (-35, 14)  (p=0.395) | -10 (-30, 10)  (p=0.343) | -10 (-26, 7)  (p=0.265) | -6 (-17, 4)  (p=0.252) |
| Cameroon | -213 (-337, -89) (p<0.001) | -104 (-166, -42) (p=0.001) | -88 (-123, -53) (p<0.001) | -13 (-23, -3)  (p=0.014) | -4 (-7, -1)  (p=0.015) |
| Chad | -148 (-358, 62) (p=0.169) | -276 (-524, -29) (p=0.028) | -424 (-661, -188) (p<0.001) | -604 (-915, -293) (p<0.001) | 64 (-18, 145)  (p=0.127) |
| Comoros | 13 (-57, 83)  (p=0.732) | 81 (-95, 257)  (p=0.373) | 25 (-12, 62)  (p=0.186) | -1 (-33, 31)  (p=0.958) | -3 (-20, 14)  (p=0.744) |
| Congo | -13 (-28, 3)  (p=0.105) | -9 (-17, -1)  (p=0.033) | -7 (-12, -3)  (p=0.001) | -2 (-3, -1)  (p=0.008) | 24 (0, 48)  (p=0.053) |
| Congo Democratic Republic | -147 (-285, -8) (p=0.038) | -118 (-241, 4) (p=0.057) | -116 (-236, 4) (p=0.058) | -74 (-149, 0)  (p=0.049) | -85 (-141, -28) (p=0.003) |
| Cote d'Ivoire | -43 (-175, 89) (p=0.532) | -35 (-141, 71) (p=0.527) | 24 (-91, 139)  (p=0.692) | 31 (-81, 142)  (p=0.602) | 3 (-30, 36)  (p=0.872) |
| Dominican Republic | 0 (-1, 1)  (p=0.91) | 0 (0, 0)  (p=0.988) | 0 (0, 0)  (p=0.996) | 0 (0, 0)  (p=0.973) | 0 (0, 0)  (p=0.823) |
| Egypt | -7 (-19, 5)  (p=0.243) | -6 (-15, 4)  (p=0.243) | -5 (-12, 3)  (p=0.213) | -4 (-9, 2)  (p=0.215) | -2 (-5, 1)  (p=0.214) |
| Ethiopia | -199 (-323, -74) (p=0.002) | -147 (-241, -53) (p=0.002) | 83 (-170, 335)  (p=0.53) | -101 (-170, -33) (p=0.004) | -72 (-178, 35) (p=0.187) |
| Gabon | -8 (-13, -2)  (p=0.004) | -3 (-5, -1)  (p<0.001) | -2 (-3, 0)  (p=0.014) | -1 (-3, 0)  (p=0.026) | 2 (-2, 6)  (p=0.346) |
| Gambia | -5 (-7, -2)  (p<0.001) | 26 (7, 44)  (p=0.006) | 10 (-42, 63)  (p=0.714) | -1 (-7, 6)  (p=0.862) | -4 (-5, -2)  (p<0.001) |
| Ghana | -6 (-11, -2)  (p=0.01) | -5 (-8, -2)  (p=0.003) | -11 (-21, -2)  (p=0.018) | 3 (-5, 11)  (p=0.47) | 0 (-1, 0)  (p=0.14) |
| Guatemala | -1 (-3, 1)  (p=0.371) | -1 (-2, 1  ) (p=0.443) | 0 (-1, 1)  (p=0.448) | 0 (-1, 0)  (p=0.491) | 0 (0, 0)  (p=0.498) |
| Guinea | -34 (-71, 3)  (p=0.071) | -100 (-245, 45) (p=0.178) | -104 (-225, 17) (p=0.093) | -15 (-28, -2)  (p=0.025) | -8 (-12, -3)  (p<0.001) |
| Haiti | -1 (-10, 8)  (p=0.798) | -1 (-7, 5)  (p=0.833) | 0 (-4, 3)  (p=0.899) | 0 (-3, 2)  (p=0.921) | 0 (-1, 1)  (p=0.935) |
| Honduras | -1 (-2, 1)  (p=0.49) | 0 (-1, 1)  (p=0.617) | 0 (-1, 0)  (p=0.681) | 0 (0, 0)  (p=0.843) | 0 (0, 0)  (p=0.735) |
| India | 5 (-6, 16)  (p=0.401) | 4 (-2, 11)  (p=0.213) | 4 (-1, 9)  (p=0.128) | 3 (0, 7)  (p=0.082) | 3 (0, 5)  (p=0.052) |
| Kenya | -21 (-37, -5)  (p=0.01) | -8 (-12, -3)  (p<0.001) | -10 (-15, -5)  (p<0.001) | -9 (-12, -5)  (p<0.001) | -4 (-6, -2)  (p<0.001) |
| Lesotho | -1 (-5, 2)  (p=0.399) | 6 (-11, 24)  (p=0.483) | -1 (-4, 1)  (p=0.317) | -2 (-4, 0)  (p=0.033) | -3 (-7, 0)  (p=0.036) |
| Liberia | -4 (-9, 2)  (p=0.211) | -9 (-39, 22)  (p=0.591) | -2 (-5, 2)  (p=0.296) | 8 (-18, 34)  (p=0.558) | 9 (-6, 24)  (p=0.234) |
| Madagascar | 4 (-32, 39)  (p=0.847) | -174 (-356, 8) (p=0.061) | -136 (-258, -15) (p=0.027) | 8 (-6, 22)  (p=0.25) | 33 (-38, 103)  (p=0.373) |
| Malawi | 2 (-6, 11)  (p=0.609) | 3 (-5, 12)  (p=0.445) | 13 (-100, 126) (p=0.832) | 1 (-5, 8)  (p=0.693) | 1 (-4, 6)  (p=0.639) |
| Maldives | 0 (-2, 1)  (p=0.91) | 0 (-1, 1)  (p=0.903) | 0 (-1, 1)  (p=0.959) | 0 (-1, 1)  (p=0.944) | 0 (-1, 0)  (p=0.475) |
| Mali | -303 (-482, -124) (p<0.001) | -254 (-411, -97) (p=0.002) | -329 (-513, -145) (p<0.001) | -217 (-335, -98) (p<0.001) | -50 (-76, -23)  (p<0.001) |
| Mauritania | -26 (-50, -1)  (p=0.039) | 25 (-102, 152) (p=0.713) | 8 (-97, 114)  (p=0.888) | -79 (-202, 45) (p=0.213) | -23 (-33, -13)  (p<0.001) |
| Mozambique | -3 (-29, 23)  (p=0.828) | 1 (-22, 25)  (p=0.91) | -76 (-241, 88) (p=0.369) | -33 (-143, 77) (p=0.567) | 0 (-3, 3)  (p=0.978) |
| Myanmar | -6 (-27, 15)  (p=0.611) | -4 (-20, 11)  (p=0.581) | -4 (-16, 8)  (p=0.513) | -3 (-10, 4)  (p=0.456) | -2 (-5, 2)  (p=0.342) |
| Nepal | -11 (-49, 28)  (p=0.602) | -8 (-38, 21)  (p=0.587) | -6 (-27, 16)  (p=0.615) | -3 (-16, 9)  (p=0.591) | -1 (-6, 3)  (p=0.632) |
| Niger | 36 (-33, 105)  (p=0.307) | 137 (-84, 358) (p=0.225) | 562 (-318, 1442) (p=0.212) | 138 (-30, 307) (p=0.107) | -96 (-234, 42) (p=0.173) |
| Nigeria | -63 (-133, 6)  (p=0.073) | 0 (-113, 113)  (p=0.997) | -22 (-50, 6)  (p=0.117) | -12 (-26, 1)  (p=0.072) | 1 (-12, 14)  (p=0.903) |
| Pakistan | -7 (-34, 20)  (p=0.634) | -5 (-24, 14)  (p=0.622) | -3 (-15, 9)  (p=0.67) | -1 (-9, 6)  (p=0.741) | -1 (-4, 2)  (p=0.561) |
| Rwanda | -3 (-7, 1)  (p=0.101) | -2 (-6, 1)  (p=0.157) | -2 (-5, 1)  (p=0.224) | -43 (-70, -16)  (p=0.002) | -3 (-4, -1)  (p=0.001) |
| Sierra Leone | 4 (-3, 12)  (p=0.258) | 15 (-54, 84)  (p=0.682) | 2 (-2, 7)  (p=0.349) | 47 (16, 78)  (p=0.003) | 4 (-12, 19)  (p=0.66) |
| South Africa | -3 (-5, -1)  (p=0.002) | -2 (-4, -1)  (p=0.008) | -2 (-3, -1)  (p=0.003) | -1 (-3, 0)  (p=0.038) | -2 (-4, 0)  (p=0.075) |
| Tanzania | 151 (-5, 306)  (p=0.057) | 66 (-129, 262) (p=0.516) | 147 (-47, 342) (p=0.138) | 104 (-15, 222) (p=0.086) | 137 (40, 235)  (p=0.006) |
| Timor Leste | -23 (-79, 33)  (p=0.435) | -20 (-70, 30)  (p=0.434) | -22 (-67, 23)  (p=0.349) | -19 (-55, 16)  (p=0.283) | -17 (-45, 12)  (p=0.25) |
| Togo | -45 (-88, -1)  (p=0.043) | -323 (-617, -29) (p=0.031) | -29 (-56, -2)  (p=0.034) | -14 (-25, -2)  (p=0.019) | -7 (-11, -3)  (p<0.001) |
| Uganda | 37 (-20, 93)  (p=0.203) | 32 (-17, 81)  (p=0.202) | 33 (-23, 89)  (p=0.254) | -89 (-133, -45) (p<0.001) | 11 (-7, 29)  (p=0.232) |
| Zambia | 2 (-10, 13)  (p=0.809) | 1 (-8, 10)  (p=0.833) | -26 (-49, -3)  (p=0.025) | 31 (3, 59)  (p=0.029) | 2 (-53, 57)  (p=0.947) |
| Zimbabwe | 1 (-8, 9)  (p=0.878) | 20 (-31, 70)  (p=0.454) | -15 (-33, 3)  (p=0.094) | -2 (-6, 1)  (p=0.225) | 0 (-1, 1)  (p=0.581) |


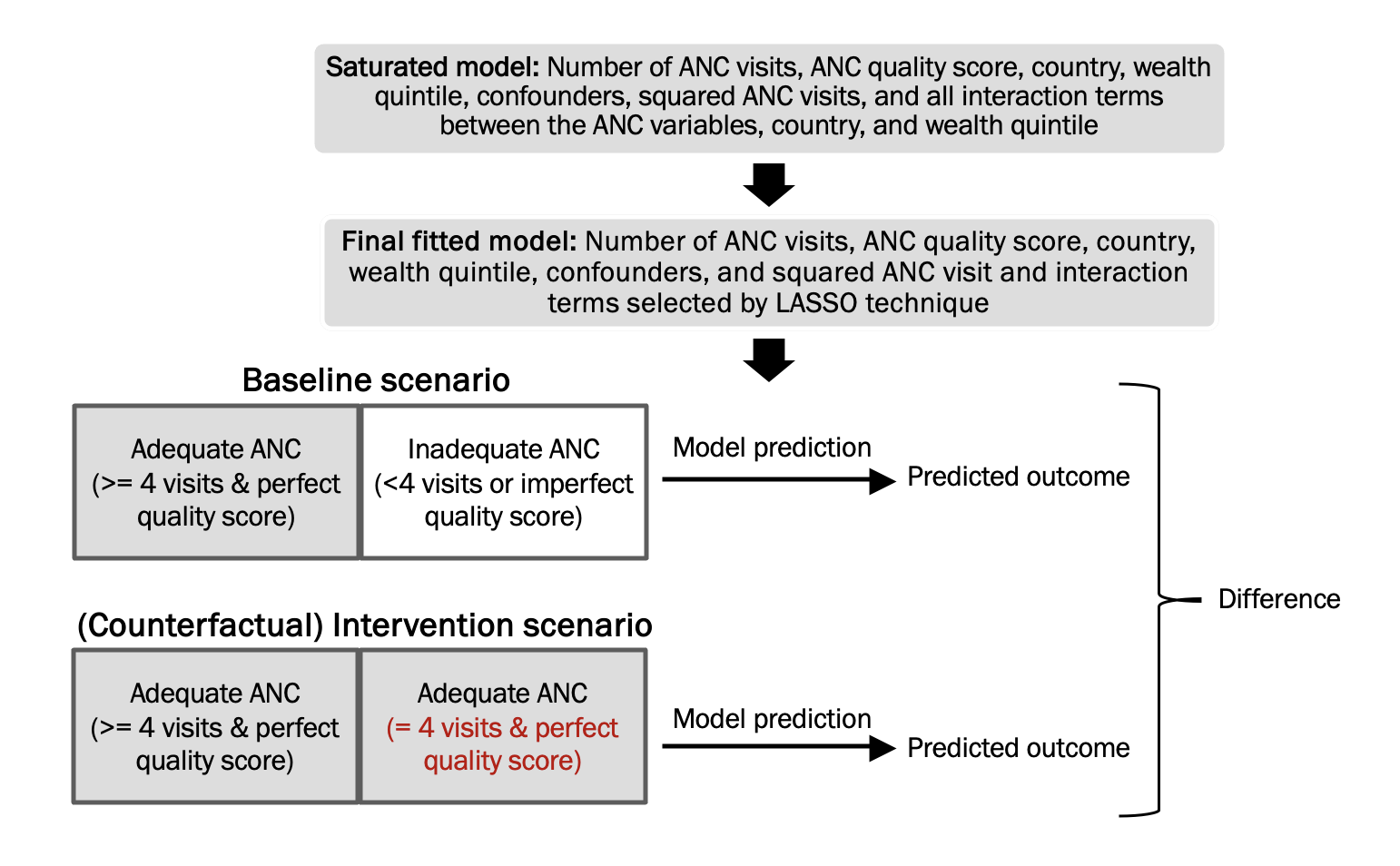


**Figure S1.** The process of statistical analysis.

**
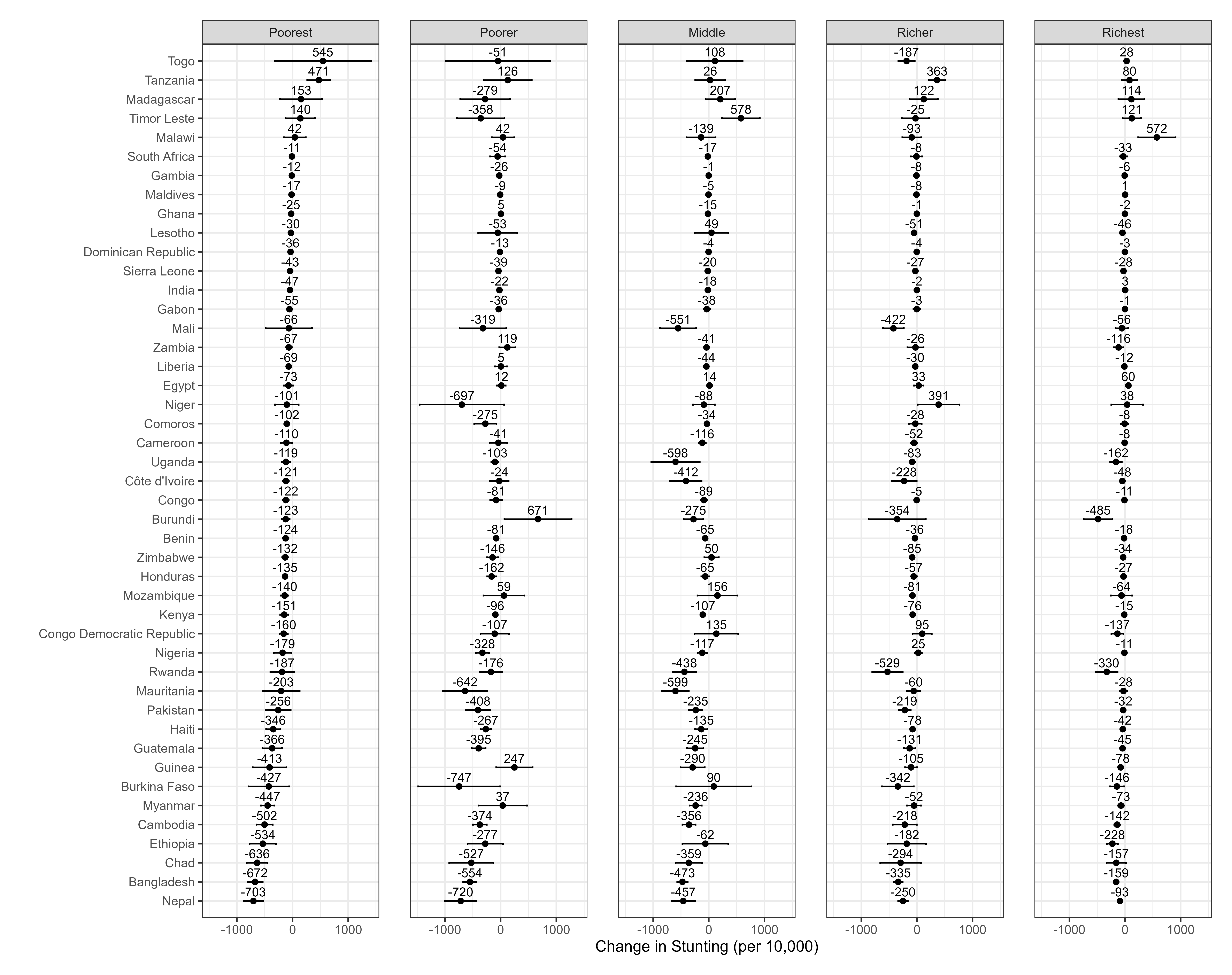
**

**Figure S2**. Stunting rate change associated with achieving recommended antenatal care visits and quality across five wealth quintiles and countries. This change is measured by the predicted difference in facility birth rate between two scenarios: an intervention that ensures all women achieve the adequate level of ANC utilization and quality, and a baseline scenario that represents the current state of ANC in each country. The dots represent point estimates of the changes, and the lines around the dots denote the 95% confidence intervals.


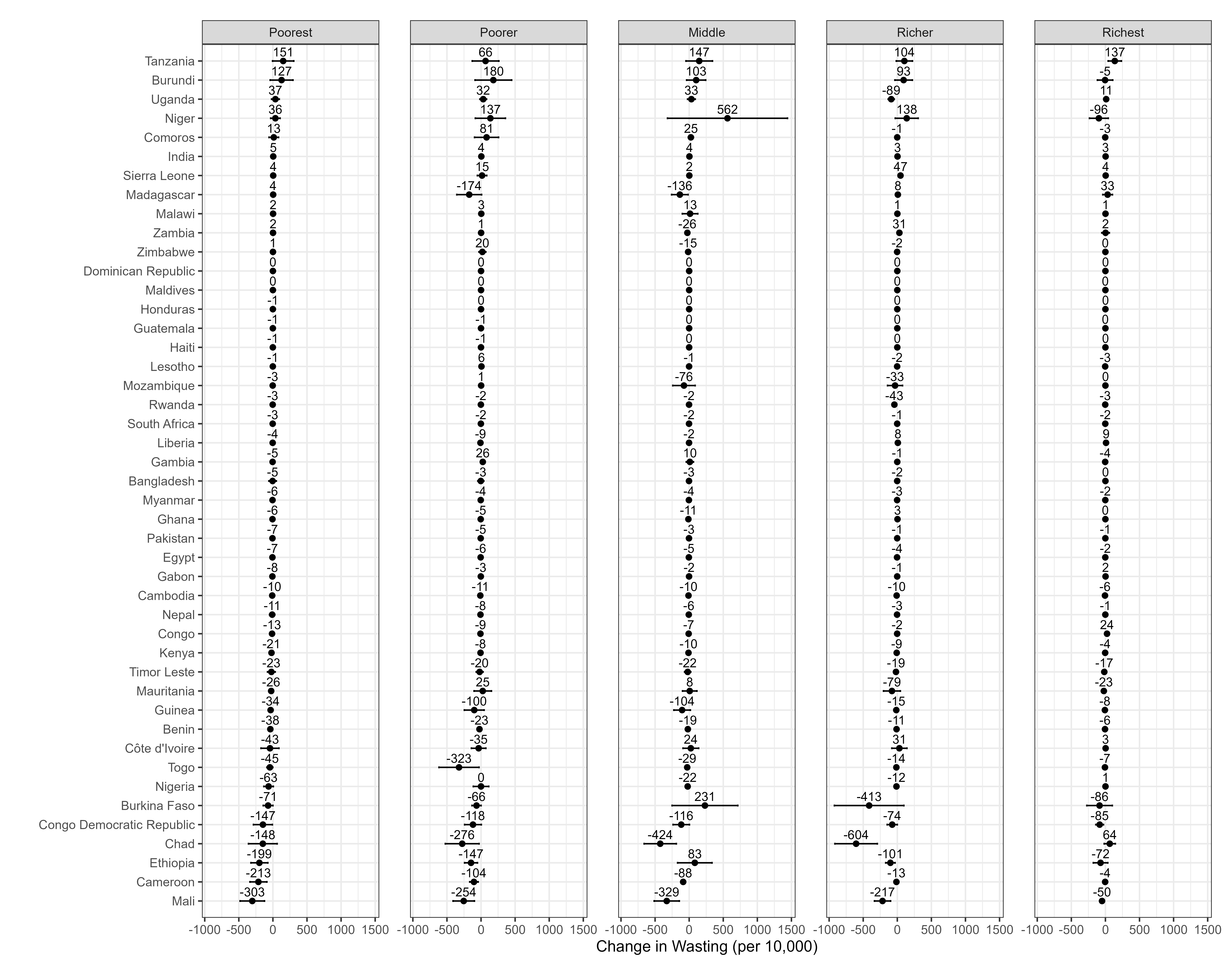


**Figure S3**. Wasting birth rate change associated with achieving recommended antenatal care visits and quality across five wealth quintiles and countries. This change is measured by the predicted difference in facility birth rate between two scenarios: an intervention that ensures all women achieve the adequate level of ANC utilization and quality, and a baseline scenario that represents the current state of ANC in each country. The dots represent point estimates of the changes, and the lines around the dots denote the 95% confidence intervals.

**S1 STROBE Statement—checklist of items that should be included in reports of observational studies**

|  | Item No. | Recommendation |  | Relevant text from manuscript |
| --- | --- | --- | --- | --- |
| **Title and abstract** | 1 | (*a*) Indicate the study’s design with a commonly used term in the title or the abstract |  | Title of manuscript and Methods section of the Abstract |
|  |  | (*b*) Provide in the abstract an informative and balanced summary of what was done and what was found |  |  |
| Introduction | | | |  |
| Background/rationale | 2 | Explain the scientific background and rationale for the investigation being reported |  | Introduction section |
| Objectives | 3 | State specific objectives, including any prespecified hypotheses |  | Last paragraph of Introduction section |
| Methods | | | |  |
| Study design | 4 | Present key elements of study design early in the paper |  | Methods section |
| Setting | 5 | Describe the setting, locations, and relevant dates, including periods of recruitment, exposure, follow-up, and data collection |  | Methods section: Data sources |
| Participants | 6 | (*a*) *Cohort study*—Give the eligibility criteria, and the sources and methods of selection of participants. Describe methods of follow-up  *Case-control study*—Give the eligibility criteria, and the sources and methods of case ascertainment and control selection. Give the rationale for the choice of cases and controls  *Cross-sectional study*—Give the eligibility criteria, and the sources and methods of selection of participants |  | Methods section: Data sources |
|  |  | (*b*) *Cohort study*—For matched studies, give matching criteria and number of exposed and unexposed  *Case-control study*—For matched studies, give matching criteria and the number of controls per case |  |  |
| Variables | 7 | Clearly define all outcomes, exposures, predictors, potential confounders, and effect modifiers. Give diagnostic criteria, if applicable |  | Methods section: Independent variables and dependent variables |
| Data sources/ measurement | 8* | For each variable of interest, give sources of data and details of methods of assessment (measurement). Describe comparability of assessment methods if there is more than one group |  | Methods section: Independent variables and dependent variables |
| Bias | 9 | Describe any efforts to address potential sources of bias |  | First paragraph of Results section |
| Study size | 10 | Explain how the study size was arrived at |  | First paragraph of Results section |

Continued on next page

| Quantitative variables | 11 | Explain how quantitative variables were handled in the analyses. If applicable, describe which groupings were chosen and why |  | Methods section: Independent variables and dependent variables |
| --- | --- | --- | --- | --- |
| Statistical methods | 12 | (*a*) Describe all statistical methods, including those used to control for confounding |  | Methods section: Statistical analysis |
|  |  | (*b*) Describe any methods used to examine subgroups and interactions |  | Methods section: Statistical analysis |
|  |  | (*c*) Explain how missing data were addressed |  | First paragraph of Results section |
|  |  | (*d*) *Cohort study*—If applicable, explain how loss to follow-up was addressed  *Case-control study*—If applicable, explain how matching of cases and controls was addressed  *Cross-sectional study*—If applicable, describe analytical methods taking account of sampling strategy |  | Last paragraph of statistical analysis, Methods section |
|  |  | (*e*) Describe any sensitivity analyses |  |  |
| Results | | | | |
| Participants | 13* | (a) Report numbers of individuals at each stage of study—eg numbers potentially eligible, examined for eligibility, confirmed eligible, included in the study, completing follow-up, and analysed |  | First paragraph of Results section |
|  |  | (b) Give reasons for non-participation at each stage |  |  |
|  |  | (c) Consider use of a flow diagram |  |  |
| Descriptive data | 14* | (a) Give characteristics of study participants (eg demographic, clinical, social) and information on exposures and potential confounders |  | First paragraph of Results section; Table 1-2, and Appendix and S2 |
|  |  | (b) Indicate number of participants with missing data for each variable of interest |  | First paragraph of Results section |
|  |  | (c) *Cohort study*—Summarise follow-up time (eg, average and total amount) |  |  |
| Outcome data | 15* | *Cohort study*—Report numbers of outcome events or summary measures over time |  |  |
|  |  | *Case-control study—*Report numbers in each exposure category, or summary measures of exposure |  |  |
|  |  | *Cross-sectional study—*Report numbers of outcome events or summary measures |  | First and second paragraphs of Results section |
| Main results | 16 | (*a*) Give unadjusted estimates and, if applicable, confounder-adjusted estimates and their precision (eg, 95% confidence interval). Make clear which confounders were adjusted for and why they were included |  | Paragraph 3-7 of Results section; Figure 1-4 |
|  |  | (*b*) Report category boundaries when continuous variables were categorized |  |  |
|  |  | (*c*) If relevant, consider translating estimates of relative risk into absolute risk for a meaningful time period |  | Paragraph 3-7 of Results section; Figure 1-4 |

Continued on next page

| Other analyses | 17 | Report other analyses done—eg analyses of subgroups and interactions, and sensitivity analyses |  |  |
| --- | --- | --- | --- | --- |
| Discussion | | | | |
| Key results | 18 | Summarise key results with reference to study objectives |  | First paragraph of Discussion section |
| Limitations | 19 | Discuss limitations of the study, taking into account sources of potential bias or imprecision. Discuss both direction and magnitude of any potential bias |  | Seventh paragraph of Discussion section |
| Interpretation | 20 | Give a cautious overall interpretation of results considering objectives, limitations, multiplicity of analyses, results from similar studies, and other relevant evidence |  | Discussion section |
| Generalisability | 21 | Discuss the generalisability (external validity) of the study results |  | Seventh paragraph of Discussion section |
| Other information | |  | | |
| Funding | 22 | Give the source of funding and the role of the funders for the present study and, if applicable, for the original study on which the present article is based |  | Acknowledgement |

*Give information separately for cases and controls in case-control studies and, if applicable, for exposed and unexposed groups in cohort and cross-sectional studies.

**Note:** An Explanation and Elaboration article discusses each checklist item and gives methodological background and published examples of transparent reporting. The STROBE checklist is best used in conjunction with this article (freely available on the Web sites of PLoS Medicine at http://www.plosmedicine.org/, Annals of Internal Medicine at http://www.annals.org/, and Epidemiology at http://www.epidem.com/). Information on the STROBE Initiative is available at www.strobe-statement.org.
